# Inner- and Second-Sphere Interactions of Co(II) and Fe(II) Parashift Agents with Lactate, Trifluorolactate, and Fluoride

**DOI:** 10.1021/acs.inorgchem.6c01979

**Published:** 2026-06-15

**Authors:** Anwita Roy, Deepak Krishnan Balaji, Joseph A. Spernyak, Janet R. Morrow

**Affiliations:** † Department of Chemistry, 12292 University at Buffalo, The State University of New York, Amherst, New York 14260, United States; ‡ Department of Cell Stress Biology, 2074 Roswell Park Comprehensive Cancer Center, Buffalo, New York 14263, United States

## Abstract

Three pentadentate ligands based on 1,4,7-triazacyclononane (TACN) bearing 6-methyl-2-picolyl pendants and ancillary groups alkyl-sulfonate (L1), benzyl (L2), or proton (L3) were prepared to generate Fe­(II) and Co­(II) complexes as paramagnetic shift (parashift) agents with available coordination sites for anion binding. The solid-state structure of [Co­(L2)­(NO_3_)]­(NO_3_) confirms a six-coordinate complex cation with distorted octahedral geometry. ^17^O NMR spectroscopy studies support rapidly exchanging inner-sphere water for [Co­(L1)]^+^, [Co­(L2)]^2+^, and [Co­(L3)]^2+^ at neutral pH and for [Fe­(L1)]^+^ and [Fe­(L2)]^2+^ under mildly acidic conditions. The methyl proton resonances of [Co­(L1)]^+^ and [Co­(L2)]^2+^ appear at −45 ppm and +40 ppm and those of [Co­(L3)]^2+^ appear at −51 ppm and +21 ppm, compared to those of the symmetrical complex [Co­(L4)]^2+^ at 8 ppm. [Fe­(L1)]^+^ and [Fe­(L2)]^2+^ have methyl proton resonances at −38 or −39 ppm, which compare to that of [Fe­(L4)]^2+^ at 21 ppm. ^1^H and ^19^F NMR spectroscopy studies suggest fluoride binds as an inner-sphere ligand to Fe­(II) complexes and to [Co­(L1)]^+^ and [Co­(L2)]^2+^. Co­(II) and Fe­(II) complexes with bound water show second-sphere interactions with trifluorolactate and fluoride as supported by NMR studies. Magnetic resonance spectroscopy imaging at 7 T demonstrates strong, well-resolved methyl signals for [Co­(L1)]^+^ and [Co­(L2)]^2+^, highlighting their potential as parashift probes.

## Graphical abstract

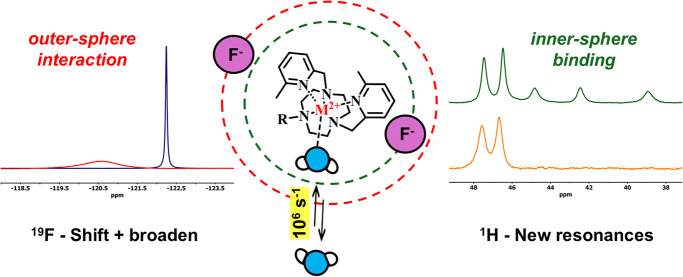

## Introduction

MRI contrast agents are successful diagnostic tools that enable the assessment of blood–brain barrier damage, detection of lesions, vasculature health, and organ perfusion.^1^ MRI contrast agents typically contain paramagnetic Gd­(III) centers incorporated into macrocyclic ligands to produce signal by modulating the relaxation rate of neighboring water protons. These contrast agents (or probes) are used to map physiological changes in function through dynamic contrast imaging, as they diffuse through blood and tissue.^2^ However, much of the current research on contrast agents focuses on the development of activatable probes that register changes in biochemical environments associated with disease states through either a targeted or responsive probe approach.^1,3^ It is proposed that such molecular imaging agents will improve diagnostic protocols.^3–8^ Notably, relaxivity-based MRI contrast agents such as those containing Gd­(III) may be challenging to use as responsive or activatable probes, as it is difficult to parse the signal contribution produced from activation of the probe from changes in the local probe concentration.^9^

An alternative to Gd­(III) complexes and other relaxivity probes is paramagnetic shift agents that produce distinct resonances for activated and inactivated probes.^10,11^ Paramagnetic shift agents may produce signal by chemical exchange saturation transfer (CEST) if there are protons on ligands that exchange with those of water,^11,12^ or by direct detection of the paramagnetically shifted ligand proton resonances (parashift).^13,14^ In either case, the paramagnetic center serves to shift the proton resonances far from those of water or tissue protons to minimize background signal interference. As responsive agents, parashift probes capitalize on the shift of their resonances upon activation to produce new sets of resonances.^14–16^ Such frequency-encoded changes allow for the detection of distinct signals from activated and nonactivated forms simultaneously^10^ and have been used to monitor temperature^14,17,18^ and pH.^15,19^

The metal ions in parashift probes have large magnetic susceptibility anisotropies that give rise to highly shifted resonances in neighboring nuclei.^20,21^ The difference between the chemical shift of the nucleus in a diamagnetic versus a paramagnetic environment, the hyperfine shift, is induced by a pseudocontact or dipolar contribution (δ^pcs^) and by a contact or through bond (δ^con^) contribution (eq 1). To date, paramagnetic lanthanide­(III) complexes have dominated the development of parashift probes.^10,14,17^ Lanthanide­(III) probes have been optimized by consideration of distance of the metal center to the protons and the incorporation of a large number of magnetically equivalent protons.^13,22^ This research led to Ln­(III) parashift probes that are detected in micromolar concentrations in mice. Paramagnetic d-block transition metal complexes have been more recently studied as parashift agents.^15,16,19,23–25^ Advantages to d-block complexes as probes include increased sustainability as earth abundant elements and potentially improved safety. Additional challenges to the development of d-block parashift probes as well as opportunities for responsive agents are based on the multiple accessible oxidation and spin states of the transition metal center.^26^ The most highly studied transition metal paramagnetic shift agents contain high-spin Fe­(II), low-spin Fe­(III), high-spin Co­(II), or Ni­(II).^15,16,27,28^ Unlike protons in paramagnetic Ln­(III) complexes, protons in paramagnetic d-block metal complexes may experience substantial contact contributions to the hyperfine shift,^20^ even for nuclei that are several bonds away from the metal center.^29^ It is thus challenging to predict the chemical shift of the protons in d-block parashift agents due to the dual effect of contact and pseudocontact contributions.

1 $$\delta^{\mathrm{obs}}{=\delta }^{\mathrm{dia}}{+\delta }^{\mathrm{con}}{+\delta }^{\mathrm{pcs}}$$

One application of interest to the researchers in the molecular imaging field is the development of metabolite-responsive probes.^8^ This application generally requires available coordination sites at the metal center to interact with the metabolites. For example, Ln­(III)-based parashift agents have been used to detect lactate, as an important metabolite with levels correlated to cancer.^30–33^ Lactate binds to available coordination sites of Ln­(III) complexes that contain a heptadentate macrocyclic ligand.^32,34^ For certain Ln­(III) complexes, the lactate is in rapid exchange with the paramagnetic center, so that the presence of lactate is detected through a chemical shift change. This has been used to distinguish intracellular and extracellular lactate.^31^ Alternatively, strongly bound lactate gives rise to new sets of paramagnetically shifted proton resonances.^35^ In these complexes, the OH of the lanthanide-bound lactate can be imaged as an exchangeable proton in the z-spectrum through CEST.^34^ Lactate also binds to Ln­(III) complexes containing hydroxypropyl pendant groups to shift the CEST peak of the OH group upon binding to metabolites.^35^

There are few examples of metabolite-responsive parashift agents based on d-block elements.^16^ Most transition-metal parashift agents contain divalent metal ion centers bound to neutral donor groups such as heterocycles, amides, or hydroxyl groups.^26,28^ Moreover, most d-block shift agents reported to date are coordinatively saturated and lack a site for bound water,^16,24,26^ with a few exceptions for paraCEST^36,37^ or parashift agents.^38^ The rationale for studying coordinatively saturated complexes is to maintain the high symmetry of the probe along with a large number of magnetically equivalent protons in addition to simplifying the solution chemistry and increasing kinetic inertness. For example, Fe­(II) complexes with an inner-sphere water may form hydroxides and oxidize to Fe­(III).^36^ However, metabolite sensing generally requires an open coordination site for binding to the analytes. For example, a Co­(II) complex with a tetradentate ligand was shown to act as a sensor for lactate, fluoride, and citrate in a complex that likely has two available coordination sites.^38^ In addition to ^1^H-based parashift agents, ^19^F-based shift agents are becoming common and many of these contain early transition-metal ions.^39,40^ Such agents modulate the chemical shift of the ^19^F resonance and its relaxation times to enable the distinction between activated and nonactivated forms of the probe.^41,42^

Here we present three new pentadentate ligands (L1, L2, and L3) along with their Co­(II) complexes and Fe­(II) complexes as responsive parashift agents. These complexes are compared to analogous Co­(II) and Fe­(II) complexes of L4, which have been studied as some of the first transition-metal parashift agents.^19,25^ Both [Co­(L4)]^2+^ and [Fe­(L4)]^2+^ are stable as high-spin divalent complexes and are very water-soluble, but neither of the complexes has methyl proton resonances that are sufficiently shifted for further development as parashift agents. In contrast, analogous Co­(II) or Fe­(II) complexes of L1, L2, and L3 have highly shifted methyl resonances (>40 ppm) that are suitable for parashift studies and a coordination site for water to facilitate interactions with anions and metabolites for sensing. The two macrocycles differ by the ancillary group and contain either a benzyl group as a noncoordinating pendant or an alkyl sulfonate group to impart a negative charge as a potentially useful feature for future in vivo studies^43^ or a proton. The interaction of the complexes with lactate, trifluorolactate, pyruvate, malate, and fluoride is examined. This study highlights the challenges of developing open coordination sphere Co­(II) probes and their Fe­(II) analogues and shows that a simple change in coordination may produce large changes in the chemical shift of reporter methyl proton resonances. The methyl resonances are sufficiently shifted from the water proton resonances for MRS phantom imaging at 7 T with successful exclusion of background HOD signal.

## Results and Discussion

### Synthesis of Ligands and Complexes

The macrocyclic ligands were prepared by direct alkylation of protected 1,4,7-triazacyclononane (TACN) with 1,3-propane sultone for L1 or benzyl bromide for L2 (as shown in Schemes S1 and S3), followed by isolation of the cationic product, and deprotection analogous to similar procedures.^44^ Addition of the coordinating pendants was accomplished by using direct alkylation of the TACN derivative by 2-bromomethyl-6-methylpyridine for L1. Direct alkylation was preferred in the preparation of L1 due to the necessity of using polar solvents, such as ethanol, to dissolve the macrocyclic ligand with the sulfonate group (Scheme S1). Alternatively, reductive amination by using 2-carboxyl-6-methylpyridine in the presence of sodium triacetoxyborohydride (STAB) in dichloroethane with an acetic acid catalyst was the method of choice to prepare L2 (Scheme S3).^19,24,44^ L3 and L4 were synthesized by alkylation of TACN with 3-equiv of bromomethylpyridine. These two macrocycles were separated by column chromatography and used to further study how the arrangement of the pendent group changes the interactions with metabolites and fluoride as discussed below (Scheme S5).

Co­(II) and Fe­(II) complexes of L1, L2, and the Co­(II) complex of L3 were prepared to study the effect of the different ancillary groups and for comparison with Co­(II) and Fe­(II) complexes of L4 that were reported previously (Schemes S7–S8).^19^ The Co­(II) complexes were prepared by the addition of Co­(NO_3_)_2_ or Co­(ClO_4_)_2_ to their respective ligands in methanol (Schemes S2, S4, S6). The iron complexes were prepared by the addition of Fe­(CF_3_SO_3_)_2_ in methanol to L1 (Scheme S2) or L2 as reported.^44^ Characterization of the complexes in solution by using the Evans method gave μ_eff_ values for [Fe­(L1)]^+^ and [Fe­(L2)]^2+^ consistent with high-spin Fe­(II) complexes (Table 1). Values for [Co­(L1)]^+^, [Co­(L2)]^2+^, or [Co­(L3)]^2+^ (Figures S1–S4, Table 1) were consistent with high-spin Co­(II).

**Table 1:** Effective Magnetic Moments, *R*_1_ and *R*_2_ Rate Constants of Methyl Protons, Water Exchange Rate Constants, and Temperature Coefficients

				Methyl proton			
Complex	μ_eff_	LOD (μM)	Chemical shift (ppm)	*R*_1_ (s^–1^) 11.7 T	*R*_2_ (s^–1^) 11.7 T	CT (ppm/°C)	*k*_ex298 K_ (s^–1^) × 10^6^
[Fe(L1)]^+^	4.6	2000	16, −38	-	1000	-	7.8
[Fe(L2)]^2+^	5.3	50	29, −39	-	850	-	2.4
[Co(L1)]^+^	4.3	125	40, −45	460, 370	710, 670	0.25	6.1
[Co(L2)]^2+^	4.8	62.5	40, −47	460, 400	720, 590	0.25	3.1
[Co(L3)]^2+^	4.6	130	22, −51	-	1300, 1200	0.26	5.9
[Fe(L4)]^2+^ ^a^	5.7	25	21	420 (356, 9.4T)^b^	650 (600, 9.4T)	0.11	-
[Co(L4)]^2+^ ^a^	5.8	8	8.1	180 (140, 9.4T)^b^	210 (200, 9.4T)	0.02	-

a From ref. 19.

b From ref. 25.

### Crystal Structure of [Co­(L2)­(NO_3_)]­(NO_3_) Complex

X-ray crystallography studies showed a hexacoordinate [Co­(L2)­(NO_3_)] complex cation with three bound nitrogens of the TACN macrocycle, two 6-methyl-2-picolyl nitrogens and one nitrate oxygen donor (Figure 1). The [Co­(L2)­(NO_3_)]­(NO_3_) complex crystallizes in the monoclinic crystal system and the centrosymmetric space group P2_1_/n. The twist angle (θ) of the complex was determined as the dihedral angle formed between the centroids of two mean planes and two atoms, each selected from one of the planes. One plane was defined by the two coordinated pyridyl nitrogen atoms and the bound oxygen of the nitrate, while the other was defined by the three coordinated nitrogen atoms of the macrocycle. Based on the twist angle (−49.4°) and the direction (counterclockwise) of rotation of the mean planes, the overall chirality of the complex is determined as Λ and based on the helicity of the macrocyclic backbone the chirality is determined as λλλ. The asymmetric unit of [Co­(L2)­(NO_3_)]­(NO_3_) contains one full isomer Λ­(λλλ) (Figure 1) of this complex, and as the space group is centrosymmetric, the crystal structure also contains the inversion isomer Δ­(δδδ). Additionally, the twist angle supports the distorted octahedral geometry of the complex. In comparison, the twist angle of the analogous iron complex, [Fe­(L2)­(CF_3_SO_3_)]­(CF_3_SO_3_), was 35.7° with clockwise rotation of the mean planes and to give one full isomer Δ­(λλλ) and an inversion isomer Λ­(δδδ). In comparison with [Co­(L4)]^2+^, [Co­(L2)]^2+^ has a 6-methyl-2-picolyl replaced by a nitrate ion. This replacement produces a slight change in the bond angle between the nonmacrocyclic nitrogen donors of pyridine (104°) compared to that of the symmetric system of [Co­(L4)]^2+^ (103°). The Co–N bond lengths of the TACN nitrogen donors and pyridyl nitrogen donors are similar to those of the [Co­(L4)]^2+^.^19^

**Figure 1:**
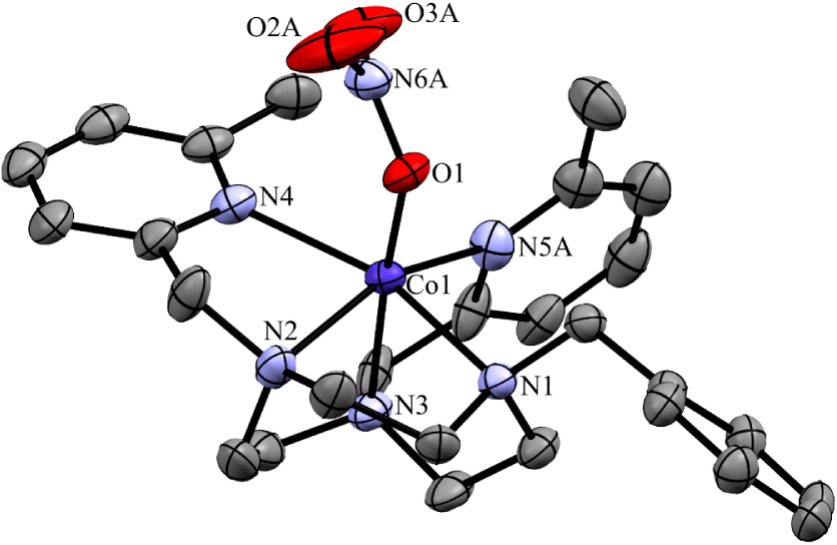
Molecular structure of [Co­(L2)­(NO_3_)]^+^ cation—Λ­(λλλ) as an ORTEP diagram (hydrogen, counteranion, solvent molecule, and occupancy disorder omitted for clarity).

### Solution Characterization

The aqueous solution chemistry of the Fe­(II) and Co­(II) complexes was characterized toward their development as parashift probes. All Co­(II) complexes were highly soluble in buffered aqueous solutions to at least 10 mM over the pH range of approximately 2–10. [Fe­(L2)]^2+^ was soluble at neutral pH up to 5 mM and up to 10 mM at pH 5. A study of the kinetic inertness of the complexes by monitoring electronic absorbance bands (400–700 nm) showed that the Co­(II) complexes were inert in 20 mM HEPES over a period of 4 h at pH 7.4 or in the presence of 1 equiv of Zn­(II) (Figures S5–S10). Analogous UV–vis spectroscopy studies on [Fe­(L2)]^2+^ supported inertness to dissociation under similar conditions.^44^ [Fe­(L1)]^+^ was soluble at 10 mM in solutions at acidic pH 4 but precipitated to give a red solid at pH 6 or higher, which is why absorbance for [Fe­(L1)]^+^ was only recorded at acidic pH (Figure S11). However, the addition of 1 equiv of lactate, malate, trifluorolactate, pyruvate, or fluoride increased the solubility of [Fe­(L1)]^+^ to 10 mM at neutral pH. None of the Co­(II) complexes or the Fe­(II) complexes^44^ were prone to oxidation under any of the conditions studied here.

The solution chemistry of the Co­(II) and Fe­(II) complexes was further studied by NMR spectroscopy. In these studies, ^1^H and ^19^F NMR spectra were collected mostly in D_2_O for convenience with a few studies in H_2_O for comparison, whereas ^17^O NMR studies were done in H_2_O only. Given that a change from H_2_O to D_2_O may subtly affect interactions with anions of the complexes with the solvent, a few studies of anion binding were examined in both D_2_O and H_2_O. It is well known that the properties of inorganic complexes differ in D_2_O versus H_2_O as the pH/pD approaches the p*K*_a_ values of the complexes.^45^ However, the ionization values of the complexes studied here lie outside the pH/pD values used in the titrations,^44^ so large differences are not expected.

The ^1^H NMR spectra of [Fe­(L1)]^+^, [Co­(L1)]^+^, [Fe­(L2)]^2+^, [Co­(L2)]^2+^, and [Co­(L3)]^2+^ show 28, 25, 24, 23, and 20 hyperfine-shifted proton resonances (Figures 2, S15), respectively, which are consistent with a single diastereomeric form in solution. The TACN ethylene groups may adopt one of two configurations (δδδ or λλλ) and the methylpyridine pendants also have two configurations based on their directional orientation.^19^ The two distinct methyl resonances were tentatively assigned at −45 ppm and +40 ppm for [Co­(L1)]^+^, −47 ppm and +40 ppm for [Co­(L2)]^2+^ (Figure 2C–D), and −51 ppm and +22 ppm for [Co­(L3)]^2+^ (Figure S15), based on their integration and relatively short *T*_1_ relaxation times. (These relaxation times are expressed as *R*_1_ rate constants (*R*_1_ = 1/*T*_1_) as shown in Table 1. Data not shown for [Co­(L3)]^2+^) (Tables 1, S5–S6). Similar to [Co­(L4)]^2+^,^19,25^ the relaxation times of the methyl resonances of [Co­(L1)]^+^ and [Co­(L2)]^2+^ are anticipated to be shorter than those of the pyridine protons due to their close proximity to the paramagnetic center. Resonances at +2.4 ppm, −8.6 ppm, and −51 ppm for [Co­(L1)]^+^ were attributed to the alkyl chain of the sulfonate pendant. The ^1^H NMR spectra of [Fe­(L1)]^+^ and [Fe­(L2)]^2+^ show 28 and 26 shifted proton resonances (Figure 2A–B), respectively, consistent with a single diastereomer. One of the two methyl group resonances was assigned at −38 ppm for [Fe­(L1)]^+^ and −39 ppm for [Fe­(L2)]^2+^. The second methyl group proton resonances were not readily assigned due to overlap with other proton resonances. The methyl proton resonances for the Fe­(II) complexes were assigned based on their integration and relatively short *T*_2_ relaxation times, expressed as rate constants (*R*_2_ = 1/*T*_2_) in Table 1.^42^ Detailed versions of the spectra are provided in Figures S12–S15 and experimental methods are described in the SI. Water proton relaxation is not reported here given that Fe­(II) and Co­(II) complexes have low reported relaxivities^44,46,47^ due to their rapid electronic relaxation rates.^21^

**Figure 2:**
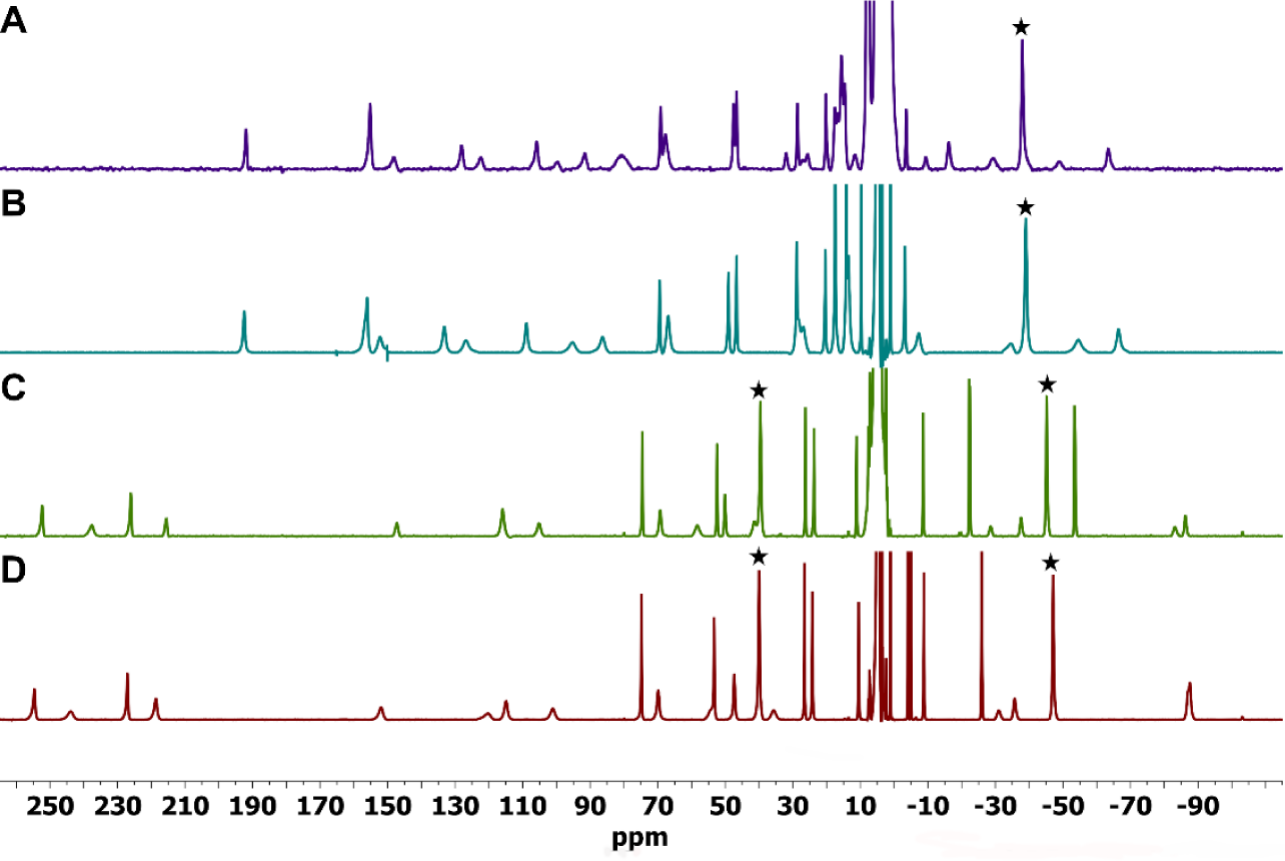
^1^H NMR spectra of (A) [Fe­(L1)]^+^ (pD 4.4), (B) [Fe­(L2)]^2+^ (pD 3.9), (C) [Co­(L1)]^+^, and (D) [Co­(L2)]^2+^ (pD 7.8), in D_2_O. Methyl proton resonance assignments are marked by asterisks.

To evaluate the most promising Co­(II) complexes as parashift agents, *R*_1_ and *R*_2_ relaxation rate constants were measured for the methyl proton resonances and compared to those previously measured for the [Co­(L4)]^2+^ complex. *R*_1_ was measured by inversion recovery experiments, whereas *R*_2_ was estimated by recording spectral line widths as described in the experimental section. Notably, the *R*_1_ values for the methyl resonances of [Co­(L1)]^+^ and [Co­(L2)]^2+^ of 460 to 370 s^–1^ at 11.7 T are about 2-fold larger than those of [Co­(L4)]^2+^ which was measured at a slightly lower field strength (9.4 T), but still within the range of values that are recommended for parashift agents (<400 s^–1^).^25^ Here the *R*_1_ rate constant should be sufficiently large to allow for rapid imaging pulses to increase the signal-to-noise ratio. Nominally, *R*_1_/*R*_2_ ratios should be as close to unity as possible but greater than 0.5. Both [Co­(L1)]^+^ and [Co­(L2)]^2+^ have *R*_1_/*R*_2_ values that are within range (0.6), albeit slightly less favorable than that of [Co­(L4)]^2+^ at 0.7. The *R*_2_ rate constant for [Co­(L3)]^2+^ was about 2-fold larger than that of the other two Co­(II) complexes, which we attribute to the greater fluxionality of this complex. The NMR properties of the Fe­(II) complexes were not further studied for parashift applications as they were less highly shifted than those of the Co­(II) complexes.

### Variable Temperature ^1^H NMR Spectroscopy Studies

Proton NMR spectra of the Co­(II) complexes were recorded over the temperature range of 25 to 75 °C to study the temperature dependence of the proton chemical shifts as well as to monitor dynamic processes. As shown in Figures S16–S21, the proton resonances of [Co­(L1)]^+^ and [Co­(L2)]^+2^ remain relatively sharp up to about 60 °C; thereafter, broadening of the resonances becomes significant. The resonances assigned to the methyl groups remain relatively narrow even above 60 °C, but most of the other resonances broaden severely at the higher temperatures, signifying a dynamic process that likely involves interconversion between stereoisomers. For [Co­(L3)]^2+^, broadening of the proton resonances becomes notable at lower temperatures (50 °C). This suggests that the absence of an ancillary group at the third TACN nitrogen gives a more dynamic Co­(II) complex. In comparison, the symmetrically substituted Co­(II) complex, [Co­(L4)]^2+^, maintained sharp resonances at much higher temperatures,^19^ suggesting that the greater rigidity of this symmetrical system is advantageous in the development of temperature-dependent parashift probes.

Parashift probes have been studied as temperature-responsive agents toward applications in thermometry.^10,16,19,48^ In these applications, the chemical shift change as a function of temperature for each proton resonance is expressed as a temperature-dependent coefficient (CT) in ppm/°C, with larger values associated with more temperature-sensitive probes. The temperature dependence of the hyperfine-shifted protons is determined by a Fermi contact term (T^–1^) and two pseudocontact terms (T^–1^ and T^–2^).^49,50^ However, the chemical shifts of the proton resonances in these Co­(II) complexes are expected to vary linearly with temperature over the small temperature ranges studied here.^19^ The temperature coefficients (CT) of the most intense proton resonances of the Co­(II) complexes are moderately large. The resonances assigned to the methyl groups of [Co­(L1)]^+^, [Co­(L2)]^2+^, and [Co­(L3)]^2+^ have CT values of 0.25–0.26 ppm/°C (Tables S7–S9). In comparison, the reporter methyl group of [Co­(L4)]^2+^ has a CT of only 0.017 ppm/°C although some of the more highly shifted but less intense proton resonances have CT values of up to 0.48 ppm/°C.^19^

### Limit of Detection Studies

The limit of detection (LOD) is the lowest concentration of probe at which proton resonances can be observed with a signal-to-noise ratio (SNR) of at least 4. LOD measurements were carried out at a chosen set of conditions to compare the Co­(II) and Fe­(II) complexes prepared here with the closed coordination complexes prepared earlier. The limit of detection is a function of the intensity of the proton resonance, which is related to the number of equivalent protons, and to the spectral line width of the resonances (related to *R*_2_). The spectral line width of the resonances is modulated by R_2_ relaxation effects as well as by dynamic processes. The LOD is also influenced by the longitudinal relaxation rate of the protons (*R*_1_) and the experimental pulse repetition rate. We chose to study solutions of the complexes at 25 °C at neutral pD with NMR spectra collected at 500 MHz over 5 min with delay time of 0.1 s to compare the Co­(II) and Fe­(II) complexes presented here. Under these conditions, the LOD for [Co­(L1)]^+^ is 125 μM with a signal-to-noise ratio (SNR) of 5.4 for the methyl proton at −45 ppm, while for [Co­(L2)]^2+^ the LOD is 63 μM with an SNR of 6.7 for the methyl proton at −47 ppm. [Co­(L3)]^2+^ has a LOD of 130 μM with an SNR of 7.2 for the methyl proton at −51 ppm. For [Fe­(L2)]^2+^ the LOD was 50 μM with an SNR of 6.8 for the methyl proton at −39 ppm (Figures S22–S26).

In comparison, the symmetrical L4 complexes showed improved LODs, with [Co­(L4)]^2+^ reaching as low as 8 μM, while [Fe­(L4)]^2+^ was 25 μM (Figures S27–S28). Comparison with the asymmetrical complexes shows that [Co­(L4)]^2+^ has an 8-fold improved LOD, more than the 3-fold factor expected from the number of equivalent protons. This difference is attributed to the larger line widths of the asymmetric Co­(II) complexes in comparison to the symmetrical complex, which shows very narrow resonances. However, [Fe­(L4)]^2+^ shows only a 2-fold LOD advantage over [Fe­(L2)]^2+^.

### ^17^O NMR and Water Exchange Rates

To verify that the complexes have an inner-sphere water that is exchangeable on the NMR time scale, variable temperature ^17^O NMR studies were carried out. A plot of ln­(1/*T*_2r_) versus temperature is shown in Figure 3 and additional studies are shown in Figures S29–S33. Fitting the data to Swift–Connick plots indicates *q* = 1 with water exchange rate constants of 10^6^ to 10^7^ s^–1^ (Tables S10–S17). Interestingly, [Co­(L1)]^+^, [Co­(L2)]^2+^, and [Co­(L3)]^2+^ show slightly different ^17^O line broadening and exchange rates in these experiments, suggesting subtle differences in water interactions due to ancillary groups (Figures 3A, S29, S31–S32). [Co­(L1)]^+^ produces the most highly shifted and broadened ^17^O NMR resonance, perhaps due to the sulfonate group that may serve to modulate the water interaction and exchange. The *k*_ex_ values range from 3.1 × 10^6^ to 6.1 × 10^6^ s^–1^. (Tables S10–S15). Similarly, variable temperature ^17^O NMR studies of [Fe­(L1)]^+^ were consistent with a value of *q* = 1 at pH 4 with *k*_ex_ = 7.8 × 10^6^ s^–1^ (Tables S16–S17, Figures 3B, S33A). The inner-sphere water exchange rate constant of [Fe­(L2)]^2+^ was reported as 2.4 × 10^6^ s^–1^ at pH 5.^44^

**Figure 3:**
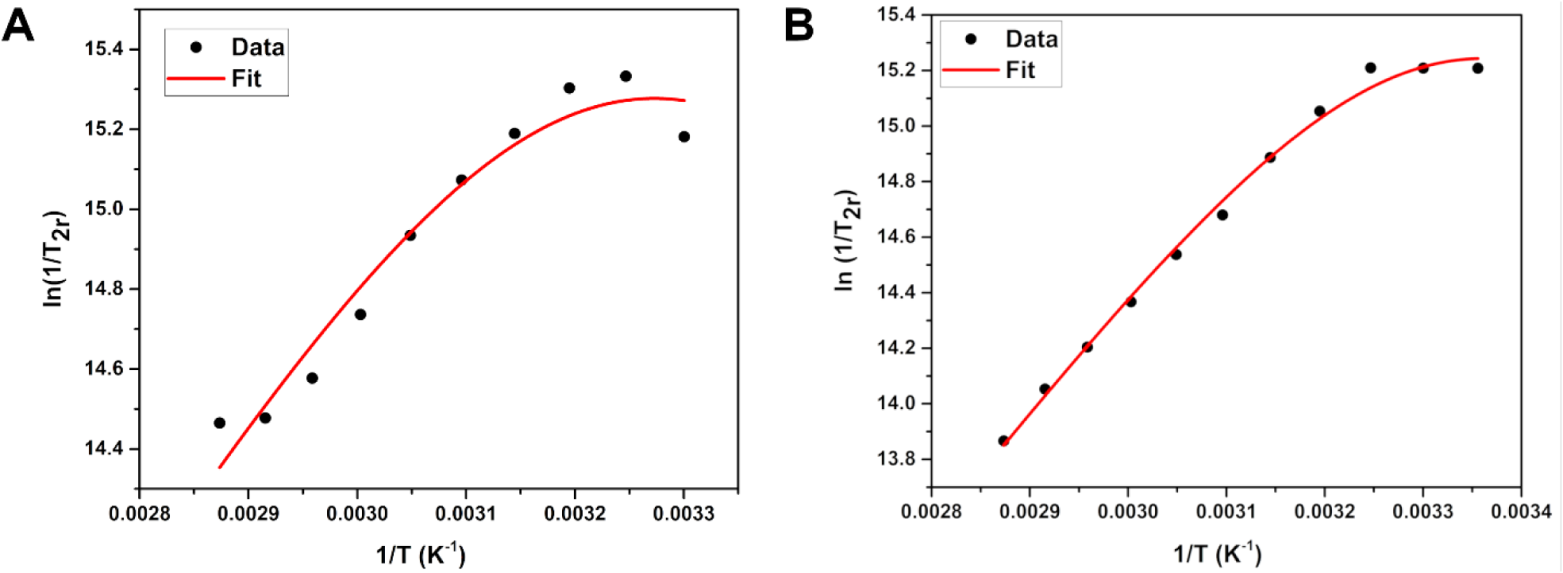
Transverse ^17^O relaxivity, ln­(1/*T*_2r_), as a function of temperature for (A) [Co­(L1)]^+^ in water at pH 7.4 and (B) [Fe­(L1)]^+^ in water at pH 4. The solid line represents a fit to the data using the Swift–Connick equations. Conditions: 10 mM complex, 0.1 M NaCl, 1% (v/v) H_2_^17^O.

The addition of 1 equiv of lactate, pyruvate, malate, or fluoride to [Co­(L1)]^+^ does not markedly affect the rate constant for water ligand exchange in the Co­(II) complex as shown by variable temperature ^17^O NMR spectra. These data are consistent with the absence of inner-sphere interactions of the anions with [Co­(L1)]^+^ to disrupt water exchange significantly (Figure S30 and Tables S10–S11). In contrast, variable temperature NMR studies were consistent with the absence of water ligand exchange on the NMR time scale for [Fe­(L1)]^+^ at neutral pH in the presence of 1 equiv (or excess) of pyruvate or lactate (Tables S16–S17, Figure S33B–D). This result may be attributed to several potential factors. First, interaction of the metabolite with the [Fe­(L1)]^+^ complex may modulate water exchange, although the metabolite is clearly not bound as an inner-sphere ligand as shown by further NMR studies described below. Alternatively, changes may be related to pH as studies showing an exchangeable inner-sphere water on [Fe­(L1)]^+^ were done at acidic pH to maintain solubility, whereas those with the metabolite were at neutral pH where formation of a hydroxide ligand is more likely. We favor a role for the sulfonate or anionic metabolite in modulating the water exchange, however, considering that the water ligand of the related complex, [Fe­(L2)]^2+^, deprotonates only at high pH (p*K*_a_ = 9.8).^44^

### Titrations with Metabolites, Fluoride, and Trifluorolactate

The addition of 1 equiv of lactate, trifluorolactate, malate, or pyruvate to solutions containing 10 mM [Co­(L1)]^+^, [Co­(L2)]^2+^, or [Co­(L3)]^2+^ in D_2_O at pH 7.4 and 25 °C produced negligible changes in the proton NMR spectrum. These data suggest that the metabolites do not bind as inner-sphere ligands under these conditions (Figures S34–S37, S39–S43) given that the replacement of inner-sphere water with any of these anions would be expected to produce new proton resonances for a distinct complex. As expected, the addition of 1 equiv of trifluorolactate to [Co­(L4)]^2+^, as an example of a complex lacking an available coordination site, showed essentially no change in the ^1^H NMR spectrum (Figure S46).

Six-coordinate macrocyclic complexes of Fe­(II) with an open coordination site may have complicated aqueous solution chemistry and it is more challenging to study anion binding. For example, [Fe­(L2)]^2+^ binds to HEPES buffer or other oxyanions.^44^ In other cases, deprotonation of Fe­(II) inner-sphere water to give a hydroxide ligand favors oxidation to Fe­(III).^36^ The [Fe­(L1)]^+^ complex studied here is insoluble at neutral pH at micromolar concentrations as opposed to [Fe­(L2)]^2+^, which exhibits up to 5 mM aqueous solubility at pH 7. Interestingly, the addition of α-substituted carboxylic acids such as lactate, malate, or pyruvate to [Fe­(L1)]^+^ dramatically increased the solubility of the complex at neutral pH to at least 10 mM. In combination with the lack of an exchangeable water as shown by variable temperature ^17^O NMR spectroscopy at pH 7, these data suggest interaction of the metabolite with the Fe­(II) complex. Second-sphere interactions are most likely, as the ^1^H NMR resonances of the Fe­(II) complex remain unchanged upon the addition of 1 equiv of lactate or pyruvate to [Fe­(L1)]^+^ at acidic pD. Subsequent adjustment to pD 7.8 yields spectra that closely resemble those of the free complex at pD 4.4 (Figures S47–S48). Similarly, the addition of 1 equiv of trifluorolactate to [Fe­(L2)]^2+^ yields spectra that resemble the free complex, consistent with a lack of inner-sphere interactions between the anion and the complex (Figure S49).

In contrast, the addition of an equivalent of potassium fluoride (KF) to [Co­(L1)]^+^ or to [Co­(L2)]^2+^ complexes in D_2_O produced new hyperfine shifted ^1^H resonances, consistent with the formation of new complexes, albeit in minor amounts (Figure 4C–D). These are most likely complexes with an inner-sphere fluoride given that the replacement of inner-sphere water with fluoride would be expected to modulate the hyperfine shifts of all the other ligand protons. The addition of potassium fluoride to [Fe­(L1)]^+^ or [Fe­(L2)]^2+^ also generated additional proton resonances signifying the formation of new complexes (Figure 4A–B). The new resonances increased in intensity as additional fluoride was added (Figure S50). That this new complex is a fluoride complex is supported by mass spectrometry studies showing the presence of the [Fe­(L2)­(F)]^+^ complex cation (Figure S51). Calculations showed that the binding constants of fluoride to [Fe­(L1)]^+^ or to [Fe­(L2)]^2+^ (*K*_d_ of 13 and 17 mM, respectively) were an order of magnitude tighter than those of either [Co­(L1)]^+^ or [Co­(L2)]^2+^ complexes (250 and 180 mM) as shown in Table S18. In H_2_O, the *K*_d_ for the binding of fluoride to [Fe­(L2)]^2+^ was studied and found to be similar to that in D_2_O (16 mM, Table S18). No new resonances were observed upon the addition of an equivalent of KF to solutions of [Co­(L3)]^2+^ or to [Fe­(L4)]^2+^ as shown in Figures S42, S44.

**Figure 4:**
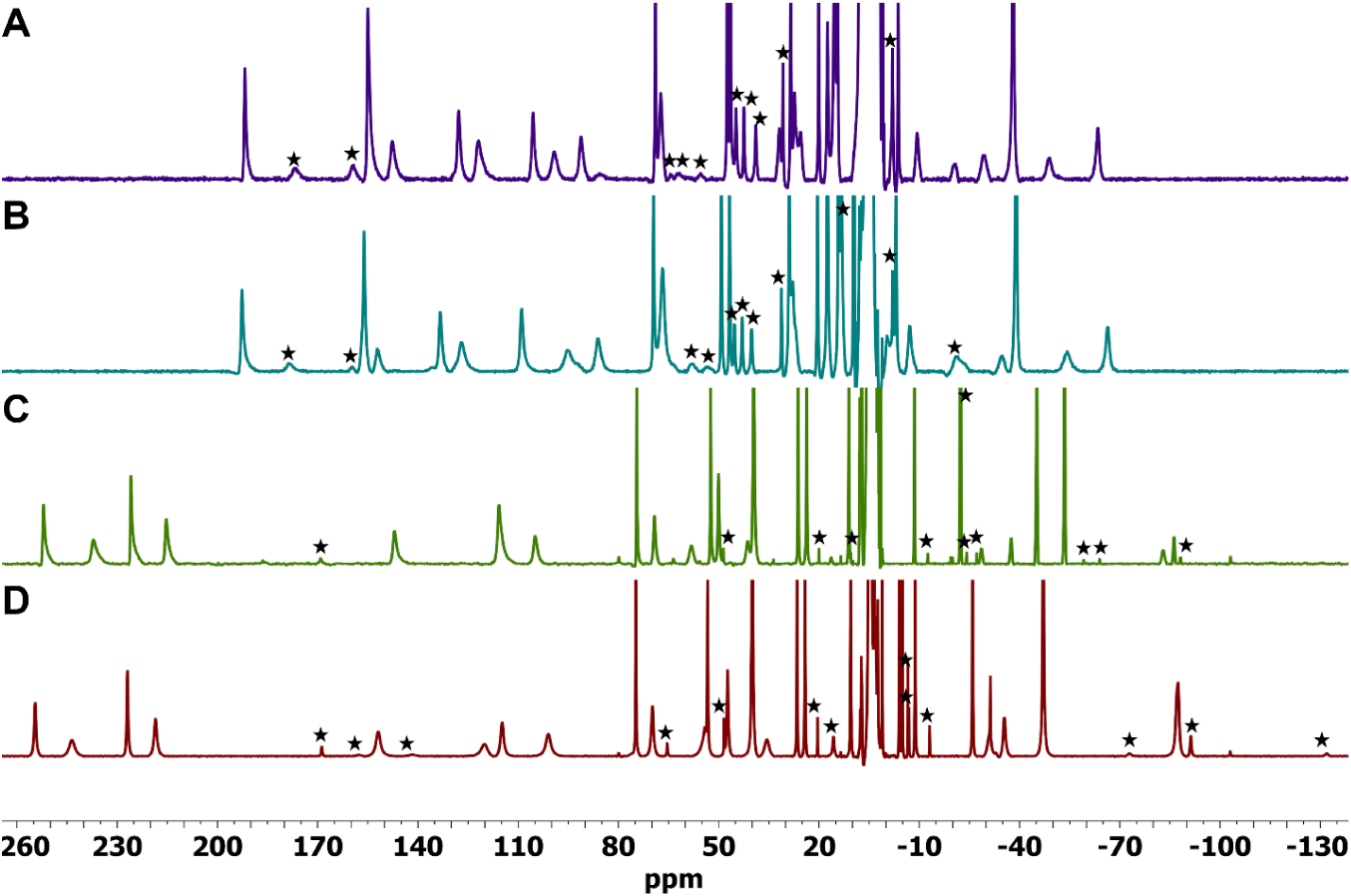
^1^H NMR spectra of the titration of KF with (A) [Fe­(L1)]^+^, (B) [Fe­(L2)]^2+^, (C) [Co­(L1)]^+^, and (D) [Co­(L2)]^2+^. Conditions: For (A), (C), and (D) Complex 10 mM, KF 10 mM, For (B) Complex 5 mM, KF 5 mM, pD 7.8, D_2_O. New proton resonances are marked by asterisks.

#### ^19^F NMR Studies of Second-Sphere Interactions

[Fe­(L2)]^2+^ and [Co­(L2)]^2+^ were titrated with KF under conditions of increasing complex or increasing fluoride as monitored by ^19^F NMR spectroscopy (Figures 5, S52–S62). In all cases, a single ^19^F resonance was observed, suggesting rapid exchange of fluoride species on the NMR time scale and a small change in chemical shift that is most consistent with second-sphere interactions. Experiments that maintain constant fluoride concentration while increasing the complex concentration show that [Co­(L2)]^2+^ shifts and broadens the fluoride resonance as the complex concentration increases (Figure 5B). The analogous experiment with [Fe­(L2)]^2+^ shows that the ^19^F resonance also shifts as more complex is added, although the single resonance is not quite as broad as that of the Co­(II) experiment (Figure 5A). The L1 analogs (Figures S54 and S56) and L3 (Figure S59) behave similarly in terms of broadening except for an additional upfield shift in the resonance of [Fe­(L1)]^+^ once the fluoride and complex are in equimolar concentrations (Figure S54). Under conditions of constant [Co­(L2)]^2+^ complex concentration and increasing fluoride (Figure 5D), the ^19^F resonance shifts slightly and broadens. In contrast (Figure 5C), the ^19^F resonance of fluoride in the presence of [Fe­(L2)]^2+^ shifts steadily as the fluoride concentration increases and broadening increases after two molar equivalents. The different appearances of the NMR titrations of fluoride with [Co­(L2)]^2+^ and [Fe­(L2)]^2+^ can be explained by the greater broadening of the ^19^F resonance in the presence of [Co­(L2)]^2+^ compared to that in the presence of [Fe­(L2)]^2+^. Differences in the rate of exchange of the second-sphere fluoride or in the difference between chemical shifts for bound and free fluoride would explain these results. Notably, there should also be minor amounts of inner-sphere fluoride complexes, [Fe­(L1)­F] and [Fe­(L2)­F]^+^, [Co­(L1)­F], and [Co­(L2)­F]^+^, under these conditions, which should give rise to new highly shifted ^19^F resonances in slow exchange on the NMR time scale as we observed in the ^1^H NMR spectroscopy experiments. However, resonances from inner-sphere fluoride complexes of Co­(II) or Fe­(II) are expected to be broadened as well as highly paramagnetically shifted. No additional resonances were detected in our experiments, even with long acquisition times and large sweep widths.

**Figure 5:**
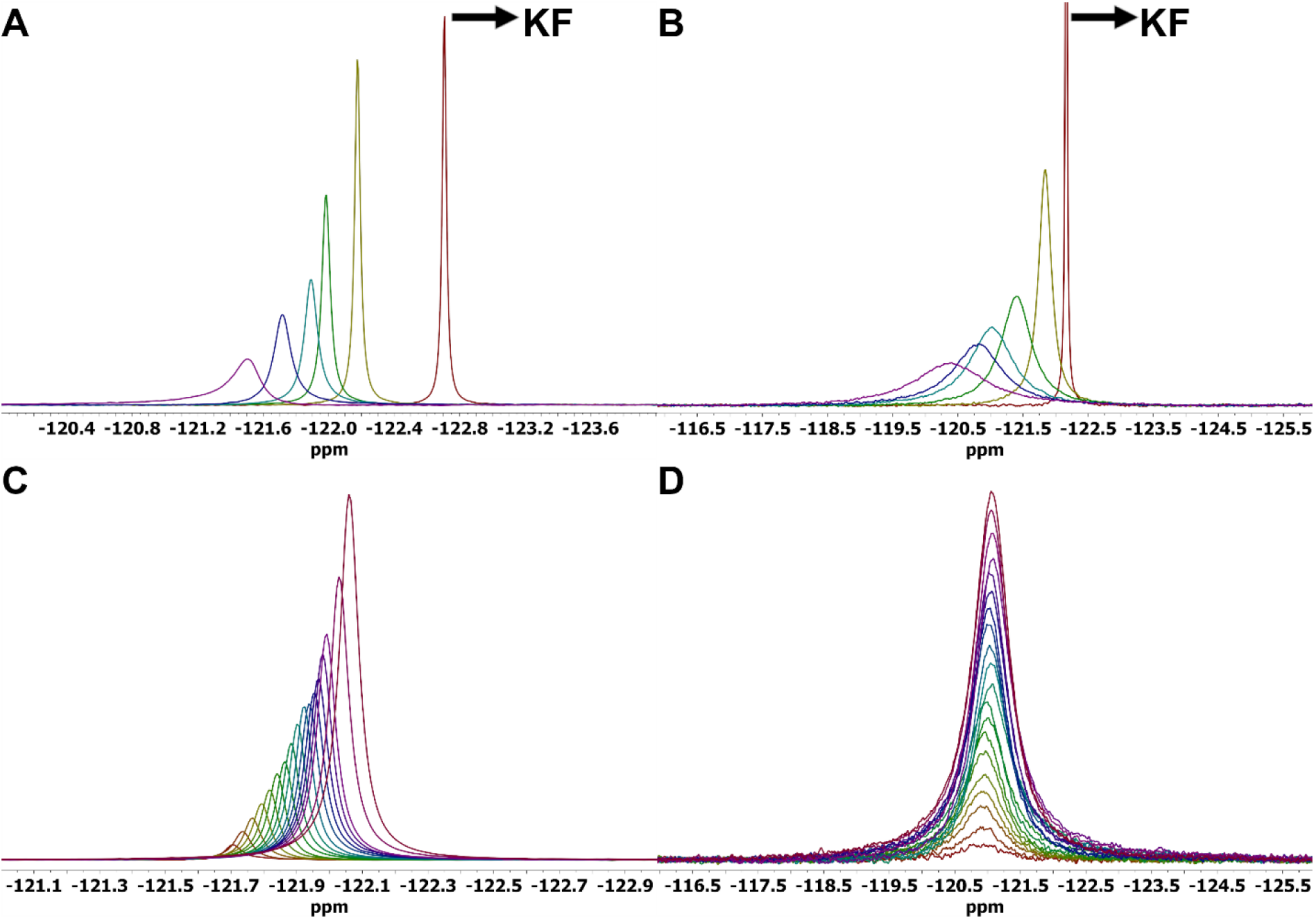
^19^F NMR titration of (A) 10 mM fluoride with 0–10 mM [FeL2]^2+^, (B) 10 mM fluoride with 0–10 mM [CoL2]^2+^, (C) 5 mM [FeL2]^2+^ with 1–25 mM fluoride, (D) 10 mM [CoL2]^2+^ with 1–20 mM fluoride. Conditions: pD 7.8, D_2_O, 0.1 M NaCl. The spectrum marked as KF was obtained in the absence of the metal complex.

The binding constant for fluoride to [Fe­(L2)]^2+^ (Table S18) was determined by fitting the plot of the change in ^19^F chemical shift versus the concentration of fluoride to a quadratic binding equation to give a *K*_d_ of 2.6 mM for what is presumably second-sphere fluoride binding (Figure S53). In comparison, the titrations studied by ^1^H NMR gave a larger *K*_d_ of 17 mM for the binding of the fluoride to give an inner-sphere complex with [Fe­(L2)]^2+^, as discussed above. Such an inner-sphere complex would presumably form from the displacement of water, and this displacement may be less favorable than the second-sphere fluoride interaction. [Co­(L3)]^2+^ titrations with KF showed similar results as those of [Co­(L2)]^2+^ and [Co­(L1)]^+^ (Figures S59 and S61). Given that there was only a small shift change, plots of the line width changes for second-sphere binding of KF with the Co­(II) complexes were examined. However, these did not give reliable binding constants due to severe broadening at high concentrations of the Co­(II) complex as well as the near linearity of the plot (Figures S57 and S60).

Titrations of Fe­(II) and Co­(II) complexes of L1 and L2 with trifluorolactate are shown in Figures 6, S63, S65–S67 as followed by ^19^F NMR spectroscopy. The addition of [Fe­(L2)]^2+^ to trifluorolactate results in a steady decrease in intensity and broadening of the trifluorolactate resonance (Figure 6B), whereas the addition of [Fe­(L1)]^+^ produces a decrease in intensity and increased broadening (Figure 6A). These differences are attributed to the sulfonate group that influences second-sphere interactions or may influence speciation in solution through aggregation or clustering of ions. On the other hand, the Co­(II) complexes broaden and shift the trifluorolactate resonance more than the Fe­(II) complexes, which we attribute to greater dynamic exchange broadening. The addition of [Co­(L2)]^2+^ (Figure 6D) produces a steady decrease in intensity similar to the Fe­(II) analog, and both [Co­(L1)]^+^ and [Co­(L3)]^2+^ (Figures 6, S68) produce broadening and a slight shift of the ^19^F NMR resonances. Titrations of [Co­(L1)]^+^ with trifluorolactate in H_2_O instead of D_2_O did not result in substantial changes (Figure S64) in the ^19^F NMR spectra. The titrations using excess trifluorolactate to the Fe­(II) or Co­(II) complexes were not very informative as the resonance broadened and shifted slightly for all complexes (Figures S63, S66–S67).

**Figure 6:**
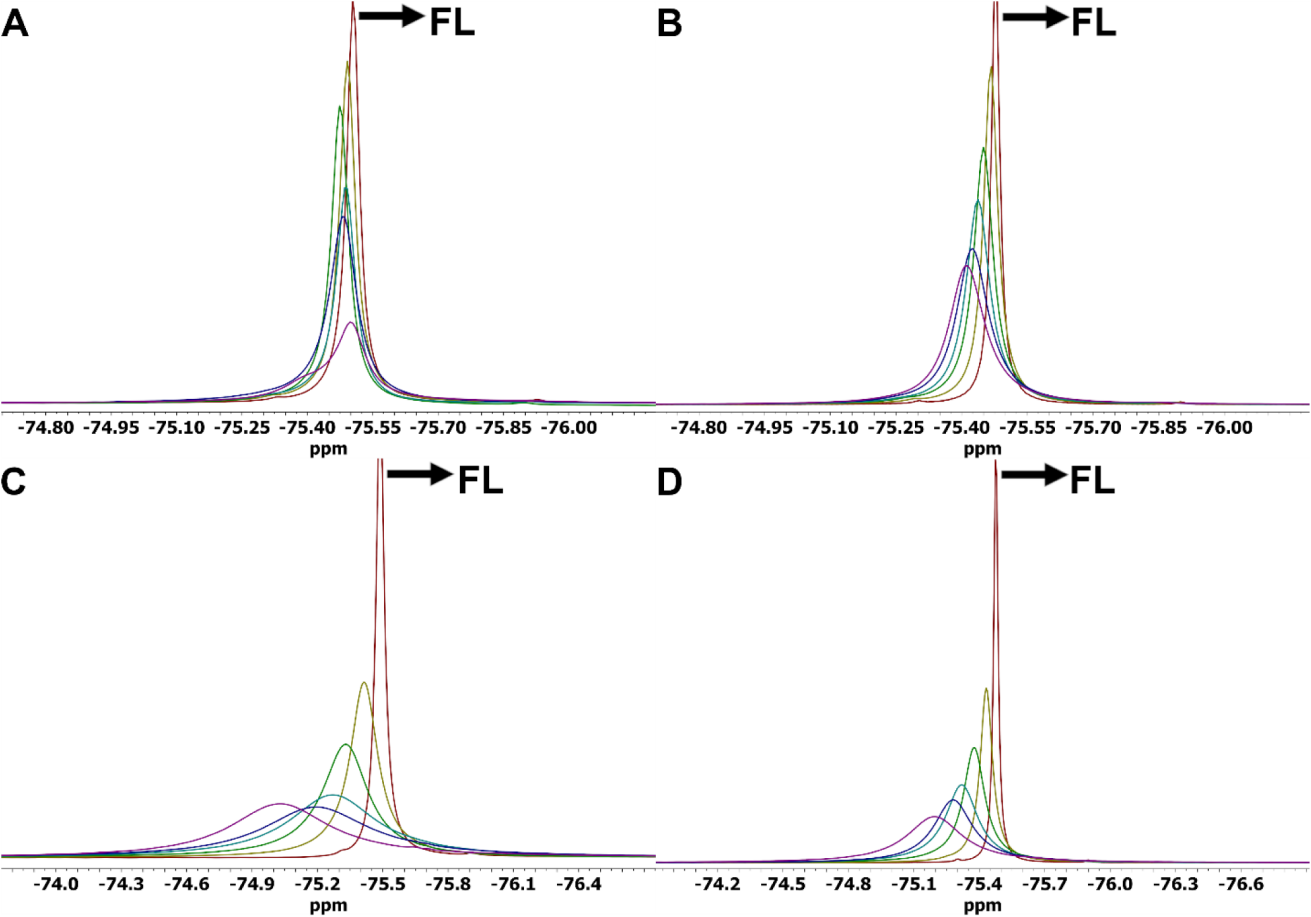
^19^F NMR titration of 10 mM trifluorolactate with (A) [FeL1]^+^, (B) [FeL2]^2+^, (C) [CoL1]^+^, and (D) [CoL2]^2+^. Conditions: complex 0–10 mM, pD 7.8, D_2_O, 0.1 M NaCl. The spectrum marked as FL was obtained in the absence of the metal complex.

In comparison, [Fe­(L4)]^2+^ and [Co­(L4)]^2+^ complexes did not broaden the ^19^F resonance in any of the titrations but instead simply shifted the resonance upon the addition of excess complex due to increasing bulk magnetic susceptibility contributions (Figures S70–S72). These data suggest that there is a difference in the nature of second-sphere complexes of trifluorolactate or fluoride with the Co­(II) and Fe­(II) complexes that have an inner-sphere water, compared to the [Fe­(L4)]^2+^ and [Co­(L4)]^2+^ complexes that lack such a site.

#### Phantom Imaging Studies

A 7 T MRI scanner was used for the phantom imaging of [Co­(L1)]^+^ and [Co­(L2)]^2+^. Acquisition parameters can be found in the Supporting Information. A sample containing ferumoxytol in HOD (on the left channel) was used as a reference to compare the image with focused short-range phantoms. Acquisition at 4.7 ppm gave the water peak and several additional proton resonances for [Co­(L1)]^+^, whereas excitation at −55 ppm gave only the two parashift resonances in this region. For [Co­(L2)]^2+^, centering the pulse at 4.7 ppm gave the water sample peak on the left and the water and an additional proton resonance for the paramagnetic shift sample on the right. Shifting the center of the pulse to −27 or 77 ppm gave additional parashift peaks in this region. These results show that the hyperfine-shifted proton resonance of the Co­(II) complexes could be imaged by changing the acquisition bandwidth and center frequency to obtain significant contrast that is unhindered by the water signal, as shown in Figure 7.

**Figure 7:**
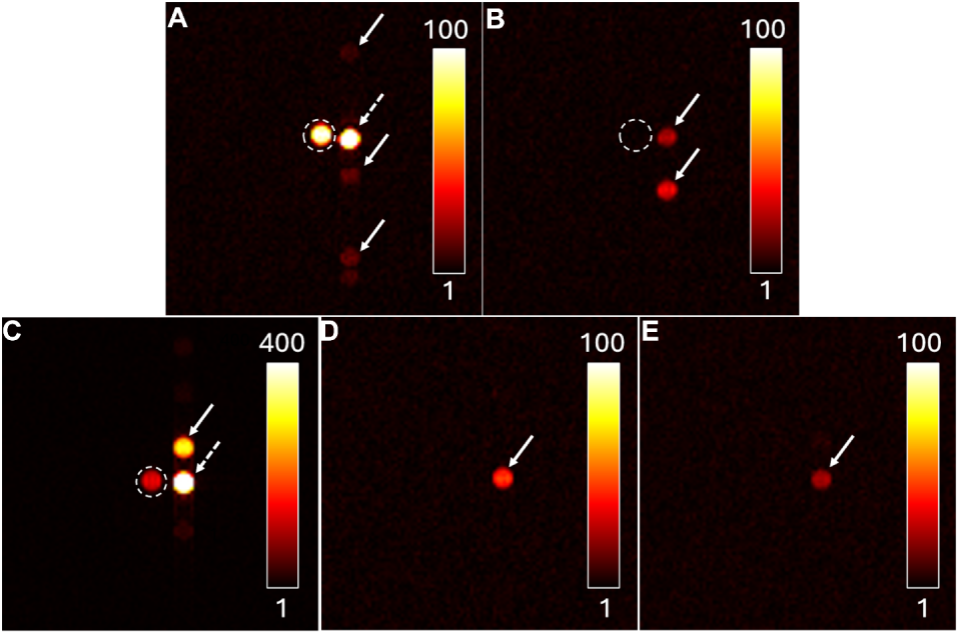
T_1_-weighted phantom imaging of Co­(II) complexes. Signal intensities represent signal-to-noise ratios. Acquisition at 4.7 ppm (A) for [Co­(L1)]^+^ shows a strong signal of the ferumoxytol phantom (dashed circle) and residual HOD arrow of the parashift sample (dashed arrow). Nearby Co­(II) proton resonances can be observed in the vertical (frequency-encoding) direction (solid arrows). Setting the excitation frequency to −55 ppm (B) results in the removal of the water signal (dashed circle) with two clear parashift resonances remaining. Acquisition at 4.7 ppm (C) for [Co­(L2)]^2+^ shows a strong signal of the ferumoxytol phantom (dashed circle) and residual HOD signal­(dashed arrow) of the parashift sample. A strong signal at 1.4 ppm is also present (solid arrow). Setting the excitation frequency at −27 ppm (D) and +77 ppm (E) reveals images of the parashift compound devoid of the residual HOD background. Intensity ranges are adjusted for improved visualization.

## Conclusions

The five Co­(II) or Fe­(II) complexes presented here have certain advantages as parashift agents in comparison to previously reported examples. Most notably, the methyl proton resonances are more highly shifted than those of the symmetrical complexes [Co­(L4)]^2+^ or [Fe­(L4)]^2+^,^19^ with only slightly larger spectral line widths than the symmetrical complexes. Notably, the methyl group proton resonances of [Co­(L1)]^+^ and [Co­(L2)]^2+^ are substantially sharper than the methyl resonances of a nonmacrocyclic Co­(II) complex studied as an anion-responsive paraCEST agent.^38^ The two distinct resonances for the Co­(II) complexes of L1 and L2 at approximately −45 and +40 ppm are in an appropriate range for magnetic resonance spectroscopy imaging as shown by the phantoms recorded for these resonances at 7 T. The Co­(II) complexes have narrow spectral line widths with a favorable *R*_1_ of 450 Hz and *R*_2_ of 700 Hz and an *R*_1_/*R*_2_ ratio of 0.6. MRS imaging studies showed that [Co­(L1)]^+^ and [Co­(L2)]^2+^ have methyl proton resonances that are detected by using broadband excitation. The assignment of proton resonances of the Fe­(II) complexes was less extensively studied because of solubility challenges and less highly shifted methyl proton resonances.

A disadvantage of inducing asymmetry in the complexes is the poorer limit of detection as there are two sets of three equivalent proton resonances from the methyl reporter groups. However, additional properties such as *R*_1_ and *R*_2_ of the reporter protons factor into the LOD. This leads to an 8-fold decrease in the LOD for [Co­(L1)]^+^ compared to [Co­(L4)]^2+^ but only a 2-fold decrease for [Fe­(L2)]^2+^ compared to [Fe­(L4)]^2+^ under our NMR spectrometer conditions. Moreover, the Co­(II) and Fe­(II) complexes with pentadentate ligands are more susceptible to dynamic processes at high temperatures, leading to some line broadening. However, the alkyl sulfonate and benzyl group ancillary pendants serve to increase the rigidity of the macrocyclic complex compared to the [Co­(L3)]^2+^ complex, which lacks such a group. [Co­(L3)]^2+^ is much more prone to dynamic processes as the temperature is increased from 25 to 60 °C. Surprisingly, the sulfonate group did not increase the solubility of the Co­(II) or Fe­(II) complexes. In fact, the [Fe­(L1)]^+^ complex had poorer solubility at neutral pH than did [Fe­(L2)]^2+^. That the Co­(II) complex did not suffer the same solubility decrease may be attributed to the more oxophilic Fe­(II) center forming less soluble species by interacting with the sulfonate pendant.

None of the metabolites, malate, pyruvate, or lactate, produced additional sets of proton resonances when added to the Co­(II) or Fe­(II) complexes that would signify the formation of an inner-sphere complex. Instead, data support second-sphere interactions of trifluorolactate with the complexes as shown by ^19^F NMR studies, based on the small chemical shift changes and broadening, and the fact that the ^1^H NMR spectra were unchanged. Variable-temperature ^17^O NMR studies on the Co­(II) complexes confirmed that the complexes had a rapidly exchangeable water ligand even in the presence of metabolite. Interestingly, the presence of a single equivalent of lactate or pyruvate was sufficient to modulate the solubility of the [Fe­(L1)]^+^ complex, perhaps by preventing sulfonate-mediated binding or aggregation.

In contrast, NMR spectroscopy data were consistent with the formation of an inner-sphere fluoride for two of the Co­(II) complexes with binding constants of fluoride to [Co­(L1)]^+^ and [Co­(L2)]^2+^ similar to those observed for a Co­(II) complex with a tetradentate ligand.^38^ Interestingly, the Fe­(II) complexes bound fluoride in an inner-sphere interaction that was 10-fold tighter than either of the Co­(II) complexes. Thus, the small and negatively charged fluoride ion replaces a water molecule on both Co­(II) and Fe­(II) complexes to provide rare examples of Co–F or Fe–F complexes.^51^ Additional second-sphere fluoride interactions were supported by ^19^F NMR spectroscopy studies for both Fe­(II) and Co­(II) complexes of L1 and L2 as well as for [Co­(L3)]^2+^. Such interactions were absent in the coordinatively saturated complexes [Co­(L4)]^2+^ or [Fe­(L4)]^2+^. These studies show that ^19^F NMR spectroscopy is a useful tool to document second-sphere binding of anions as visualized by the paramagnetic broadening and shifting of resonances. In some cases, such as for [Fe­(L2)]^2+^, these shifts may be sufficient for the sensing of fluoride analytes.

Improved d-block parashift probes for metabolite sensing may require additional coordination sites or a less restricted coordination sphere for accomplishing metabolite binding. For example, paramagnetic Co­(II) complexes of TACN with sterically efficient amide pendants have two inner-sphere waters and may merit further development as responsive probes.^52^ Alternatively, Ni­(II) complexes of heptadentate macrocyclic ligands bind anions such as carbonate to give new paramagnetically shifted resonances, although the stability of the complex would need to be improved.^53,54^ Second, more highly symmetrical systems that give large numbers of equivalent protons would be advantageous to increase signal intensity or lower the limit of detection. One class of compounds under consideration in our laboratories is coordination cages of paramagnetic divalent metal ions that not only increase the parashift signal but also provide an alternative means of binding the metabolite by using host–guest interactions.^55^

## Experimental Section

All of the reagents and solvents used were of reagent grade and were used as obtained without further purification. TACN was purchased from Ambeed Chemicals. *Tert*-butyl alcohol was purchased from Acros Organics. 1,3-propane sultone and dimethylformamide-dimethyl acetal were purchased from TCI America. 2-Bromomethyl-6-methylpyridine was purchased from Sigma-Aldrich. Sodium carbonate, ferrous triflate, cobalt­(II) nitrate, perchlorate, and chloride were obtained from Fisher Scientific. Protected TACN and benzyl-TACN were prepared as published. The macrocycles (L2), [Fe­(L2)­(CF_3_SO_3_)]­(CF_3_SO_3_),^44^ and [Fe­(L4)]­(CF_3_SO_3_)_2_ ^19^ were prepared as reported.

### Safety Statement

Perchlorate salts of metal ions and their complexes may be explosive and should be handled with caution. The hazard was mitigated by working with milligram amounts of the crystals of the complexes.

### Instrumentation

^1^H and ^13^C NMR spectra were acquired on a Neo Bruker 500 MHz, at 25 °C. Chemical shift values (δ) were reported in parts per million and referenced to residual proteo-solvent peaks. Samples were run on a NEO-Bruker 500 MHz NMR equipped with a 5 mm broadband probe. All spectra were processed with MestReNova software. A Thermo Fisher Linear Ion Trap Quadrupole (LTQ) mass spectrometer was employed to monitor the progress of reactions or report the final mass-to-charge ratio of complexes. High-resolution mass spectrometry was obtained on a Thermo Fisher Q-Exactive Focus Orbitrap (MS component) associated with a Dionex Ultimate 3000 (HPLC). The iron or cobalt concentration of each of the complexes was determined using a Thermo X-Series 2 ICP-MS. Samples were dissolved in 65–70% metal-free nitric acid for 3 days to undergo digestion. After the digestion, a cobalt internal standard was added, and the samples were diluted with Milli-Q water to contain 2% nitric acid and 50 ppb cobalt. A linear calibration curve ranging from 0.1 to 100 ppb iron was also prepared and used to quantify the samples. Quantification and data analysis were performed using Thermo Fisher Plasma Lab. All pH measurements were recorded by using a Thermo Scientific 9110DJWP double-junction, glass, semimicro pH electrode connected to a 702 SM Titrino pH meter.

### Crystallization and X-ray Diffraction Data Collection

Uniform crystals of [Co­(L2)­(NO_3_)]­(NO_3_) were formed by a vapor diffusion method at room temperature, while growing over 3 days. In a typical procedure, around 5 mg of the complex was placed in a 0.5-dram vial and dissolved in 0.5 mL of methanol. The 0.5-dram vial was placed in a 20 mL scintillation vial containing 3 mL of diethyl ether. Suitable crystals were selected and mounted on glass fibers with oil on a Rigaku XtaLAB Synergy-S diffractometer installed at a rotating X-ray source (Ag Kα radiation, λ = 0.56087 Å). The crystals were kept at 100.15 K during the data collection. The structure was solved with the ShelXT structure solution program using the intrinsic phasing method and refined using the ShelXL refinement package using the least-squares minimization. The structure was refined by two-part disorder correction as one of the 6-methyl-2-picolyl methanol and nitrates bound and unbound to the metal center were disordered.

### Synthesis of 3-(Hexahydro-2a,4a,6a-triazacyclopenta­[cd]­pentalen-6a-Ium-6a­(2a1*H*)-yl) Propane-1-sulfonate (Protected TACN Sulfone)

0.5 g of protected TACN was treated with (1.13 equiv) 0.50 g of 1,3-propane sultone in the presence of Hunig’s base (DIPEA), acetonitrile at RT for 3 h to yield a white solid product. The product (white solid, 98% yield) was washed with acetonitrile and characterized. ESI-MS (positive) *m*/*z* calculated: 262.12 (M), found: 262.2 (100%), where M equals the ligand as drawn in Scheme 1. ^1^H NMR (D_2_O): 2.23–2.92 (2H, CH_2_, multiplet), 2.96–3.12 (2H, CH_2_, triplet), 3.14–3.19 (4H, TACN CH_2_, multiplet), 3.20–3.73 (8H, TACN CH_2_, multiplet), 3.73–3.80 (2H, CH_2_SO_3_H, triplet), 5.53 (1H, CH, singlet). ^13^C NMR (D_2_O): 20.11, 47.29, 51.04, 53.44, 57.63, 118.17 (Figures S75, S79).

**Scheme 1:**
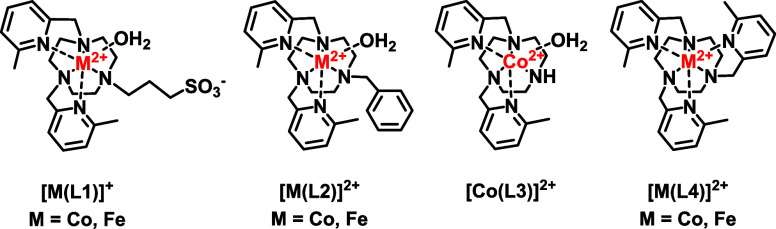
Parashift Agents

### Synthesis of 3-(1,4,7-Triazonan-1-yl)­propane-1-sulfonate (TACN Sulfone)

0.50 g of protected TACN-sulfone was treated with a 1:1 mixture of methanol and hydrobromic acid. The reaction was refluxed for 4 h, cooled to room temperature, and refrigerated overnight. Methanol was removed by distillation and the addition of cold anhydrous ethanol produced a white precipitate (yield 98%), which was filtered, washed with ethanol, and dried. ESI-MS (positive) *m*/*z* calculated: 251.13 (M), found: 252.2 (M + H^+^) (100%), where M equals the ligand as drawn in Scheme 1. ^1^H NMR (D_2_O): 1.91–1.93 (2H, CH_2_, multiplet), 2.79–2.81 (2H, CH_2_, triplet), 2.83–2.89 (2H, CH_2_SO_3_H, triplet), 2.95–2.99 (4H, TACN CH_2_, multiplet), 3.26–3.43 (4H, TACN CH_2_, multiplet), 3.53–3.60 (4H, TACN CH_2_, multiplet). ^13^C NMR (D_2_O): 20.00, 41.68, 43.24, 47.30, 49.10, 53.67 (Figures S76, S80).

### Synthesis of 3-(4,7-Bis­((6-methylpyridin-2-yl)­methyl)-1,4,7-triazonan-1-yl)­propane-1-sulfonic Acid (L1)

0.5 g of TACN-sulfone was treated with 2 equiv of 2-bromomethyl-6-methylpyridine overnight at 55 °C in the presence of base (Na_2_CO_3_, 1.2 mmol) and ethanol (10 mL), overnight. The solvent was removed under vacuum and then dichloromethane was added to dissolve the crude product. This solution was then centrifuged to remove the sodium carbonate, the supernatant was collected, and concentrated under vacuo to give a deep brown oil. The yield was 90%. ESI-MS (positive) *m*/*z* calculated: 461.3 (M), found: 462.2 (M) (100%), where M equals the ligand as drawn in Scheme 1. This was further confirmed by HRMS (Figure S73). ^1^H NMR (D_2_O): 1.64–1.83 (2H, CH_2_, multiplet), 2.69–2.75 (5H, TACN CH_2_, multiplet), 2.83–2.95 (7H, TACN CH_2_, multiplet), 3.27 (6H, CH_2_, singlet), 3.36 (4H, CH_2_, singlet), 3.37–3.58 (2H, CH_2_, triplet), 3.74–3.84 (2H, CH_2_SO_3_H, triplet), 7.30–7.51 (8H, benzyl CH, multiplet) (Figures S77, S81).

### Synthesis of [Fe­(L1)­(CF_3_SO_3_)]

L1 (0.2 g, 0.43 mmol) was treated with 1.05 equiv of ferrous triflate in methanol under argon and stirred overnight at room temperature. The volume of the solvent was reduced, and argon-purged diethyl ether was added dropwise to generate a shiny brown precipitate. The yield of the complex including one triflate counterion [Fe­(L1)­(CF_3_SO_3_)] was 53%. ESI-MS (positive) *m*/*z* calculated for the complex cation (C_23_H_34_N_5_O_3_SFe)^+^: 516.2 (M), found: 516.2 (M) and 517.2 (M + H^+^), where M equals the complex as drawn in Scheme S2. This was further confirmed by HRMS (Figure S83). ICP-MS: Fe content calculated assuming a triflate counterion (C_24_H_34_F_3_N_5_O_6_S_2_Fe): 8.39, found: 6.96.

### Synthesis of [(Co­(L1)­(ClO_4_)]

L1 (0.2 g, 0.43 mmol) was dissolved in 3 mL of methanol (yellowish-brown solution), and 1 mL of 1.05 equiv of cobalt­(II) perchlorate in methanol was added dropwise. The resultant mixture was stirred for 16 h and turned to olive green. Diethyl ether was added to the solution to precipitate the product, which was collected by centrifugation and dried under vacuum. The yield of the complex including one perchlorate [Co­(L1)­(ClO_4_)] was 95%. ESI-MS (positive) *m*/*z* calculated for the complex cation (C_23_H_34_N_5_O_3_SCo)^+^: 519.2 (M), found: 519.2 (M), and 520.1 (M + H^+^), where M equals the complex as drawn in Scheme S2.This was further confirmed by HRMS (Figure S84). ICP-MS: Co content calculated assuming a perchlorate counterion (C_23_H_34_ClN_5_O_7_SCo), 9.52, found: 8.50.

### Synthesis of [Co­(L2)­(NO_3_)]­(NO_3_)

L2 (103 mg, 0.24 mmol) in a solution of ethanol was placed in a double-necked round-bottom flask, and the solvent was evaporated under vacuum. Then, the final mass of the round-bottom flask was taken to obtain the ligand weight added to the flask. This flask was equipped with a stir bar and purged with argon gas for 15 min. Cobalt nitrate hexahydrate (1.05 equiv) was placed in a 20 mL vial and purged with argon gas for 15 min. Ethanol was added to a 20 mL vial and degassed using argon. The degassed ethanol was transferred to the round-bottom flask containing the ligand and to the 20 mL vial containing the cobalt salt through a cannula. After dissolving the cobalt salt in ethanol, the solution was transferred to the round-bottom flask through a cannula. The reaction was carried out in the round-bottom flask under an argon atmosphere for 2 h. The completion of the reaction was monitored by mass spectrometry, and diethyl ether was used to precipitate the product from the solution. The product (pink powder) was washed a couple of times with diethyl ether and dried under a vacuum. The yield of the complex including two nitrate counterions, [Co­(L2)­(NO_3_)]­(NO_3_), was 98.6%. HRMS *m*/*z* calculated for the complex cations (C_27_H_35_N_5_Co)^2+^: 244. 1106 (100%) [(M – 2­(NO_3_))/2]^2+^, 550.2096 (100%) [M – (NO_3_)]^1+^ (Figure S85), where M equals the complex as drawn in Scheme S4. ICP-MS: Co content calculated assuming two nitrate counterions (C_27_H_35_N_7_O_6_Co): 9.62, found: 9.59.

### Synthesis of L3 and L4 Ligands

1,4,7-Triazacyclononane (0.50 g, 3.86 mmol) was dissolved in acetonitrile (20 mL) to afford a colorless basic solution (pH ≈ 9). To this, 2-bromomethyl-6-methylpyridine (2.20 g, 11.6 mmol, 3.0 equiv) was added, resulting in a rose-pink solution (pH ≈ 6). Triethylamine (1.2 mL, 8.5 mmol, 2.2 equiv) was introduced gradually until pH ≈ 8. The mixture was heated at 70 °C for 6 h. The solvent was removed *in vacuo*, and the residue was dissolved in chloroform (20 mL) and washed with saturated brine (3 × 15 mL). The organic layer was dried (Na_2_SO_4_), filtered, and concentrated to yield an auburn-colored oil of crude L4.

Purification was achieved on a short plug of basic alumina (33 g, ≈20 × product mass). The column was pre-eluted with 0.5% MeOH/DCM to deactivate the resin. The crude was loaded in minimal 0.5% MeOH/DCM and eluted stepwise (0.5–3% MeOH/DCM, ∼300 mL per step). The tris product eluted primarily at 1%, with minor streak up to 2%, followed by the bis-substituted byproduct (>2%). Fractions were monitored by ESI-MS and NMR. A second brief column yielded analytically pure L3 and L4 as orange-amber gels (isolated yield ≈ 68%).

### **Ligand (L3)**: (C_20_H_29_N_5_)

ESI-MS (positive): calculated *m*/*z* = 339.2, found = 340.2 [M + H]^+^ (∼100%) (Figure S74, Scheme S5). ^1^H NMR (CDCl_3_): 2.44 (6H, CH_3_, singlet), 2.82 (12H, TACN CH_2_, singlet), 3.74 (4H, benzyl CH_2_, singlet), 6.90–6.92 (2H, benzyl CH, doublet), 7.13–7.14 (2H, benzyl CH, doublet), 7.39–7.42 (2H, benzyl CH, triplet). ^13^C NMR (CDCl_3_): 24.18, 45.63, 50.54, 53.04, 61.91, 119.84, 121.7, 136.79, 157.82, 158.27 (Figures S78, S82).

### **Ligand (L4)**: (C_27_H_36_N_6_)

ESI-MS (positive): calculated *m*/*z* = 444.3, found = 445.3 [M + H]^+^ (∼95%) and 467.3 [M + Na]^+^ (∼5%), where M corresponds to the product as drawn in Scheme S5. ^1^H NMR (CDCl_3_): 2.44 (9H, CH_3_, singlet), 2.57–2.75 (12H, TACN CH_2_, singlets), 3.74 (6H, benzyl CH_2_, singlet), 6.91–6.93 (2H, benzyl CH, doublet), 7.26–7.28 (2H, benzyl CH, doublet), 7.44–7.47 (2H, benzyl CH, triplet). ^13^C NMR (CDCl_3_): 24.57, 56.01, 65, 119.97, 121.23, 136.52, 157.39, 160.01.

### Synthesis of [Co­(L3)­(NO_3_)]­(NO_3_)

Ligand L3 (0.1 g, 0.3 mmol) was dissolved in acetonitrile (3 mL) to form a yellow solution. Cobalt­(II) nitrate hexahydrate (0.09 g, 0.3 mmol, 1.05 equiv) was added, producing a dark solution. The mixture was stirred at RT for 1 h and then layered with diethyl ether to afford a violet gel. The yield of the neutral complex including two nitrate counterions [Co­(L3)­(NO_3_)]­(NO_3_) was 95%. ESI-MS (positive) *m*/*z* calculated for the complex cations (C_20_H_29_N_5_Co)^2+^: 398.2 (M), found: 199 (M/2), where M equals the complex as drawn in Scheme S6. This was further confirmed by HRMS (Figure S86). ICP-MS: Co content calculated considering two nitrate counterions (C_20_H_29_N_7_O_6_Co): 11.28, found: 10.96.

### Synthesis of [Co­(L4)]­(ClO_4_)_2_

Ligand L4 (0.19 g, 0.42 mmol) was dissolved in acetonitrile (8 mL) to form a yellow solution. Cobalt­(II) perchlorate hexahydrate (0.17 g, 0.46 mmol, 1.1 equiv) was added, producing a rose-red solution. The mixture was stirred at RT for 1 h, concentrated *in vacuo* to 3 mL, then layered with diethyl ether to afford a purple precipitate. The yield of the complex including two perchlorate counterions [Co­(L4)]­(ClO_4_)_2_ was 95%. ESI-MS (positive) *m*/*z* calculated for the complex cations (C_27_H_36_N_6_Co)^2+^: 503.2 (M), found: 251.6 (M/2) and 602.2 [M + ClO_4_^–^]^+^, where M equals the complex as drawn in Scheme S8. ICP-MS: Co content calculated considering two perchlorate counterions (C_27_H_36_N_6_Cl_2_O_8_Co): 8.39, found: 8.01.

## Supplementary Material



## Notes

The SI includes Materials and methods, synthetic procedures, spectroscopic characterization, crystallographic data, NMR data as a function of temperature or addition of anions or metabolites (PDF)

## References

[1] Wahsner J., Gale E. M., Rodriguez-Rodriguez A., Caravan P. (2019). Chemistry of MRI Contrast
Agents: Current Challenges and New Frontiers. Chem. Rev..

[2] Caravan P., Ellison J. J., McMurry T. J., Lauffer R. B. (1999). Gadolinium
(III)
chelates as MRI contrast agents: structure, dynamics, and applications. Chem. Rev..

[3] Shuvaev S., Akam E., Caravan P. (2021). Molecular
MR Contrast Agents. Invest. Radiol..

[4] Bonnet C. S., Toth E. (2021). Metal-based environment-sensitive
MRI contrast agents. Curr. Opin. Chem. Biol..

[5] Major J. L., Meade T. J. (2009). Bioresponsive, cell-penetrating,
and multimeric MR
contrast agents. Acc. Chem. Res..

[6] De
Leon-Rodriguez L. M., Lubag A. J. M., Malloy C. R., Martinez G. V., Gillies R. J., Sherry A. D. (2009). Responsive MRI Agents for Sensing
Metabolism in Vivo. Acc. Chem. Res..

[7] Louie A. (2013). MRI biosensors:
A short primer. J. Magn. Reson. Imaging.

[8] Parrott D., Fernando W. S., Martins A. F. (2019). Smart MRI
Agents for Detecting Extracellular
Events In Vivo: Progress and Challenges. Inorganics.

[9] Li H., Meade T. J. (2019). Molecular magnetic
resonance imaging with Gd (III)-based
contrast agents: challenges and key advances. J. Am. Chem. Soc..

[10] Harnden A. C., Parker D., Rogers N. J. (2019). Employing
paramagnetic shift for
responsive MRI probes. Coord. Chem. Rev..

[11] Viswanathan S., Kovacs Z., Green K. N., Ratnakar S. J., Sherry A. D. (2010). Alternatives
to Gadolinium-Based Metal Chelates for Magnetic Resonance Imaging. Chem. Rev..

[12] Sherry A. D., Castelli D. D., Aime S. (2023). Prospects
and limitations of paramagnetic
chemical exchange saturation transfer agents serving as biological
reporters in vivo. NMR Biomed..

[13] Finney K.L.N., Harnden A.C., Rogers N.J., Senanayake P.K., Blamire A.M., O’Hogain D., Parker D. (2017). Simultaneous Triple
Imaging with Two PARASHIFT Probes: Encoding Anatomical, pH and Temperature
Information using Magnetic Resonance Shift Imaging. Chem. Eur. J..

[14] Huang Y., Coman D., Ali M. M., Hyder F. (2015). Lanthanide ion (III)
complexes of 1,4,7,10-tetraazacyclododecane-1,4,7,10-tetraaminophosphonate
for dual biosensing of pH with chemical exchange saturation transfer
(CEST) and biosensor imaging of redundant deviation in shifts (BIRDS). Contrast Media Mol. Imaging.

[15] Mishra S. K., Zakaria A., Mihailovic J., Maritim S., Mercado B., Coman D., Hyder F. (2024). Complexes
of Iron­(II), Cobalt­(II),
and Nickel­(II) with DOTA-Tetraglycinate for pH and Temperature Imaging
Using Hyperfine Shifts of an Amide Moiety. Inorg.
Chem..

[16] Slade F., Collingwood J. F., Rogers N. J. (2024). Transition metal
Parashift and ParaCEST
MRI agents: Current progress and challenges. Coord. Chem. Rev..

[17] Aime S., Botta M., Fasano M., Terreno E., Kinchesh P., Calabi L., Paleari L. (1996). A new ytterbium chelate as contrast
agent in chemical shift imaging and temperature sensitive probe for
MR spectroscopy. Magn. Reson. Med..

[18] Hekmatyar S. K., Hopewell P., Pakin S. K., Babsky A., Bansal N. (2005). Noninvasive
MR thermometry using paramagnetic lanthanide complexes of 1,4,7,10-tetraazacyclodoecane-alpha,alpha’,alpha’‘alpha’’’-tetramethyl-1,4,7,10-tetraacetic
acid (DOTMA4-). Magn. Reson. Med..

[19] Tsitovich P. B., Cox J. M., Benedict J. B., Morrow J. R. (2016). Six-coordinate Iron­(II)
and Cobalt­(II) paraSHIFT Agents for Measuring Temperature by Magnetic
Resonance Spectroscopy. Inorg. Chem..

[20] Bertini, I. ; Luchinat, C. Chapter 2 The Hyperfine Shift. Coord. Chem. Rev., 1996, 150, 29–75 10.1016/0010-8545(96)01242-8.

[21] Bertini, I. ; Luchinat, C. ; Parigi, G. ; Ravera, E. NMR of Paramagnetic Molecules Applications to Metallobiomolecules and Models; Elsevier, 2017; pp. 1–24.

[22] Harvey P., Blamire A. M., Wilson J. I., Finney K. L. N. A., Funk A. M., Senanayake P. K., Parker D. (2013). Moving the goal posts:
enhancing the sensitivity of PARASHIFT proton magnetic resonance imaging
and spectroscopy. Chem. Sci..

[23] Tsitovich P. B., Morrow J. R. (2012). Macrocyclic ligands
for Fe­(II) paraCEST and chemical
shift MRI contrast agents. Inorg. Chim. Acta.

[24] Tsitovich P. B., Tittiris T. Y., Cox J. M., Benedict J. B., Morrow J. R. (2018). Fe­(ii)
and Co­(ii) N-methylated CYCLEN complexes as paraSHIFT agents with
large temperature dependent shifts. Dalton Trans..

[25] Rogers N.
J., Kumar C. A., Alexander C., Bowdery D., Pavlovskaya G., Harvey P. (2025). Macrocyclic transition-metal parashift complexes for
MRI at clinical and pre-clinical magnetic fields. Dalton Trans..

[26] Morrow, J. R. ; Raymond, J. J. Other First-Row Transition Metal-Based Complexes as MRI Contrast Agents. In Lanthanide and Other Transition Metal Ion Complexes and Nanoparticles in Magnetic Resonance Imaging; Taylor & Francis, CRC Press, 2023; ch 6, pp 182–212.

[27] Tsitovich P. B., Gendron F., Nazarenko A. Y., Livesay B. N., Lopez A. P., Shores M. P., Autschbach J., Morrow J. R. (2018). Low-Spin Fe­(III)
Macrocyclic Complexes of Imidazole-Appended 1,4,7-Triazacyclononane
as Paramagnetic Probes. Inorg. Chem..

[28] Morrow, J. R. ; Chowdhury, S. I. ; Abozeid, S. M. ; Patel, A. ; Raymond, J. J. Transition metal ParaCEST, LipoCEST and CellCEST agents as MRI probes. In Encyclopedia of Inorganic and Bioinorganic Chemistry; John Wiley & Sons, 2020; pp. 1–19. 10.1002/9781119951438.eibc2749

[29] Martin B., Autschbach J. (2016). Kohn-Sham calculations of NMR shifts
for paramagnetic
3d metal complexes: protocols, delocalization error, and the curious
amide proton shifts of a high-spin iron­(II) macrocycle complex. Phys. Chem. Chem. Phys..

[30] Aime S., Delli Castelli D., Fedeli F., Terreno E. (2002). A paramagnetic MRI-CEST
agent responsive to lactate concentration. J.
Am. Chem. Soc..

[31] Aime S., Botta M., Mainero V., Terreno E. (2002). Separation of intra-
and extracellular lactate NMR signals using a lanthanide shift reagent. Magn. Reson. Med..

[32] Terreno E., Botta M., Fedeli F., Mondino B., Milone L., Aime S. (2003). Enantioselective recognition
between chiral α-hydroxy-carboxylates
and macrocyclic heptadentate lanthanide­(III) chelates. Inorg. Chem..

[33] Hammell J., Buttarazzi L., Huang C. H., Morrow J. R. (2011). Eu­(III) Complexes
as Anion-Responsive Luminescent Sensors and Paramagnetic Chemical
Exchange Saturation Transfer Agents. Inorg.
Chem..

[34] Chiaffarelli R., Cruz P. F., Cotton J., Kelm T., Lee S., Ghaderian M., Zimmermann M., Geraldes C. F. G. C., Jurek P., Martins A. F. (2025). Metabolic PCTA-Based Shift Reagents
for the Detection of Extracellular Lactate Using CEST MRI. JACS Au.

[35] Zhang L., Martins A. F., Zhao P. Y., Tieu M., Esteban-Gómez D., McCandless G. T., Platas-Iglesias C., Sherry A. D. (2017). Enantiomeric Recognition
of D- and L-Lactate by CEST with the Aid of a Paramagnetic Shift Reagent. J. Am. Chem. Soc..

[36] Bond C. J., Sokolow G. E., Crawley M. R., Burns P. J., Cox J. M., Mayilmurugan R., Morrow J. R. (2019). Exploring inner-sphere water interactions
of Fe (II) and Co (II) complexes of 12-membered macrocycles to develop
CEST MRI probes. Inorg. Chem..

[37] Jeon I. R., Park J. G., Haney C. R., Harris T. D. (2014). Spin crossover iron­(II)
complexes as PARACEST MRI thermometers. Chem.
Sci..

[38] O’Neill E. S., Kolanowski J. L., Bonnitcha P. D., New E. J. (2017). A cobalt­(II) complex
with unique paraSHIFT responses to anions. Chem.
Commun..

[39] Xie D., Yu M., Kadakia R. T., Que E. L. (2020). F-19 Magnetic Resonance Activity-Based
Sensing Using Paramagnetic Metals. Acc. Chem.
Res..

[40] Thorarinsdottir A. E., Gaudette A. I., Harris T. D. (2017). Spin-crossover and
high-spin iron­(II)
complexes as chemical shift 19-F magnetic resonance thermometers. Chem. Sci..

[41] Garda Z., Szeremeta F., Quin O., Molnár E., Váradi B., Clémencon R., Meme S., Pichon C., Tircsó G., Tóth E. (2024). Small Fluorinated Mn Chelate as an
Efficient ^1^H and ^19^F MRI Probe. Angew. Chem. Int. Ed..

[42] Garda Z., Szeremeta F., Tóth C. N., Bunda S., Pifferi C., Clémençon R., Même S., Tircsó G., Tóth É. (2025). Relaxation-Based
In Vivo Discrimination
of Oxidized and Reduced States of a Redox Switchable ^19^F MRI Probe. J. Am. Chem. Soc..

[43] Asik D., Smolinski R., Abozeid S. M., Mitchell T. B., Turowski S. G., Spernyak J. A., Morrow J. R. (2020). Modulating the properties of Fe (III)
macrocyclic MRI contrast agents by appending sulfonate or hydroxyl
groups. Molecules.

[44] Balaji D. K., Spernyak J. A., Crawley M. R., Morrow J. R. (2026). Redox-responsive
Fe­(II)/Fe­(III) MRI probes with pentadentate or hexadentate macrocyclic
ligands. Inorg. Chem. Front..

[45] Yang M. Y., Iranzo O., Richard J. P., Morrow J. R. (2005). Solvent deuterium
isotope effects on phosphodiester cleavage catalyzed by an extraordinarily
active Zn­(II) complex. J. Am. Chem. Soc..

[46] Dorazio S.
J., Tsitovich P. B., Siters K. E., Spernyak J. A., Morrow J. R. (2011). Iron­(II)
PARACEST MRI Contrast Agents. J. Am. Chem. Soc..

[47] Dorazio S.
J., Olatunde A. O., Spernyak J. A., Morrow J. R. (2013). CoCEST: Cobalt­(II)
amide-appended paraCEST MRI contrast agents. Chem. Commun..

[48] Alexander C., Li H. S., Harvey P., Pavlovskaya G. E., Rogers N. J., Parker D. (2026). Absolute Temperature
Mapping Using
Chiral Terbium Parashift Complexes for MRI Thermometry. Chem. Biomed. Imaging.

[49] Bloembergen N., Morgan L. O. (1961). Proton Relaxation Times in Paramagnetic Solutions Effects
of Electron Spin Relaxation. J. Chem. Phys..

[50] Bleaney B. (1972). Nuclear Magnetic-Resonance
Shifts in Solution Due to Lanthanide Ions. J.
Magn. Reson..

[51] Blower P. J., Levason W., Luthra S. K., McRobbie G., Monzittu F. M., Mules T. O., Reid G., Subhan M. N. (2019). Exploring
transition
metal fluoride chelates – synthesis, properties and prospects
towards potential PET probes. Dalton Trans..

[52] Abozeid S. M., Snyder E. M., Tittiris T. Y., Steuerwald C. M., Nazarenko A. Y., Morrow J. R. (2018). Inner-sphere and
outer-sphere water
interactions in Co (II) paraCEST agents. Inorg.
Chem..

[53] Olatunde A. O., Cox J. M., Daddario M. D., Spernyak J. A., Benedict J. B., Morrow J. R. (2014). Seven-Coordinate Co-II Fe-II and
Six-Coordinate Ni-II
Amide-Appended Macrocyclic Complexes as ParaCEST Agents in Biological
Media. Inorg. Chem..

[54] Olatunde A. O., Dorazio S. J., Spernyak J. A., Morrow J. R. (2012). The NiCEST Approach:
Nickel­(II) ParaCEST MRI Contrast Agents. J.
Am. Chem. Soc..

[55] Dissanayake A., Raymond J. J., Crawley M. R., Morrow J. R. (2026). Octahedral Coordination
Cages of Co­(II) and Ni­(II): Enhanced Probe Signal Through Symmetry. Inorg. Chem..

