# Supplementary material for: Inner- and Second-Sphere Interactions of Co(II) and Fe(II) Parashift Agents with Lactate, Trifluorolactate, and Fluoride
Source: Inorg Chem. 2026 Jun 15;65(25):14348–60. doi: 10.1021/acs.inorgchem.6c01979 (PMC13321298; doi:10.1021/acs.inorgchem.6c01979)
Supplement: Supplementary file 1 [file ic6c01979_si_001.pdf]

Supporting Information

Inner- and Second-sphere Interactions of Co(II) and Fe(II) Parashift Agents with Lactate, Trifluorolactate and Fluoride

Anwita Roy,[1+] Deepak Krishnan Balaji,[1+] Joseph A. Spernyak[2] and Janet R. Morrow*[1]

1. Department of Chemistry, University at Buffalo, The State University of New York

Amherst, NY 14260, United States

2. Department of Cell Stress Biology, Roswell Park Comprehensive Cancer Center,

Buffalo, New York 14263, United States

+ contributed equally

*corresponding author, jmorrow@buffalo.edu

List of figures

Figure S 1: ^{1}H NMR experiment used to calculate magnetic susceptibility for 10 mM $[Fe(L1)]^{+}$ in 5% t-butanol in D_2O. 21
Figure S 2: ^{1}H NMR experiment used to calculate magnetic susceptibility for 10 mM $[Co(L1)]^{+}$ in 5% t-butanol in D_2O. With counteranions (A) chloride, (B) perchlorate and (C) nitrate. 21
Figure S 3: ^{1}H NMR experiment used to calculate magnetic susceptibility for 10 mM $[Co(L2)]^{2+}$ in 5% t-butanol in D_2O. 22
Figure S 4: ^{1}H NMR experiment used to calculate magnetic susceptibility for 10 mM $[Co(L3)]^{2+}$ in 5% t-butanol in D_2O. 22
Figure S 5: UV-Vis spectra of $[Co(L1)]^{+}$ in the presence of HEPES buffer over 4 hours. Conditions: 20 mM complex, 20 mM HEPES, 0.1 M NaCl, pH 7.4, 37 °C. 23
Figure S 6: UV-Vis spectra of $[Co(L1)]^{+}$ in the presence of Zn^{2+} over 4 hours. Conditions: 20 mM complex, 20 mM HEPES, 0.1 M NaCl, 20 mM $Zn(NO_3)_2$. $6H_2O$, pH 7.4, 37 °C. 23
Figure S 7: UV-Vis spectra of $[Co(L2)]^{2+}$ in the presence of HEPES buffer over 4 hours. Conditions: 10 mM complex, 20 mM HEPES, 0.1 M NaCl, pH 7.4, 37 °C. 24
Figure S 8: UV-Vis spectra of $[Co(L2)]^{2+}$ in the presence of Zn^{2+} over 4 hours. Conditions: 10 mM complex, 20 mM HEPES, 0.1 M NaCl, 10 mM $Zn(NO_3)_2$, pH 7.4, 37 °C. 24
Figure S 9: UV-Vis spectra of $[Co(L3)]^{2+}$ in the presence of HEPES buffer over 4 hours. Conditions: 10 mM complex, 20 mM HEPES, 0.1 M NaCl, pH 7.4, 37° C. 25
Figure S 10: UV-Vis spectra of $[Co(L3)]^{2+}$ in the presence of Zn^{2+} over 4 hours. Conditions: 10 mM complex, 20 mM HEPES, 0.1 M NaCl, 10 mM $Zn(NO_3)_2$, pH 7.4, 37 °C. 25
Figure S 11: UV-Vis spectrum of $[Fe(L1)]^{+}$. Conditions: 0.20 mM complex, 0.1 M NaCl, pH 4.0, 37 °C. 26
Figure S 12: ^{1}H NMR spectrum of $[Fe(L1)]^{+}$ in D_2O. Conditions: 10 mM, pD 4.4 26
Figure S 13: ^{1}H NMR spectrum of $[Co(L1)]^{+}$ in D_2O. Conditions: 10 mM, pD 7.8 27
Figure S 14: ^{1}H NMR spectrum of $[Co(L2)]^{2+}$ in D_2O. Conditions: 10 mM, pD 7.8 27
Figure S 15: ^{1}H NMR spectrum of $[Co(L3)]^{2+}$ in D_2O. Conditions: 10 mM, pD 7.8 28
Figure S 16: Variable temperature paramagnetic ^{1}H NMR spectra of $[Co(L1)]^{+}$. Bottom (25 °C) to top (75 °C) with 5°C interval. Conditions: 10 mM, D_2O, pD 7.8. 29
Figure S 17: Temperature dependence of ^{1}H chemical shifts of $[Co(L1)]^{+}$. Conditions: 10 mM, D_2O, pD 7.8 29
Figure S 18: Variable temperature paramagnetic ^{1}H NMR spectra of $[Co(L2)]^{2+}$. Bottom (25 °C) to top (75 °C) with 5°C interval. Conditions: 10 mM, D_2O, pD 7.8. 30
Figure S 19: Temperature dependence of ^{1}H chemical shifts of $[Co(L2)]^{2+}$. Conditions: 10 mM, D_2O, pD 7.8 31
Figure S 20: Variable temperature paramagnetic ^{1}H NMR spectra of $[Co(L3)]^{2+}$. Bottom (25 °C) to top (75 °C) with 5°C interval. Conditions: 10 mM, D_2O, pD 7.8. 32
Figure S 21: Temperature dependence of selected ^{1}H chemical shifts of $[Co(L3)]^{2+}$. Conditions: 10 mM, D_2O, pD 7.8. 32

Figure S 22: ^{1}H NMR spectra to study detection limit of $[Fe(L1)]^{+}$. Conditions: 1 mM - 10 mM complex, pD 4.4, D_2O. 33
Figure S 23: ^{1}H NMR spectra to study limit of detection of $[Fe(L2)]^{2+}$. Conditions: 0.05 mM - 5 mM complex, pD 7.8, D_2O. 34
Figure S 24: ^{1}H NMR spectra to study limit of detection of $[Co(L1)]^{+}$. Conditions: 0.125 mM - 10 mM complex, pD 7.8, D_2O. 34
Figure S 25: ^{1}H NMR spectra to study limit of detection of $[Co(L2)]^{2+}$. Conditions: 0.063 mM - 10 mM complex, pD 7.8, D_2O. 35
Figure S 26: ^{1}H NMR spectra to study limit of detection of $[Co(L3)]^{2+}$. Conditions: 0.13 mM - 10 mM complex, pD 7.8, D_2O. 35
Figure S 27: ^{1}H NMR spectra to study limit of detection of $[Fe(L4)]^{2+}$. Conditions: 0.025 mM - 10 mM complex, pD 7.8, D_2O. 36
Figure S 28: ^{1}H NMR spectra to study limit of detection of $[Co(L4)]^{2+}$. Conditions: 0.008 mM - 10 mM complex, pD 7.8, D_2O. 36
Figure S 29: Variable temperature ^{17}O NMR spectra of aqueous solutions of $[Co(L1)]^{+}$. Conditions: 10 mM complex, 0.1 M NaCl, pH 7.4, 1% (v/v) $H_2{}^{17}O$. 38
Figure S 30: Variable temperature ^{17}O NMR spectra of aqueous solutions of $[Co(L1)]^{+}$ with (A) 10 mM KF (B) 10 mM lactate (C) 10 mM pyruvate & (D) 10 mM malate on left and Swift-Connick fitting of $\ln(1/T_{2r})$ vs 1/T on right. Conditions: 10 mM complex, 0.1 M NaCl, pH 7.4, 1% (v/v) $H_2{}^{17}O$. 39
Figure S 31: Variable temperature ^{17}O NMR spectra of solutions containing $[Co(L2)]^{2+}$ on left and Swift-Connick fitting of $\ln(1/T_{2r})$ vs 1/T on right. Conditions: 10 mM complex, 0.1 M NaCl, pH 7.4, 1% (v/v) $H_2{}^{17}O$. 40
Figure S 32: Variable temperature ^{17}O NMR spectra of solutions of $[Co(L3)]^{2+}$ on left and Swift-Connick fitting of $\ln(1/T_{2r})$ vs 1/T on right. Conditions: 10 mM complex, 0.1 M NaCl, pH 7.4, 1% (v/v) $H_2{}^{17}O$. 41
Figure S 33: Variable temperature ^{17}O NMR spectra of solutions containing: (A) $[Fe(L1)]^{+}$, with (B) 10 mM lactate (C) 10 mM pyruvate & (D) 100 mM pyruvate. Conditions: 10 mM complex, 0.1 M NaCl, pH 4 (A), pH 7.4 (B, C, D), 1% (v/v) $H_2{}^{17}O$. 43
Figure S 34: ^{1}H NMR spectra of $[Co(L1)]^{+}$ (black) upon addition of 10 mM lactate (green). Conditions: 10 mM complex, pD 7.8, D_2O. 44
Figure S 35: ^{1}H NMR spectra of $[Co(L1)]^{+}$ (black) upon addition of 10 mM pyruvate (pink). Conditions: 10 mM complex, pD 7.8, D_2O. 44
Figure S 36: ^{1}H NMR spectra of $[Co(L1)]^{+}$ (black) upon addition of 10 mM malate (blue). Conditions: 10 mM complex, pD 7.8, D_2O. 45
Figure S 37: ^{1}H NMR spectra of $[Co(L1)]^{+}$ (black) upon addition of 10 mM trifluorolactate (orange). Conditions: 10 mM complex, pD 7.8 D_2O (left), pH 7.4 H_2O (right). 45
Figure S 38: ^{1}H NMR spectra of $[Co(L1)]^{+}$ upon addition of 10 eq. KF. Conditions: 10 mM complex, 100 mM KF, pD 7.8 D_2O. New proton resonances are labeled. 46

Figure S 39: ^{1}H NMR spectra of $[Co(L2)]^{2+}$ (black) upon addition of 10 mM lactate (red). Conditions: 10 mM complex, pD 7.8, D_2O. 46
Figure S 40: ^{1}H NMR spectra of $[Co(L2)]^{2+}$ (black) upon addition of 10 mM pyruvate (red). Conditions: 10 mM complex, pD 7.8, D_2O. 47
Figure S 41: ^{1}H NMR spectra of $[Co(L2)]^{2+}$ (black) upon addition of 10 mM trifluorolactate (red). Conditions: 10 mM complex, pD 7.8, D_2O. 47
Figure S 42: ^{1}H NMR spectra of $[Co(L3)]^{2+}$ (black) upon addition of 10 mM KF (red). Conditions: 10 mM complex, pD 7.8, D_2O. 48
Figure S 43: ^{1}H NMR spectra of $[Co(L3)]^{2+}$ (black) upon addition of 10 mM trifluorolactate (orange). Conditions: 10 mM complex, pD 7.8, D_2O. 48
Figure S 44: ^{1}H NMR spectra of $[Fe(L4)]^{2+}$ (black) upon addition of 10 mM KF (red). Conditions: 10 mM complex, pD 7.8, D_2O. 49
Figure S 45: ^{1}H NMR spectra of $[Fe(L4)]^{2+}$ (black) upon addition of 10 mM trifluorolactate (orange). Conditions: 10 mM complex, pD 7.8, D_2O. 49
Figure S 46: ^{1}H NMR spectra of $[Co(L4)]^{2+}$ (black) upon addition of 10 mM trifluorolactate (orange). Conditions: 10 mM complex, pD 7.8, D_2O. 50
Figure S 47: ^{1}H NMR spectra of $[Fe(L1)]^{+}$ (10 mM, black pD 4.4) upon addition of 10 mM lactate (green pD 7.8). Conditions: D_2O. 50
Figure S 48: ^{1}H NMR spectra of $[Fe(L1)]^{+}$ (10 mM, black pD 4.4) upon addition of 10 mM pyruvate (pink pD 7.8). Conditions: D_2O. 51
Figure S 49: ^{1}H NMR spectra of $[Fe(L2)]^{2+}$ (black) upon addition of 10 mM trifluorolactate (red). Conditions: 10 mM complex, pD 7.8, D_2O. 51
Figure S 50: ^{1}H NMR spectra of $[Fe(L2)]^{2+}$ (black) upon addition of 25 mM KF (red). Conditions: 5 mM complex, pD 7.8 D_2O (top), pH 7.4 H_2O (bottom). New proton resonances are labeled. In H_2O, there is a new resonance at about 200 ppm that does not appear in D2O which is assigned as a protonated group. 52
Figure S 51: High resolution mass spectrometry of $[Fe(L2)]^{2+}$ upon addition of 5 eq. KF Conditions: 100 μM complex, 500 μM KF, pH 7.4 H_2O. Insert shows the simulated spectra of $[Fe(L2)(F)]^{+}$ species. 53
Figure S 52: ^{19}F NMR spectra for titration of $[Fe(L2)]^{2+}$ with fluoride. Conditions: 5 mM complex, 1 - 25 mM KF, pH 7.4, H_2O, 0.1 M NaCl. 54
Figure S 53: Plot of change in chemical shift of ^{19}F NMR vs concentration of KF with fitting curve to determine binding constant for $[Fe(L2)]^{2+}$ with fluoride. pD 7.8 D_2O (left), pH 7.4 H_2O (right). 54
Figure S 54: ^{19}F NMR spectra for titration of fluoride with $[Fe(L1)]^{+}$. Conditions: 0 – 10 mM complex, 10 mM KF, pD 7.8, D_2O, 0.1 M NaCl. 55
Figure S 55: Plot of change in linewidth of ^{19}F NMR vs concentration from the titration of fluoride with $[Fe(L1)]^{+}$ 55
Figure S 56: ^{19}F NMR spectra for titration of fluoride with $[Co(L1)]^{+}$. Conditions: 0 – 10 mM complex, 10 mM KF, pD 7.8, D_2O, 0.1 M NaCl. 56

Figure S 57: Plot of change in linewidth of ^{19}F NMR vs concentration from the titration of fluoride with $[Co(L1)]^{+}$ 56
Figure S 58: ^{19}F NMR titration of fluoride with $[Co(L1)]^{+}$. Conditions: 10 mM complex, 1 - 20 mM KF, 3 mM trifluoroethanol, pD 7.8, D_2O, 0.1 M NaCl. 57
Figure S 59: ^{19}F NMR titration of fluoride with $[Co(L3)]^{2+}$. Conditions: 0 – 10 mM complex, 10 mM KF, pD 7.8, D_2O, 0.1 M NaCl. 57
Figure S 60: Plot of change in linewidth of ^{19}F NMR vs concentration from the titration of fluoride with $[Co(L3)]^{2+}$. 58
Figure S 61: ^{19}F NMR titration of $[Co(L3)]^{2+}$ with fluoride. Conditions: 10 mM complex, 10 - 100 mM KF, pD 7.8, D_2O, 0.1 M NaCl. 58
Figure S 62: ^{19}F NMR titration of $[Fe(L4)]^{2+}$ with fluoride. Conditions: 10 mM complex, 1 - 20 mM KF, 3 mM trifluoroethanol, pD 7.8, D_2O, 0.1 M NaCl. 59
Figure S 63: ^{19}F NMR spectra of titration of $[Fe(L2)]^{2+}$ and trifluorolactate. Conditions: 5 mM complex, 1 -10 mM trifluorolactate, pD 7.8, D_2O, 0.1 M NaCl. 59
Figure S 64: Plot of change in linewidth of ^{19}F NMR vs concentration from the titration of $[Co(L1)]^{+}$ with trifluorolactate. D_2O (left) and H_2O (right). 60
Figure S 65: ^{19}F NMR spectra for titration of trifluorolactate with $[Co(L1)]^{+}$. Conditions: 0 – 10 mM complex, 10 mM trifluorolactate, pH 7.4, H_2O, 0.1 M NaCl. 60
Figure S 66: ^{19}F NMR spectra of titration of $[Co(L1)]^{+}$ and trifluorolactate. Conditions: 10 mM complex, 10 -100 mM trifluorolactate, pD 7.8, D_2O, 0.1 M NaCl. Impurity in trifluorolactate is marked by a star. 61
Figure S 67: ^{19}F NMR spectra of titration of $[Co(L2)]^{2+}$ and trifluorolactate. Conditions: 10 mM complex, 10 -100 mM trifluorolactate, pD 7.8, D_2O, 0.1 M NaCl. Impurity in trifluorolactate is marked by a star. 61
Figure S 68: ^{19}F NMR titration of 10 mM trifluorolactate with $[Co(L3)]^{2+}$. Conditions: complex 0 – 10 mM, pD 7.8, D_2O, 0.1 M NaCl. 62
Figure S 69: Plot of change in linewidth of ^{19}F NMR vs concentration from the titration of $[Co(L3)]^{2+}$ with trifluorolactate. 62
Figure S 70: ^{19}F NMR titration of 10 mM trifluorolactate with $[Fe(L4)]^{2+}$. Conditions: complex 0 – 10 mM, pD 7.8, D_2O, 0.1 M NaCl. 63
Figure S 71: ^{19}F NMR spectra of titration of $[Fe(L4)]^{2+}$ and trifluorolactate. Conditions: 10 mM complex, 10 -100 mM trifluorolactate, pD 7.8, D_2O, 0.1 M NaCl. 63
Figure S 72: ^{19}F NMR titration of 10 mM trifluorolactate with $[Co(L4)]^{2+}$. Conditions: complex 0 – 10 mM, pD 7.8, D_2O, 0.1 M NaCl. 64
Figure S 73: High-resolution mass spectrometry of L1 ligand. 65
Figure S 74: High-resolution mass spectrometry of L3 ligand. 65
Figure S 75: ^{1}H NMR spectrum of Protected sulfone TACN in D_2O. Conditions: 20mM 66
Figure S 76: ^{1}H NMR spectrum of sulfone TACN in D_2O. Conditions: 20mM 66
Figure S 77: ^{1}H NMR spectrum of L1 ligand in D_2O. Conditions: 20mM 67
Figure S 78: ^{1}H NMR spectrum of L3 ligand in $CDCl_3$. Conditions: 20mM 67

Figure S 79: ^{13}C NMR spectrum of Protected sulfone TACN in D_2O. Conditions: 20mM 68
Figure S 80: ^{13}C NMR spectrum of sulfone TACN in D_2O. Conditions: 20mM 68
Figure S 81: ^{13}C NMR spectrum of L1 ligand in D_2O. Conditions: 20mM 69
Figure S 82: ^{13}C NMR spectrum of L3 ligand in $CDCl_3$. Conditions: 20mM............................. 69
Figure S 83: High-resolution mass spectrometry of $[Fe(L1)]^+$. 70
Figure S 84: High-resolution mass spectrometry of $[Co(L1)]^+$. 70
Figure S 85: High-resolution mass spectrometry of $[Co(L2)]^{2+}$. 71
Figure S 86: High-resolution mass spectrometry of $[Co(L3)]^{2+}$. 71

List of tables

Table S1: Acquisition parameters for parashift imaging. 16
Table S 2: Crystal data and structure refinement for [Co(L2)(NO_3)](NO_3). CCDC Deposition Number 2545340 17
Table S 3: Bond lengths for [Co(L2)(NO_3)](NO_3). 18
Table S 4: Bond angles for [Co(L2)(NO_3)](NO_3). 19
Table S5: Assignment of proton resonances of $[Co(L1)]^{+}$ based on R_1, R_2 and integration. 28
Table S6: Assignment of proton resonances of $[Co(L2)]^{2+}$ based on R_1, R_2 and integration 28
Table S7: Slopes from temperature dependence of ^{1}H chemical shifts of $[Co(L1)]^{+}$. Conditions: 10 mM, D_2O, pD 7.8. 30
Table S8: Temperature dependence of ^{1}H chemical shifts of $[Co(L2)]^{2+}$. Conditions: 10 mM, D_2O, pD 7.8 31
Table S9: Temperature dependence of ^{1}H chemical shifts of $[Co(L3)]^{2+}$. Conditions: 10 mM, D_2O, pD 7.8 33
Table S10: ^{17}O NMR data for 10 mM $[Co(L1)]^{+}$ solutions with and without 10 mM metabolites or fluoride 37
Table S11: Calculated rate constant and activation parameters for water exchange in solutions containing 10 mM $[Co(L1)]^{+}$ with and without 10 mM metabolites or fluoride. 37
Table S12: ^{17}O NMR data for solutions containing 10 mM $[Co(L2)]^{2+}$. 40
Table S 13: Calculated rate constant and activation parameters for water exchange in solutions of 10 mM $[Co(L2)]^{2+}$ 40
Table S14: ^{17}O NMR data for solutions containing 10 mM $[Co(L3)]^{2+}$. 41
Table S15: Calculated rate constant and activation parameters for water exchange in solutions containing 10 mM $[Co(L3)]^{2+}$ 41
Table S16: ^{17}O NMR data for solutions of 10 mM $[Fe(L1)]^{+}$ with and without 10 mM metabolite anions. 42
Table S17: Calculated rate constant and activation parameters for water exchange in solutions of 10 mM $[Fe(L1)]^{+}$ 42
Table S 18: Binding constants from the fluoride NMR titrations at 25 °C, 0.1 M NaCl, pD 7.8. 53

Schemes

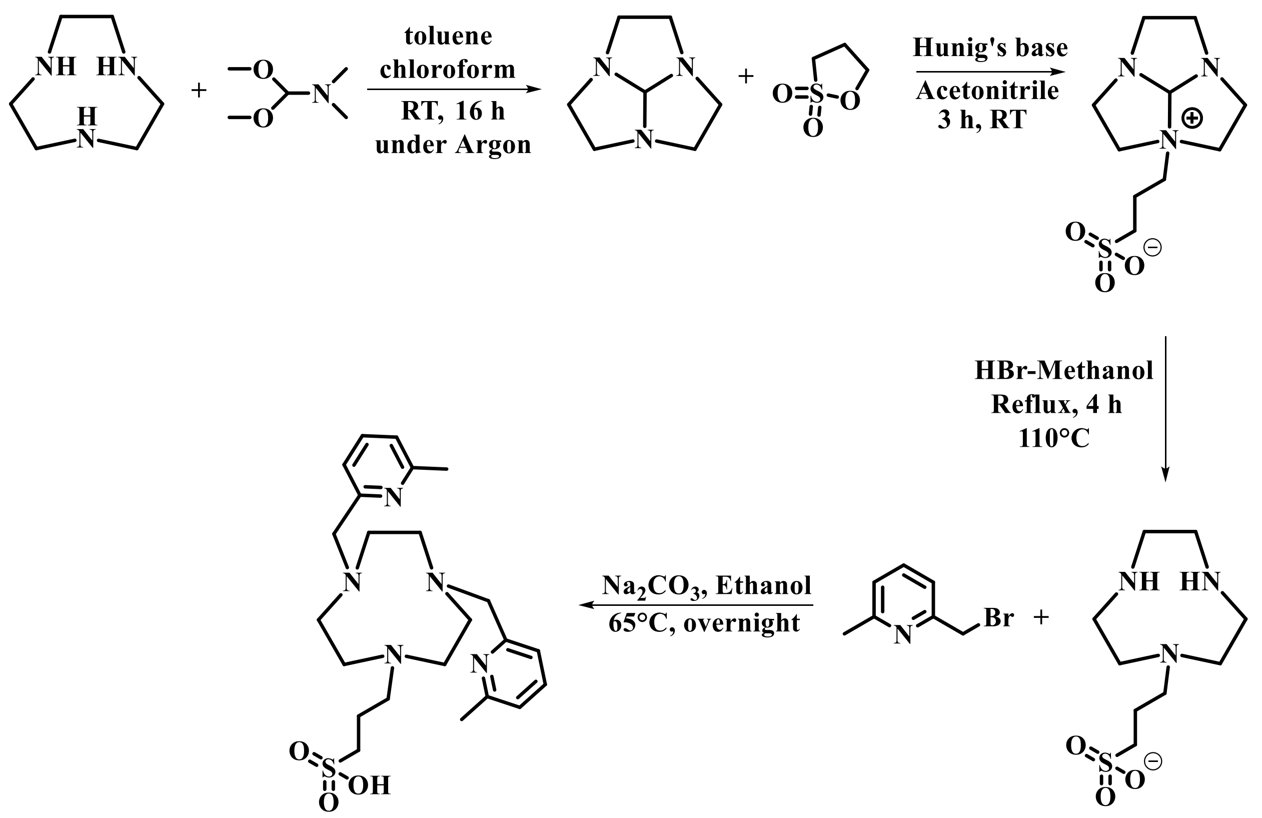

Scheme S 1: Synthesis of ligand L1.

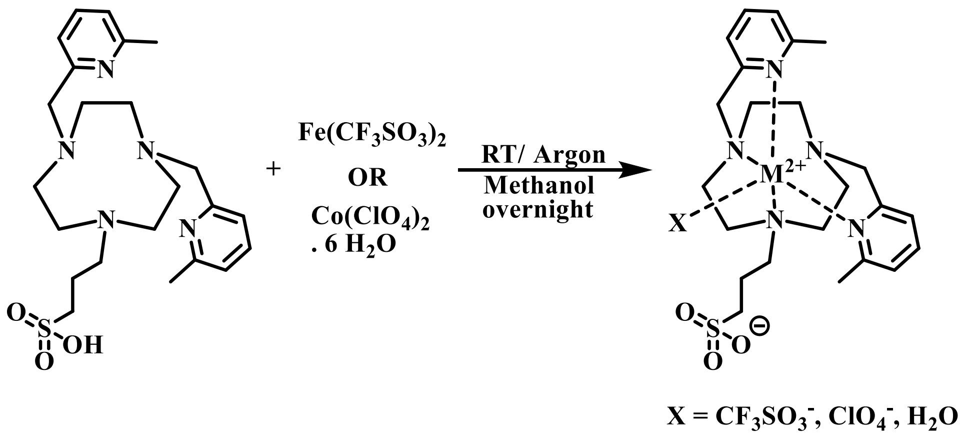

Scheme S 2: Synthesis of $[Fe(L1)(CF_3SO_3)]$ and $[Co(L1)(ClO_4)]$ complexes.

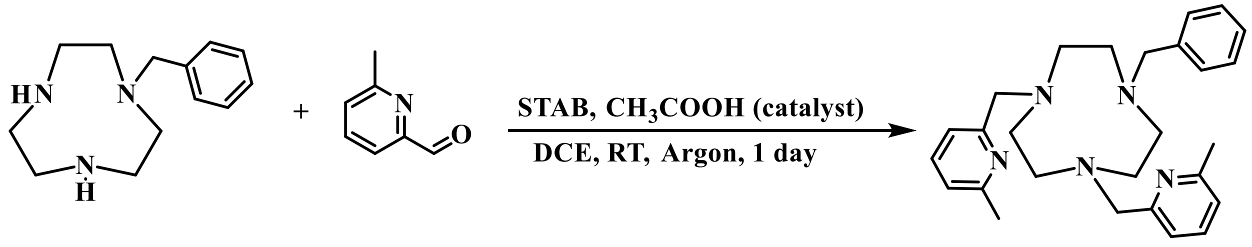

Scheme S 3: Synthesis of ligand L2.

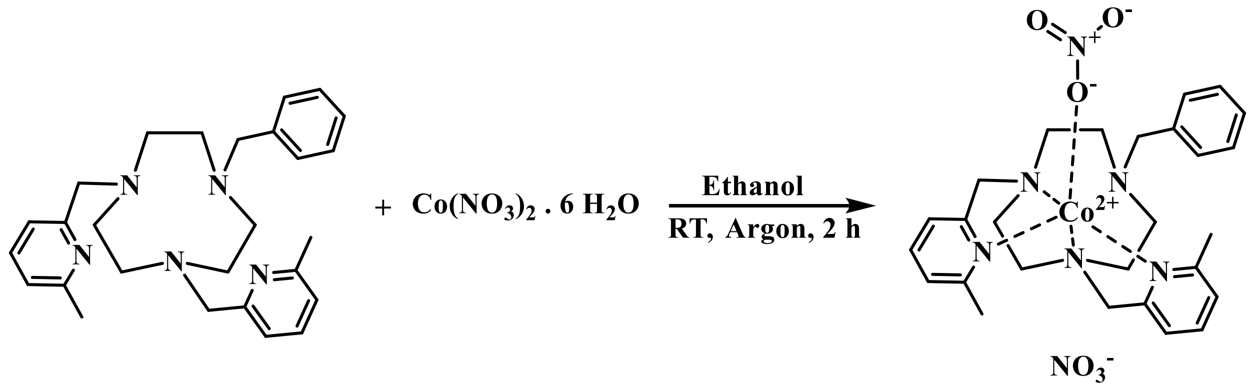

Scheme S 4: Synthesis of $[Co(L2)(NO_3)](NO_3)$ complex.

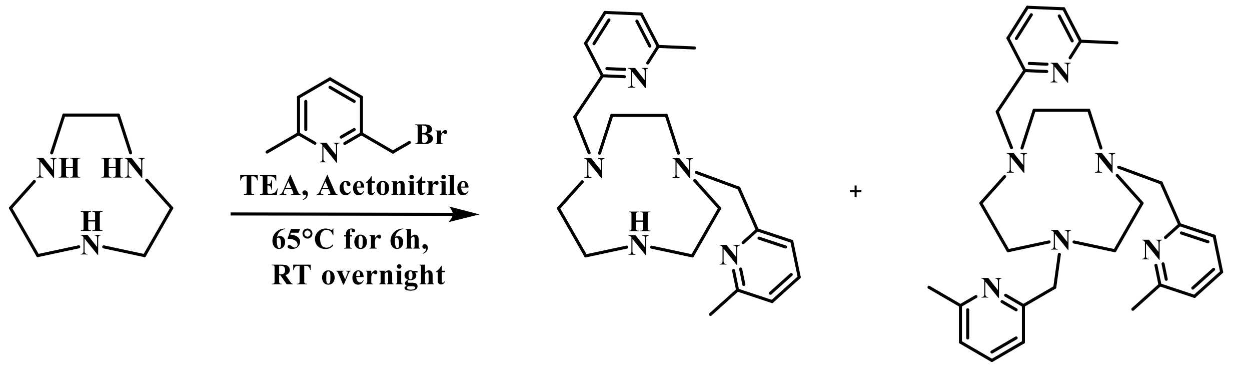

L3

L4

Scheme S 5: Synthesis of ligands L3 and L4.

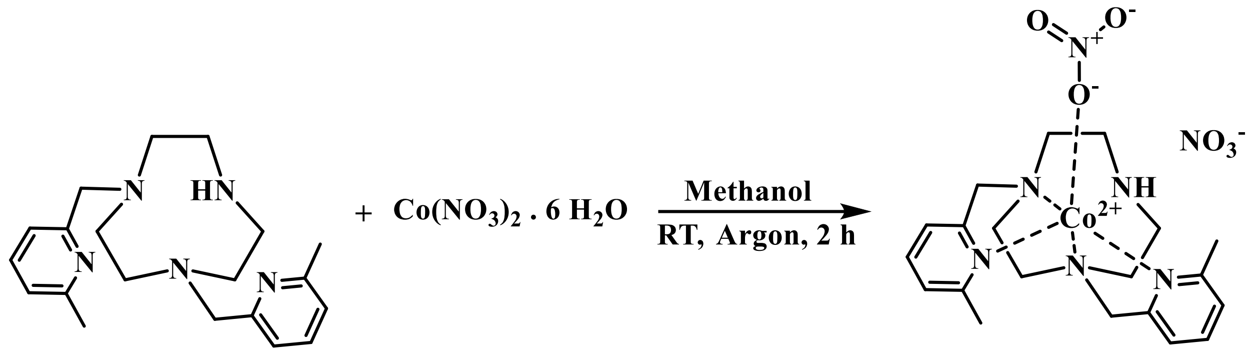

Scheme S 6: Synthesis of $[Co(L3)(NO_3)](NO_3)$ complex.

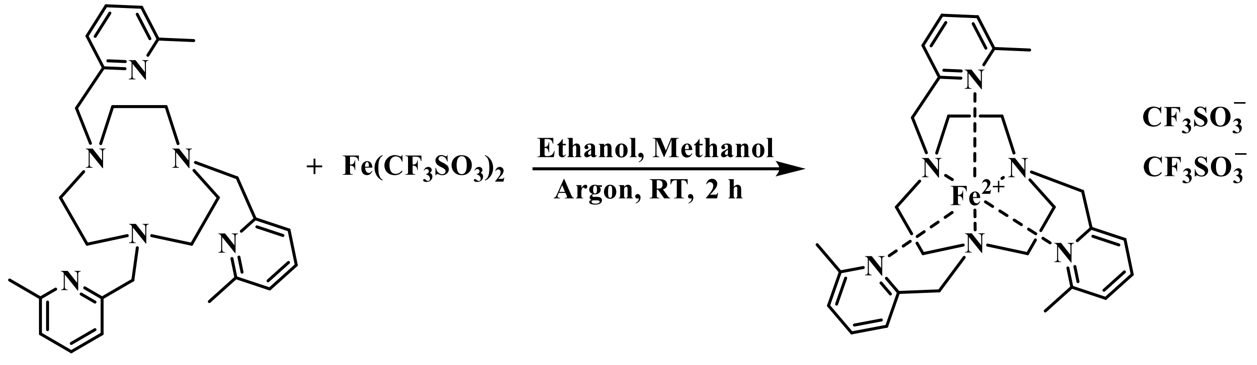

Scheme S 7: Synthesis of $[Fe(L4)](CF_3SO_3)_2$ complex.

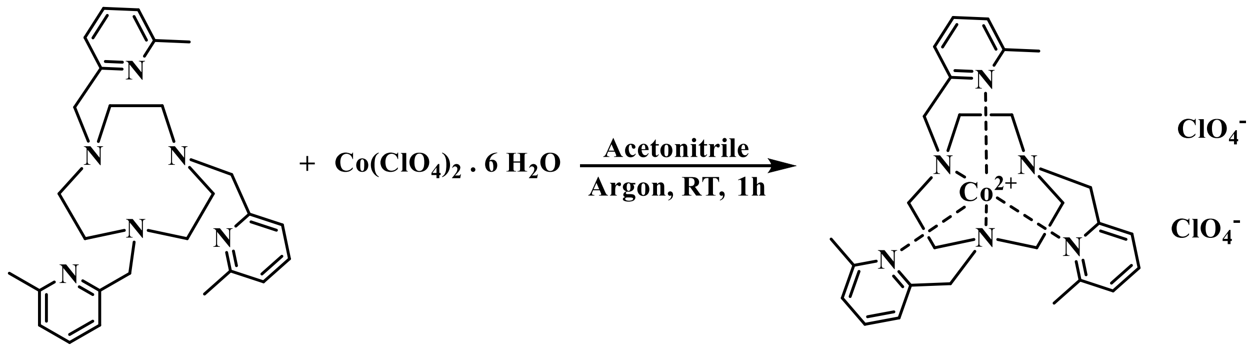

Scheme S 8: Synthesis of $[Co(L4)](ClO_4)_2$ complex.

Methods

Evans's method for determination of magnetic moments

Evans's method for determining magnetic susceptibility (χ) was performed as reported.[1] Three independently measured values were averaged. In a typical experiment, a solution of 10 mM Fe(II) or Co(II) complex in water containing 5% tert-butanol (v/v) was placed in a coaxial NMR tube insert while a reference solution of 5% tert-butanol (v/v) in D_2O was contained in the "outer" NMR tube. The mass susceptibility (χ_g) was calculated using Equation 1, where Δf is the frequency shift (Hz); v_0 is the operating frequency of the NMR spectrometer (Hz); and m is the concentration of the substance (g/mL). The solvent correction was considered using Equation 2. The molar susceptibility (χ_M) was calculated by multiplying the mass susceptibility by the molar mass of the complex. Then the paramagnetic molar susceptibility (χ_M^P) was calculated by subtracting the diamagnetic susceptibility contribution (χ_M^{dia}) in Equations 3 – 4. This was used to calculate the effective magnetic moment (μeff) in Equation 5.

$$\chi_g = \left(\frac{3\Delta f}{4\pi v_0 m}\right) + \chi_0 \qquad \textit{Eq. 1}$$

$$\chi_M = \chi_g * MW \qquad \textit{Eq. 2}$$

$$\chi_M^{dia} = -\left(\frac{MW}{2}\right) * 10^{-6} \qquad \textit{Eq. 3}$$

$$\chi_M^p = \chi_M - \chi_M^{dia} \qquad \textit{Eq. 4}$$

$$\mu_{eff} = 2.83\sqrt{\left(\chi_M^p * T\right)} \qquad \textit{Eq. 5}$$

Variable-Temperature ^{17}O NMR Spectroscopy:

The samples were prepared in 0.1 M sodium chloride in 1% $H_2{}^{17}O$ water solution, pH was adjusted by adding 0.1 M NaOH and studied at temperatures ranging from 298 K – 353 K. The transverse relaxation rate constants ($\frac{1}{T_{2r}}$) were calculated by the difference of the full width at half maximum (FWHM) of the ^{17}O resonance with and without Fe(II)/Co(II) complex and multiplying by a factor of π and dividing by the mole fraction of bound water P_m. Linewidth and chemical shift values for each spectrum were collected from the instrument and used in the Swift-Connick Eq. 6 – 11 to calculate the water exchange rate.

$$\frac{1}{T_{2r}} = \frac{\pi}{P_m} * (\Delta\vartheta_{obs} - \Delta\vartheta_{solvent}) = \frac{1}{\tau_m} * \frac{\left(T_{2m}^{-1} + T_{2m}^{-1} * \tau_m^{-1} + \Delta\omega_m^2\right)}{\left(T_{2m}^{-1} + \tau_m^{-1}\right)^2 + \Delta\omega_m^2} + \frac{1}{T_{2OS}} \qquad \textit{Eq. 6}$$

In Eq. 6, $\frac{1}{T_{2r}}$ is the reduced transverse relaxation rate constant, and P_m is the mole fraction of water in the coordination site compared to the bulk water. The observed and solvent $\Delta\vartheta$ represent the line widths of the water peak with and without the complex, respectively. The lifetime of the bound water molecule is represented as τ_m, T_{2m}^{-1} represents the transverse relaxation rate of the bound water, and $\Delta\omega_m$ is the difference between the resonance frequency of ^{17}O nuclei of solvent in the metal ion first coordination sphere and in the bulk. $\frac{1}{T_{2OS}}$ is the transverse relaxation that occurs from only outer sphere interactions with water.

At lower temperature ranges, Eq. 6 can be reduced to Eq. 7 due to T_{2m}^{-1} and $\frac{1}{T_{2OS}}$ being negligible.

$$\frac{1}{T_{2r}} = \frac{\pi}{P_m} * (\Delta\vartheta_{obs} - \nabla\vartheta_{solvent}) = \frac{1}{\tau_m} * \frac{\Delta\omega_m^2}{(\tau_m^{-2} + \Delta\omega_m^2)} \qquad \textit{Eq. 7}$$

Eq. 8 represents the logarithmic form of this data.

$$\ln\left(\frac{1}{T_{2r}}\right) = \ln\left\{\frac{1}{\tau_m} * \frac{\Delta\omega_m^2}{(\tau_m^{-2} + \Delta\omega_m^2)}\right\} \qquad \textit{Eq. 8}$$

Both the $\frac{1}{\tau_m}$ and $\Delta\omega_m$ terms can be further simplified as Eq. 9 and 10,

$$\frac{1}{\tau_m} = k_{ex} = \frac{k_b * T}{h}\, exp^{\left(\frac{\Delta S}{R} - \frac{\Delta H}{R*T}\right)} \qquad \textit{Eq. 9}$$

$$\Delta\omega_m = \frac{g_L \mu_b S(S+1)B}{T}\left(\frac{A}{\hbar}\right) \qquad \textit{Eq. 10}$$

Where k_{ex} is the exchange rate of the water in the inner sphere coordination site, k_b is Boltzmann's constant, h is Planck's constant, T is temperature, ΔS is the activation entropy and ΔH is the activation enthalpy. The Lande g-factor is represented as g_L, μ_b is the Bohr Magneton, S is the total spin state, B is the magnetic field, A is the hyperfine coupling constant and $\hbar$ is the reduced Planck's constant.

In Eq. 11, the Lande g-factor, magnetic moment, spin state, magnetic field, and hyperfine coupling constant terms are consolidated into a single parameter which is solved for in the treatment of the data. This consolidation is represented by the constant C:

$$\Delta\omega_m = \frac{C}{T} \qquad \textit{Eq. 11}$$

Since activation enthalpy and entropy cannot be directly measured, they are treated as parameters and solved for in the least squares fitting of $\ln\left(\frac{1}{T_{2r}}\right)$ vs. $\frac{1}{T}$. Uncertainty in the parameter values is obtained using a statistics report generated within the program.

T_1 Measurements for protons in complexes

Samples of 100 mM mononuclear complexes in D_2O were used for the inversion-recovery T_1 experiments at 25°C, where the standard two-pulse sequence (180° pulse followed by 90° pulse) was employed. The minimum expected T_1, the maximum expected T_1 ($\approx 5 \times T_1$), the delay times, and the total time of each experiment were varied and optimized for each proton considered. The ^{1}H NMR spectrum of each compound was divided into several regions to ensure effective inversion of the spin populations, as well as to avoid having off-axis magnetization and fold-overs when the observed pulse was applied. The baseline correction was performed for all spectra. To determine the value of T_1, the array of values of the parameter d2, which is between the minimum T_1 and the maximum T_1, was fit by an exponential curve as in equation 12.

$$M_t = (M(0) - M_0) * exp^{\left(\frac{-t}{T_1}\right)} + M_0 \qquad \textit{Eq. 12}$$

where M_0 is the equilibrium Z magnetization and $M(0)$ is the magnetization at time zero, which is immediately after the 180° pulse for an inversion recovery T_1 experiment. The T_1 values were obtained by averaging data from at least two independent experiments and recorded with the standard deviation.

T_2 Measurements for protons in complexes

^{1}H NMR spectra of solutions containing 10 mM complex were acquired on Bruker NEO 500 and processed by using MestReNova. The software was then used to calculate full width at half maximum (FWHM/linewidth) of prominent resonances (NMR > Processing > More Processing > Reference deconvolution), which were then multiplied by π to give $\frac{1}{T_2}$ (in Hz). A reciprocal was taken to generate T_2 (in sec). Note that for well-shimmed samples, $T_2 = T_2^*$ with T_2^* measured from linewidth at half-height (ϑ) in Equation 13.

$$\frac{1}{T_2} = \vartheta * \pi \quad \text{(Hz)} \qquad \textit{Eq. 13}$$

pH measurements and UV vis spectroscopy studies

pH measurements were taken with a Thermo Scientific 9110DJWP double-junction glass semimicro electrode connected to a 702 SM Titrino pH meter. UV–vis spectra were recorded on a Beckman–Coulter DU-800 spectrophotometer equipped with a Peltier temperature controller. Absorption spectra were collected over 200–800 nm at 37 °C.

For kinetic inertness studies, solutions containing ≥10 mM Co(II) complexes were prepared in 100 mM NaCl with 20 mM HEPES buffer, in the absence or presence of 1 equiv zinc (II) nitrate. The pH was adjusted to 7.4 using 1 M HCl or 1 M NaOH. Spectral changes were monitored over 4 h.

For $[Fe(L1)]^{+}$, solutions had 0.2 mM complex in 100 mM NaCl at pH 4. For $[Fe(L2)]^{2+}$, inertness studies were performed under conditions analogous to those used for the Co(II) complexes as reported.[2]

^{1}H NMR spectroscopy of paramagnetic metal complexes

Solutions of the metal complexes were prepared in D_2O at a final concentration of 10 mM in a total volume of 500 μL. The pD was adjusted to 7.8 for all cobalt complexes with small aliquots (~ 1 μL) of 0.10 M NaOD or DCl, as measured with a micro pH electrode, and the final volume was adjusted to 500 μL with D_2O prior to NMR analysis. The spectra were recorded at 25°C unless otherwise specified.

^{17}O NMR spectroscopy

Solutions were prepared with 10 mM complex, 0.10 M NaCl and 1% ^{17}O water. The pH of the solution was adjusted using small aliquots (~ 5 μL total) of 0.10 M NaOH or HCl. The solution was then transferred to an NMR tube, and a coaxial insert containing DMSO-d_6 was used to provide the field-frequency lock and for shimming prior to data acquisition.

ESI and High-Resolution mass spectrometry

A Thermo Fisher Linear Ion Trap Quadrupole mass spectrometer was employed to monitor the progress of reactions. High resolution mass spectrometry data was obtained on a Thermo Fisher Q-Exactive Focus Orbitrap (MS component) associated with a Dionex UltiMate 3000 (HPLC) to report the final mass-to-charge ratio of ligands and their complexes.

ICP-MS data

The iron concentration of each of the complexes was determined using a Thermo X-Series 2 ICP-MS. Samples were dissolved in 65–70% metal-free nitric acid for 3 days to undergo digestion. After the digestion, a cobalt internal standard was added, and the samples were diluted with Milli-Q water to contain 2% nitric acid and 50 ppb cobalt. A linear calibration curve ranging from 0.1 ppb to 100 ppb iron was also prepared and used to quantify the samples. Quantification and data analysis were performed using Thermo Fisher PlasmaLab.

Binding constants

^{1}H NMR spectroscopy titrations: Binding constants for KF interaction with the complexes were calculated by using the equation:

$$K_d = \frac{[M][L]}{[ML]} \qquad \textit{Eq. 14}$$

Where, K_d = dissociation constant (mM), $[M]$ = concentration of metal complex, $[L]$ = concentration of ligand (potassium fluoride or trifluorolactate) and $[ML]$ = concentration of bound metal-ligand species.

Concentrations of free complex and fluoride complexes were monitored by integration of proton resonances.

^{19}F NMR: Binding constants for KF interaction with the complexes were calculated by using the equation:

$$y = (y_0 + y_{max}) * \frac{(x+M+K_d) - \sqrt{(x+M+K_d)^2 - 4Mx}}{2x} \qquad \textit{Eq. 15}$$

Where, y = observed signal (chemical shift, Hz), y_0 = baseline correction (no binding), $y_{\max}$ = maximum change in chemical shift, x = ligand (titrant) concentration (mM), M = total metal complex concentration (mM), K_d = dissociation constant (mM).

A plot of chemical shift (δ) in (Hz) or linewidth in (Hz) versus concentration of the titrant can be fitted to the quadratic binding equation to obtain K_d values.

Parashift T_1-weighted phantom imaging

Imaging of compounds $[Co(L1)]^+$ and $[Co(L2)]^{2+}$ (10 mM in D2O, pH 7.4) was carried out at room temperature on a 7 Tesla preclinical MRI using a 23 mm, quadrature RF coil (ParaVision 360, Bruker Biospin). The coil was affixed to manufacturer-supplied imaging sled (ManPac) to allow for precise removal and re-insertion of the samples and RF coil. To serve as a reference to parashift images, 100% deuterated water was spiked with ferumoxytol (AMAG Pharmaceuticals) dissolved in ^{1}H distilled water to achieve a concentration of 0.5% ^1H_2O and 15 µg/mL Fe, which provided comparable proton density and T_2 relaxation times to the parashift samples. A phantom consisting of 10 mM $CuSO_4$ was temporarily inserted between parashift and ferumoxytol samples to provide sufficient ^{1}H signal for completion of pre-scan adjustments (global shims, Larmor frequency, RF excitation power). The B_0 field was shimmed to a voxel of 25^3 mm size, after which the $CuSO_4$ phantom was removed with minimal disturbance to parashift samples.

Water-suppressed, non-localized one-dimensional spectra were acquired to identify parashift resonances and verify correspondence with previously obtained high-resolution NMR spectra. Spectroscopic parameters included a spectral width of 195 ppm, 4096 acq. points, repetition time (TR) of 700 ms, and 200 signal averages, with RF excitation centered on the water resonance.

Parashift images were acquired with RF excitation pulses centered at multiple ppm offsets and spectral bandwidths (Table S1) using a single-slice, spoiled gradient echo scan with the following common parameters: FOV = 64 mm, matrix size = 96 x 96, slice thickness = 3 mm, flip angle = 90°, NEX = 500. Echo and repetition times were adjusted slightly to accommodate variable frequency encoding bandwidths as noted in Table S1.

Reconstructed images were normalized such that the standard deviation of background noise (measured in air regions) was set to unity. Pseudo-color visualization was applied to normalized datasets within MATLAB (MathWorks) to enhance visual clarity of signal differences.

Table S1: Acquisition parameters for parashift imaging.

Ligand	Central ppm	Encoding Bandwidth (ppm)	Echo Time (ms)	Repetition Time (ms)
**$[Co(L1)]^{+}$**	+4.7	50	2.5	7.5
**$[Co(L1)]^{+}$**	-55	50	2.5	7.5
**$[Co(L2)]^{2+}$**	+4.7	33.3	3.0	9.2
**$[Co(L2)]^{2+}$**	-27	33.3	3.0	9.2
**$[Co(L2)]^{2+}$**	+77	33.3	3.0	9.2

X-ray Diffraction data

Table S 2: Crystal data and structure refinement for $[Co(L2)(NO_3)](NO_3)$. CCDC Deposition Number 2545340

**Empirical formula**	$C_{28.5}H_{41}CoN_7O_{7.5}$
**Formula weight**	660.61
**Temperature/K**	100.15
**Crystal system**	monoclinic
**Space group**	$P2_1/n$
**a/Å**	10.9265(3)
**b/Å**	20.1552(6)
**c/Å**	14.0544(5)
**α/°**	90
**β/°**	94.772(3)
**γ/°**	90
**Volume/Å^3**	3084.41(17)
**Z**	4
**ρ_{calc}g/cm^3**	1.423
**μ/mm^{-1}**	0.328
**F(000)**	1392.0
**Crystal size/mm^3**	0.18 × 0.14 × 0.07
**Radiation**	Ag Kα (λ = 0.56087)
**2Θ range for data collection/°**	4.59 to 41.584
**Index ranges**	$-13 \leq h \leq 13$, $-25 \leq k \leq 25$, $-17 \leq l \leq 17$
**Reflections collected**	35342
**Independent reflections**	6536 [R_{int} = 0.0410, R_{sigma} = 0.0264]
**Data/restraints/parameters**	6536/228/522
**Goodness-of-fit on F^2**	1.064
**Final R indexes [I>=2σ (I)]**	R_1 = 0.0432, wR_2 = 0.1145
**Final R indexes [all data]**	R_1 = 0.0516, wR_2 = 0.1188
**Largest diff. peak/hole / e Å^{-3}**	0.81/-0.38

Table S 3: Bond lengths for [Co(L2)(NO_3)](NO_3).

Atom1	Atom2	Length (Å)	Atom1	Atom2	Length (Å)
**Co1**	O1	2.040(2)	C11	H11	0.95
**Co1**	N1	2.225(2)	C11	C12	1.378(4)
**Co1**	N2	2.153(2)	C11	C10	1.383(3)
**Co1**	N4	2.240(2)	C2	H2A	0.99
**Co1**	N5A	2.19(2)	C2	H2B	0.989
**Co1**	N3	2.152(2)	C20	H20A	0.98
**O1**	N6A	1.295(6)	C20	H20B	0.98
**N1**	C7	1.492(3)	C20	H20C	0.98
**N1**	C1	1.498(3)	C12	H12	0.95
**N1**	C6	1.483(3)	C10	H10	0.95
**N2**	C2	1.483(3)	C22A	C21A	1.47(2)
**N2**	C3	1.490(4)	C22A	C23A	1.39(2)
**N2**	C14	1.478(3)	C15	C14	1.506(4)
**N4**	C19	1.350(3)	C15	C16	1.379(3)
**N4**	C15	1.347(3)	C3	H3A	0.99
**N5A**	C22A	1.37(2)	C3	H3B	0.99
**N5A**	C26A	1.36(2)	C3	C4	1.520(4)
**N3**	C4	1.480(3)	C4	H4A	0.99
**N3**	C21A	1.47(1)	C4	H4B	0.99
**N3**	C5	1.498(3)	C14	H14A	0.99
**N6A**	O2A	1.221(8)	C14	H14B	0.99
**N6A**	O3A	1.202(7)	C21A	H21A	0.99
**C8**	C7	1.512(3)	C21A	H21B	0.99
**C8**	C13	1.393(3)	C26A	C27A	1.50(2)
**C8**	C9	1.393(3)	C26A	C25A	1.39(1)
**C7**	H7A	0.99	C16	H16	0.95
**C7**	H7B	0.99	C5	H5A	0.99
**C1**	H1A	0.99	C5	H5B	0.99
**C1**	H1B	0.99	C23A	H23A	0.95
**C1**	C2	1.518(3)	C23A	C24A	1.37(2)
**C13**	H13	0.95	C27A	H27A	0.98
**C13**	C12	1.383(4)	C27A	H27B	0.98
**C9**	H9	0.95	C27A	H27C	0.98
**C9**	C10	1.391(3)	C24A	H24A	0.95
**C6**	H6A	0.99	C24A	C25A	1.37(2)
**C6**	H6B	0.99	C25A	H25A	0.95
**C6**	C5	1.526(3)	N7	O6A	1.20(1)
**C19**	C18	1.402(4)	N7	O4A	1.215(8)
**C19**	C20	1.493(4)	N7	O5A	1.326(6)
**C18**	H18	0.95	O7	H7	0.839
**C18**	C17	1.367(4)	O7	C28	1.395(5)
**C17**	H17	0.95	C28	H28A	0.98
**C17**	C16	1.385(4)	C28	H28B	0.98

Table S 4: Bond angles for [Co(L2)(NO_3)](NO_3).

Atom1	Atom2	Atom3	Angle (°)	Atom1	Atom2	Atom3	Angle (°)
**O1**	Co1	N1	87.78(6)	N2	C2	H2B	109.6
**O1**	Co1	N2	92.27(7)	C1	C2	H2A	109.5
**O1**	Co1	N4	92.54(7)	C1	C2	H2B	109.6
**O1**	Co1	N5A	111.2(5)	H2A	C2	H2B	108.1
**O1**	Co1	N3	167.89(7)	C19	C20	H20A	109.5
**N1**	Co1	N2	80.99(7)	C19	C20	H20B	109.5
**N1**	Co1	N4	159.97(7)	C19	C20	H20C	109.5
**N1**	Co1	N5A	94.0(5)	H20A	C20	H20B	109.4
**N1**	Co1	N3	81.76(7)	H20A	C20	H20C	109.5
**N2**	Co1	N4	78.98(7)	H20B	C20	H20C	109.5
**N2**	Co1	N5A	155.8(5)	C13	C12	C11	120.5(2)
**N2**	Co1	N3	80.11(8)	C13	C12	H12	119.8
**N4**	Co1	N5A	104.4(5)	C11	C12	H12	119.8
**N4**	Co1	N3	95.19(7)	C9	C10	C11	120.0(2)
**N5A**	Co1	N3	75.8(5)	C9	C10	H10	120
**Co1**	O1	N6A	123.2(3)	C11	C10	H10	120
**Co1**	N1	C7	112.7(1)	N5A	C22A	C21A	118(1)
**Co1**	N1	C1	108.2(1)	N5A	C22A	C23A	121(1)
**Co1**	N1	C6	103.0(1)	C21A	C22A	C23A	121(1)
**C7**	N1	C1	111.4(2)	N4	C15	C14	116.2(2)
**C7**	N1	C6	110.5(2)	N4	C15	C16	123.1(2)
**C1**	N1	C6	110.7(2)	C14	C15	C16	120.6(2)
**Co1**	N2	C2	104.6(1)	N2	C3	H3A	109.4
**Co1**	N2	C3	111.5(2)	N2	C3	H3B	109.4
**Co1**	N2	C14	104.3(2)	N2	C3	C4	111.4(2)
**C2**	N2	C3	112.6(2)	H3A	C3	H3B	108
**C2**	N2	C14	111.5(2)	H3A	C3	C4	109.3
**C3**	N2	C14	111.8(2)	H3B	C3	C4	109.3
**Co1**	N4	C19	131.8(2)	N3	C4	C3	109.7(2)
**Co1**	N4	C15	109.6(1)	N3	C4	H4A	109.7
**C19**	N4	C15	118.2(2)	N3	C4	H4B	109.7
**Co1**	N5A	C22A	110(1)	C3	C4	H4A	109.7
**Co1**	N5A	C26A	130(1)	C3	C4	H4B	109.7
**C22A**	N5A	C26A	119(2)	H4A	C4	H4B	108.2
**Co1**	N3	C4	106.5(1)	N2	C14	C15	110.4(2)
**Co1**	N3	C21A	107.2(6)	N2	C14	H14A	109.6
**Co1**	N3	C5	110.1(1)	N2	C14	H14B	109.6
**C4**	N3	C21A	105.9(6)	C15	C14	H14A	109.6
**C4**	N3	C5	111.3(2)	C15	C14	H14B	109.5
**C21A**	N3	C5	115.4(6)	H14A	C14	H14B	108.1
**O1**	N6A	O2A	115.3(5)	N3	C21A	C22A	104(1)
**O1**	N6A	O3A	124.0(5)	N3	C21A	H21A	111
**O2A**	N6A	O3A	120.7(6)	N3	C21A	H21B	111

**C7**	C8	C13	121.3(2)	C22A	C21A	H21A	111
**C7**	C8	C9	120.2(2)	C22A	C21A	H21B	111
**C13**	C8	C9	118.4(2)	H21A	C21A	H21B	109
**N1**	C7	C8	115.6(2)	N5A	C26A	C27A	120(1)
**N1**	C7	H7A	108.4	N5A	C26A	C25A	121(1)
**N1**	C7	H7B	108.4	C27A	C26A	C25A	119(1)
**C8**	C7	H7A	108.4	C17	C16	C15	118.7(2)
**C8**	C7	H7B	108.4	C17	C16	H16	120.6
**H7A**	C7	H7B	107.4	C15	C16	H16	120.7
**N1**	C1	H1A	109.3	N3	C5	C6	112.9(2)
**N1**	C1	H1B	109.3	N3	C5	H5A	109
**N1**	C1	C2	111.7(2)	N3	C5	H5B	109
**H1A**	C1	H1B	107.9	C6	C5	H5A	109
**H1A**	C1	C2	109.2	C6	C5	H5B	109
**H1B**	C1	C2	109.3	H5A	C5	H5B	107.8
**C8**	C13	H13	119.7	C22A	C23A	H23A	120
**C8**	C13	C12	120.7(2)	C22A	C23A	C24A	120(1)
**H13**	C13	C12	119.7	H23A	C23A	C24A	120
**C8**	C9	H9	119.7	C26A	C27A	H27A	109
**C8**	C9	C10	120.6(2)	C26A	C27A	H27B	109
**H9**	C9	C10	119.7	C26A	C27A	H27C	109
**N1**	C6	H6A	109.3	H27A	C27A	H27B	109
**N1**	C6	H6B	109.2	H27A	C27A	H27C	109
**N1**	C6	C5	111.8(2)	H27B	C27A	H27C	110
**H6A**	C6	H6B	107.9	C23A	C24A	H24A	120
**H6A**	C6	C5	109.3	C23A	C24A	C25A	119(1)
**H6B**	C6	C5	109.3	H24A	C24A	C25A	120
**N4**	C19	C18	120.8(2)	C26A	C25A	C24A	120(1)
**N4**	C19	C20	119.0(2)	C26A	C25A	H25A	120
**C18**	C19	C20	120.2(2)	C24A	C25A	H25A	120
**C19**	C18	H18	119.9	O6A	N7	O4A	125.1(7)
**C19**	C18	C17	120.3(2)	O6A	N7	O5A	116.6(6)
**H18**	C18	C17	119.9	O4A	N7	O5A	117.7(5)
**C18**	C17	H17	120.6	H7	O7	C28	109.5
**C18**	C17	C16	118.7(2)	O7	C28	H28A	109.5
**H17**	C17	C16	120.6	O7	C28	H28B	109.4
**H11**	C11	C12	120.2	O7	C28	H28C	109.5
**H11**	C11	C10	120.1	H28A	C28	H28B	109.5
**C12**	C11	C10	119.7(2)	H28A	C28	H28C	109.5
**N2**	C2	C1	110.4(2)	H28B	C28	H28C	109.5
**N2**	C2	H2A	109.5				

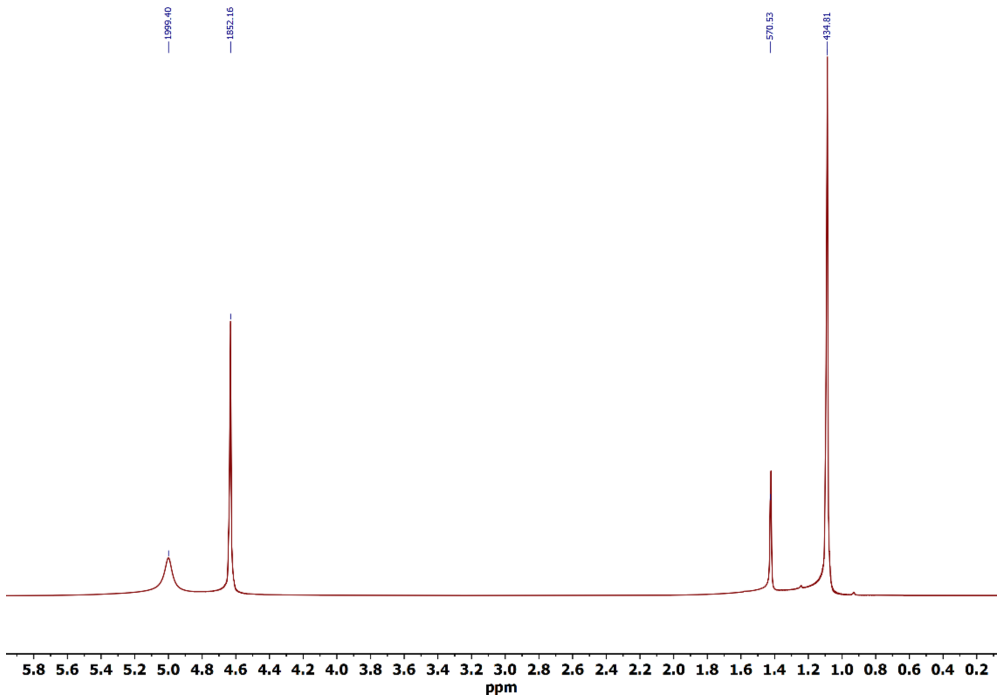

Figure S 1: ¹H NMR experiment used to calculate magnetic susceptibility for 10 mM $[Fe(L1)]^+$ in 5% t-butanol in D_2O.

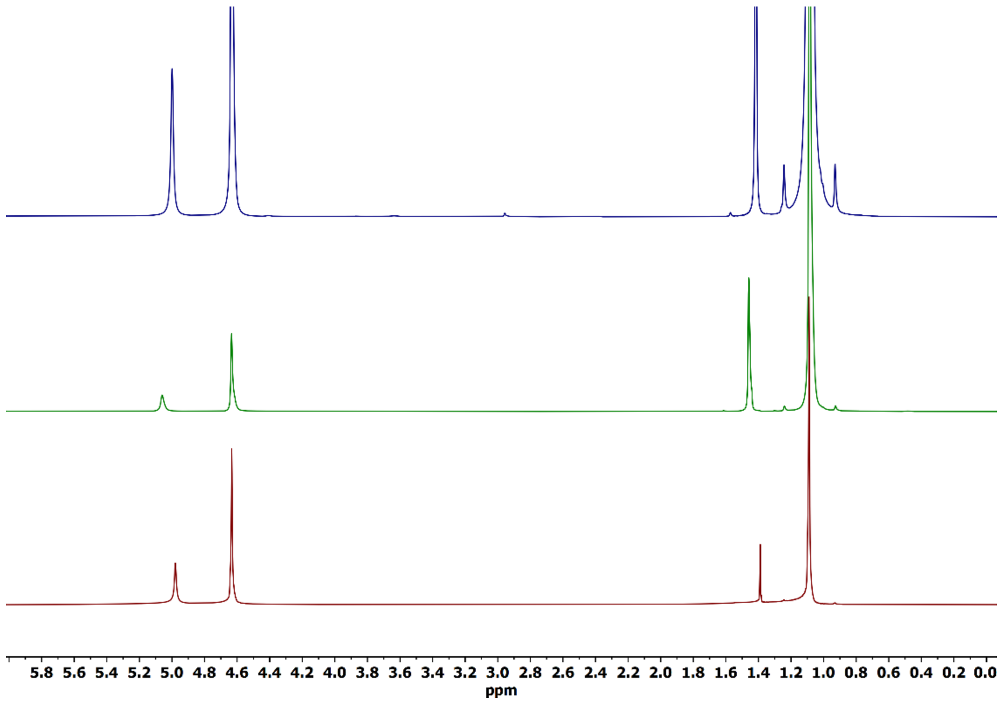

Figure S 2: ¹H NMR experiment used to calculate magnetic susceptibility for 10 mM $[Co(L1)]^+$ in 5% t-butanol in D_2O. With counteranions (A) chloride, (B) perchlorate and (C) nitrate.

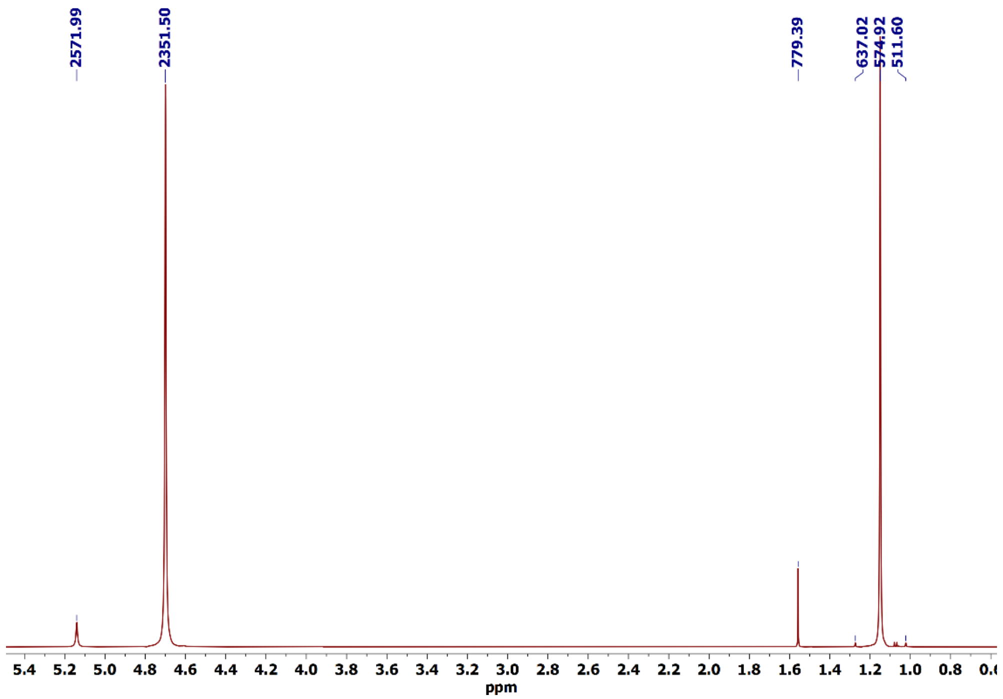

Figure S 3: ^{1}H NMR experiment used to calculate magnetic susceptibility for 10 mM $[Co(L2)]^{2+}$ in 5% t-butanol in D_2O.

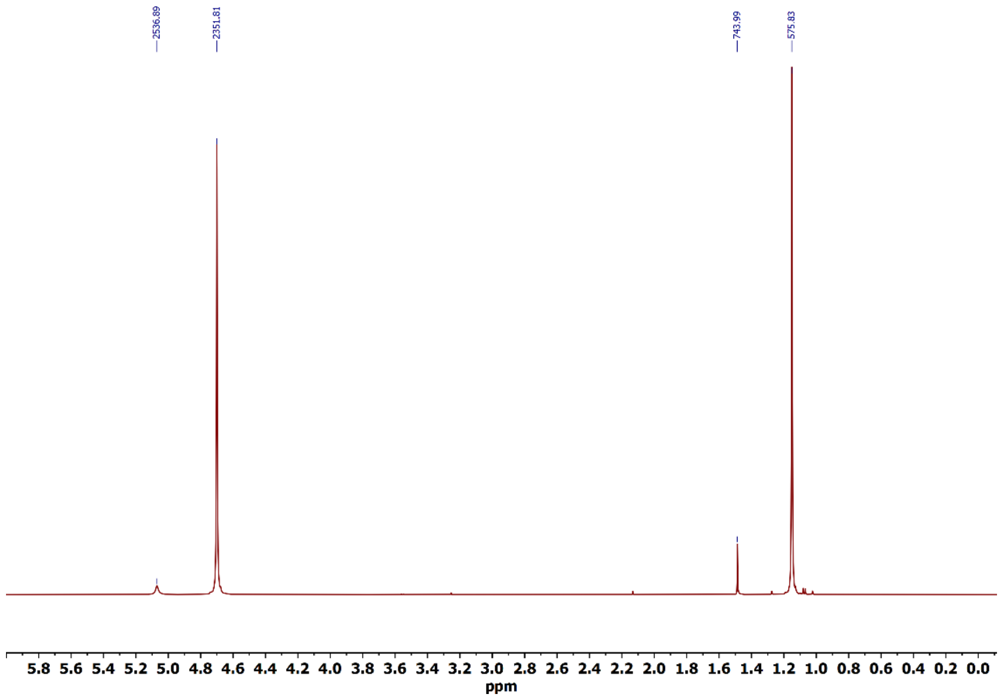

Figure S 4: ^{1}H NMR experiment used to calculate magnetic susceptibility for 10 mM $[Co(L3)]^{2+}$ in 5% t-butanol in D_2O.

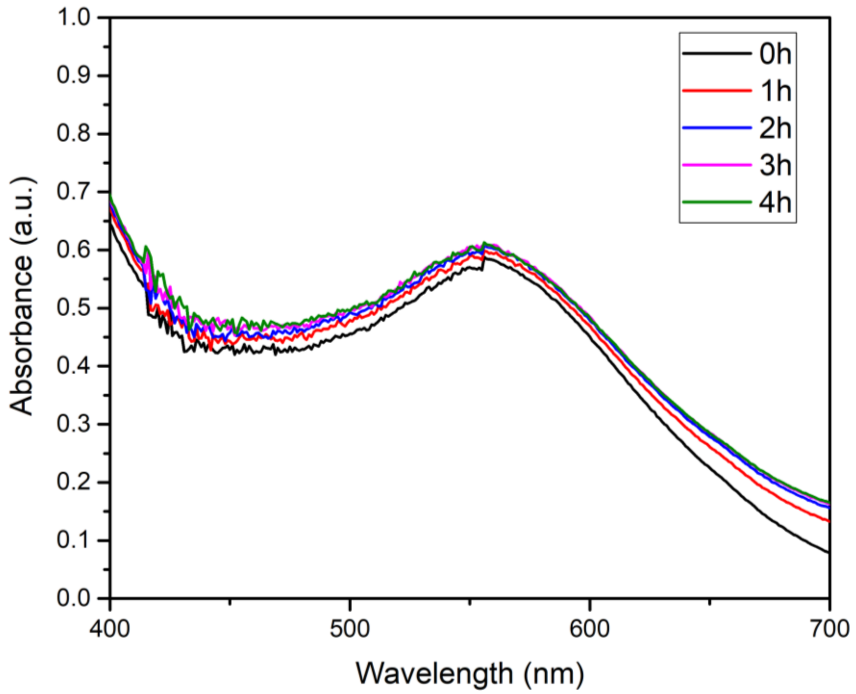

Figure S 5: UV-Vis spectra of $[Co(L1)]^{+}$ in the presence of HEPES buffer over 4 hours. Conditions: 20 mM complex, 20 mM HEPES, 0.1 M NaCl, pH 7.4, 37 °C.

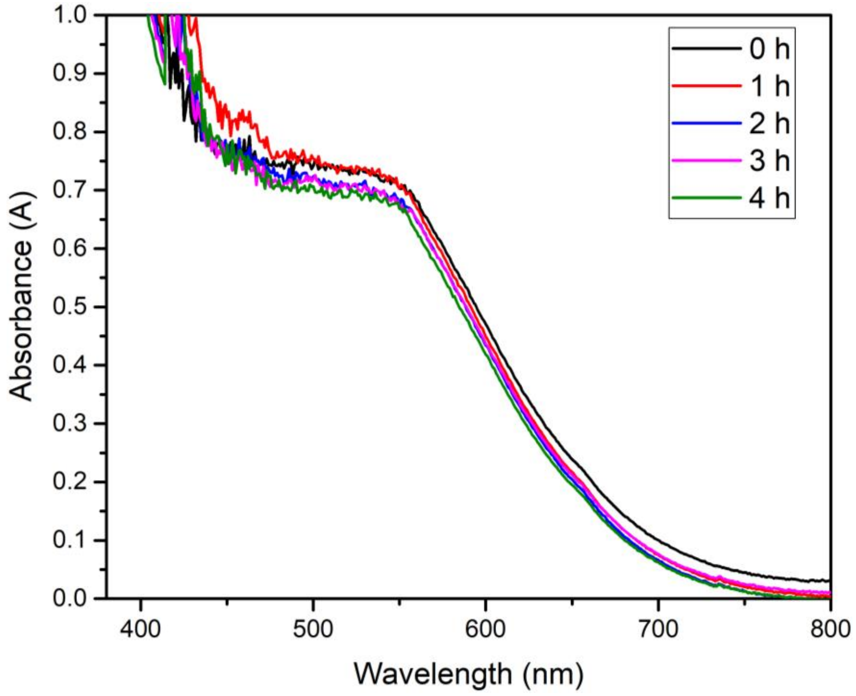

Figure S 6: UV-Vis spectra of $[Co(L1)]^{+}$ in the presence of Zn^{2+} over 4 hours. Conditions: 20 mM complex, 20 mM HEPES, 0.1 M NaCl, 20 mM $Zn(NO_3)_2 . 6H_2O$, pH 7.4, 37 °C.

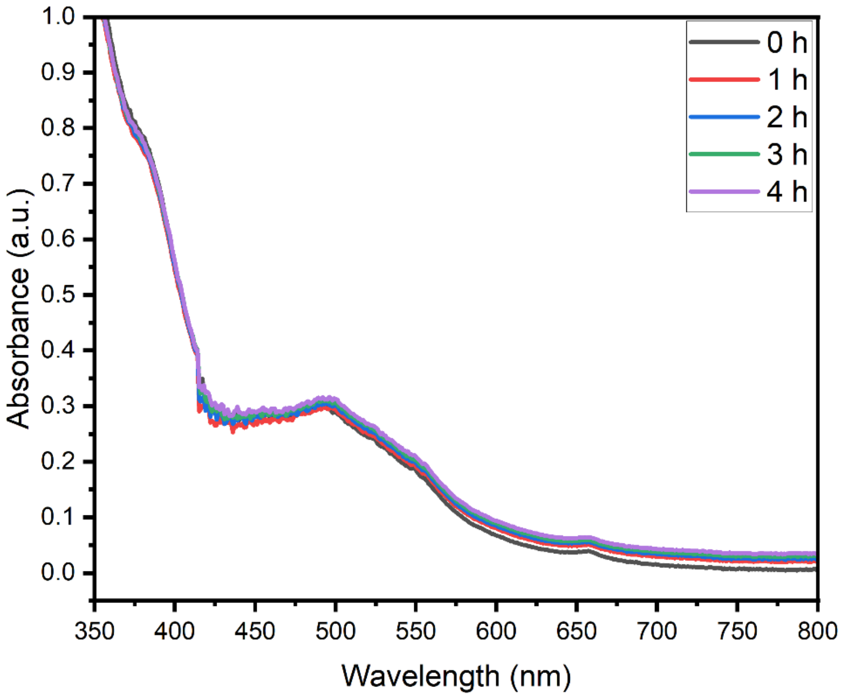

Figure S 7: UV-Vis spectra of $[Co(L2)]^{2+}$ in the presence of HEPES buffer over 4 hours. Conditions: 10 mM complex, 20 mM HEPES, 0.1 M NaCl, pH 7.4, 37 °C.

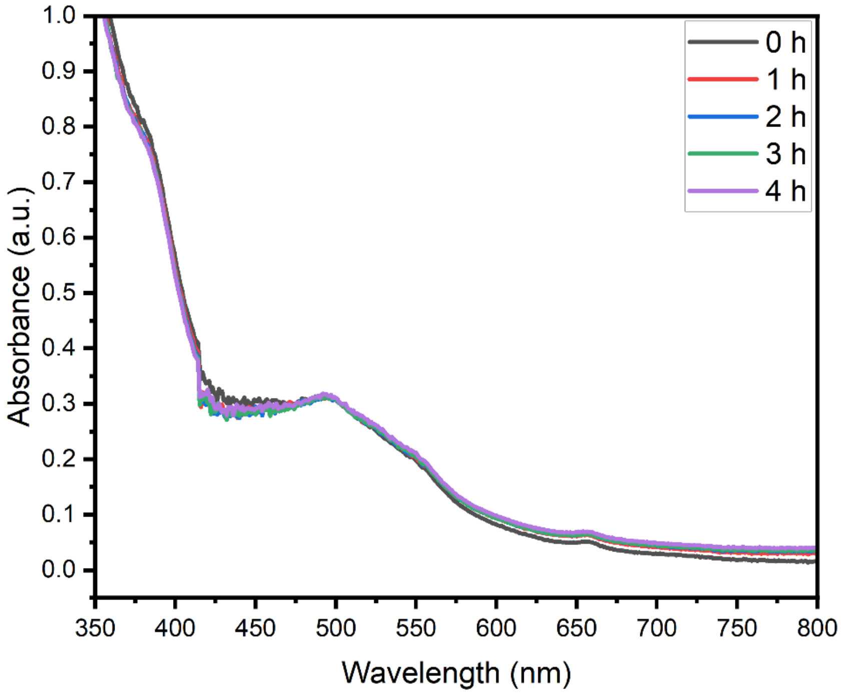

Figure S 8: UV-Vis spectra of $[Co(L2)]^{2+}$ in the presence of Zn^{2+} over 4 hours. Conditions: 10 mM complex, 20 mM HEPES, 0.1 M NaCl, 10 mM $Zn(NO_3)_2$, pH 7.4, 37 °C.

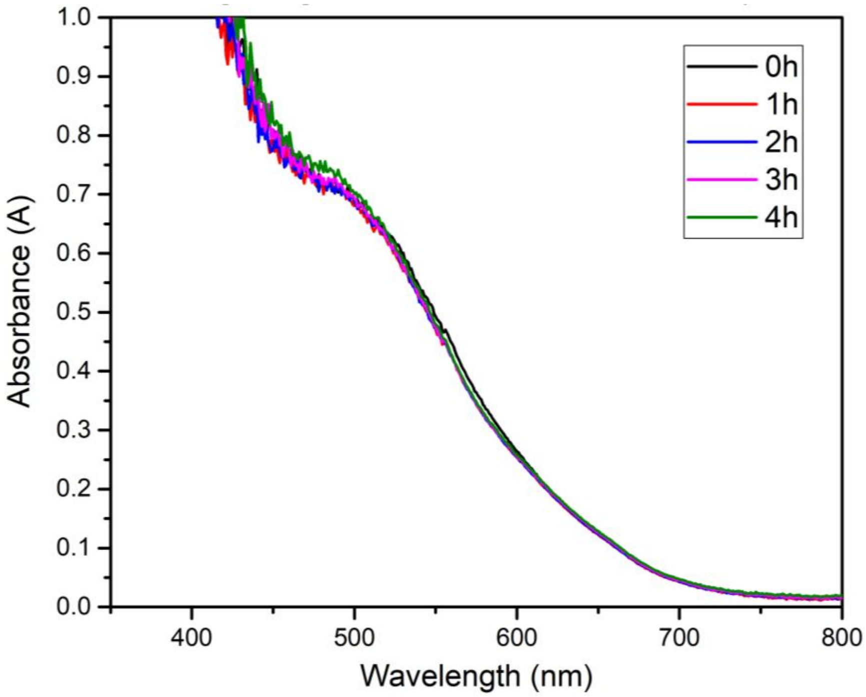

Figure S 9: UV-Vis spectra of $[Co(L3)]^{2+}$ in the presence of HEPES buffer over 4 hours. Conditions: 10 mM complex, 20 mM HEPES, 0.1 M NaCl, pH 7.4, 37° C.

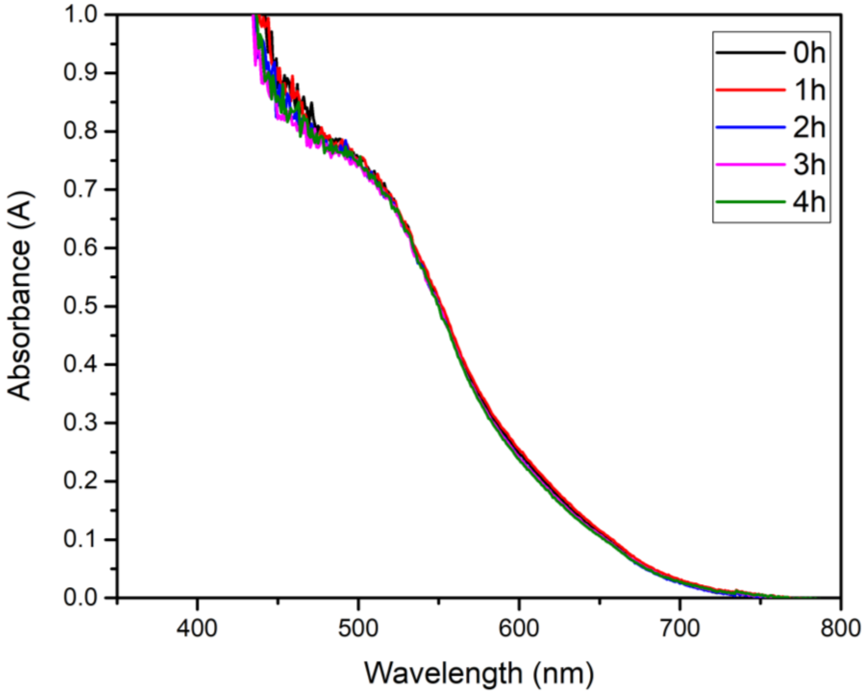

Figure S 10: UV-Vis spectra of $[Co(L3)]^{2+}$ in the presence of Zn^{2+} over 4 hours. Conditions: 10 mM complex, 20 mM HEPES, 0.1 M NaCl, 10 mM $Zn(NO_3)_2$, pH 7.4, 37 °C.

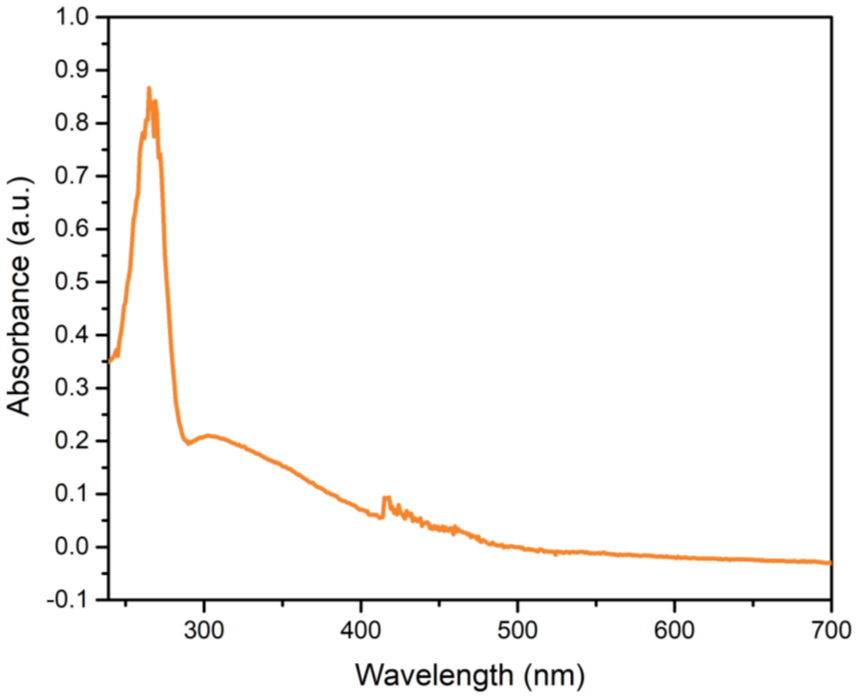

Figure S 11: UV-Vis spectrum of $[Fe(L1)]^+$. Conditions: 0.20 mM complex, 0.1 M NaCl, pH 4.0, 37 °C.

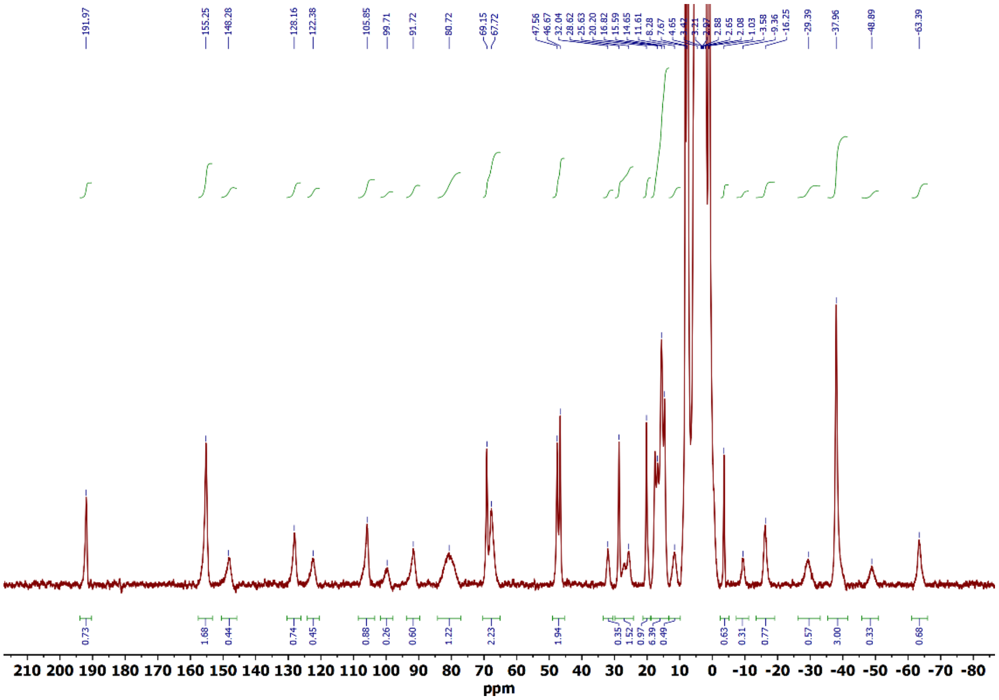

Figure S 12: ^1H NMR spectrum of $[Fe(L1)]^+$ in D_2O. Conditions: 10 mM, pD 4.4

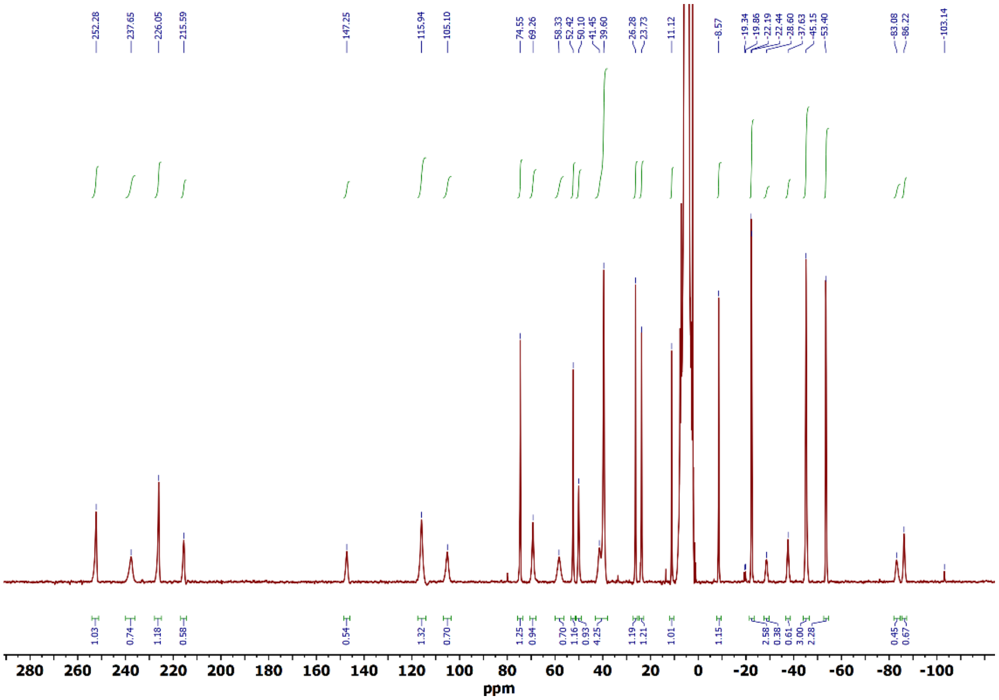

Figure S 13: ^{1}H NMR spectrum of $[Co(L1)]^{+}$ in D_2O. Conditions: 10 mM, pD 7.8

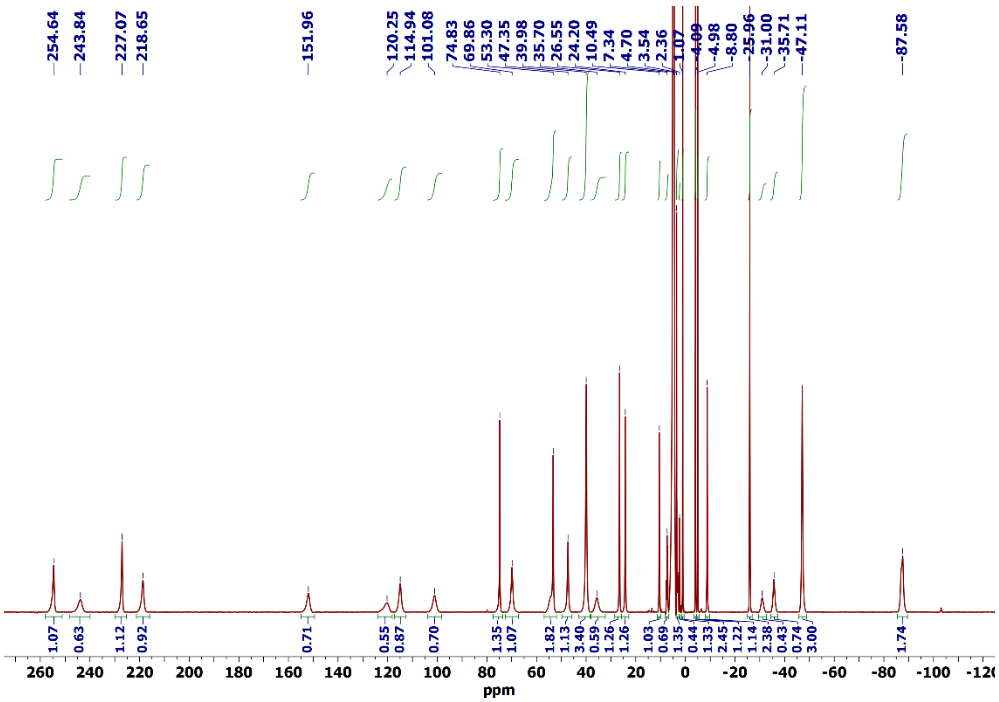

Figure S 14: ^{1}H NMR spectrum of $[Co(L2)]^{2+}$ in D_2O. Conditions: 10 mM, pD 7.8

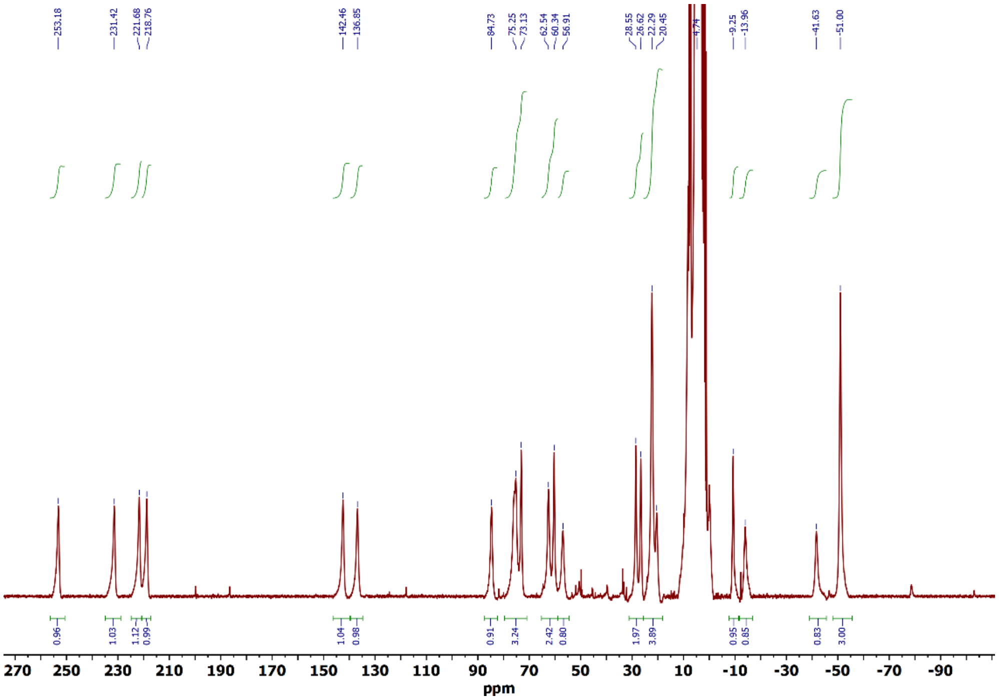

Figure S 15: ^{1}H NMR spectrum of $[Co(L3)]^{2+}$ in D_2O. Conditions: 10 mM, pD 7.8

Table S5: Assignment of proton resonances of $[Co(L1)]^{+}$ based on R_1, R_2 and integration.
Conditions: 100 mM complex in 20 mM HEPES, 0.1M NaCl, D_2O, pD 7.8, 25 °C

Chemical shift (ppm)	Linewidth FWHM (Hz)	R_1 (s^{-1})	R_2 (s^{-1})	Integration	Type of proton
**74.6**	107	100	340	1.3	Pyridine
**39.6**	225.2	460	710	2.8	Methyl
**26.3**	85.1	330	270	1.2	Pyridine
**-45.1**	206.7	370	650	3	Methyl
**-53.4**	186.3	83	590	2.3	Methylene

Table S6: Assignment of proton resonances of $[Co(L2)]^{2+}$ based on R_1, R_2 and integration
Conditions: 100 mM complex in 20 mM HEPES, 0.1M NaCl, D_2O, pD 7.8, 25 °C

Chemical shift (ppm)	Linewidth FWHM (Hz)	R_1 (s^{-1})	R_2 (s^{-1})	Integration	Type of proton
**74.8**	95.8	65	300	1.4	Pyridine
**40.0**	228.9	460	720	3.4	Methyl
**26.6**	74.9	24	240	1.3	Pyridine
**24.2**	91.7	56	290	1.3	Pyridine
**-26.0**	51.0	43	160	2.4	Methylene
**-47.1**	188.9	400	590	3.0	Methyl

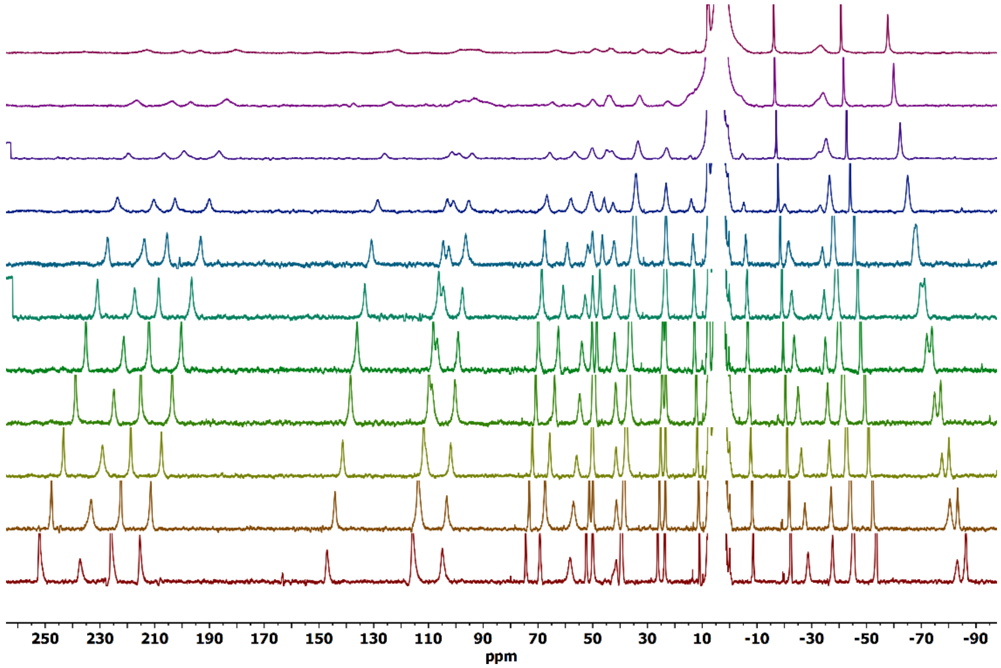

Figure S 16: Variable temperature paramagnetic ^{1}H NMR spectra of $[Co(L1)]^+$. Bottom (25 °C) to top (75 °C) with 5°C interval. Conditions: 10 mM, D_2O, pD 7.8.

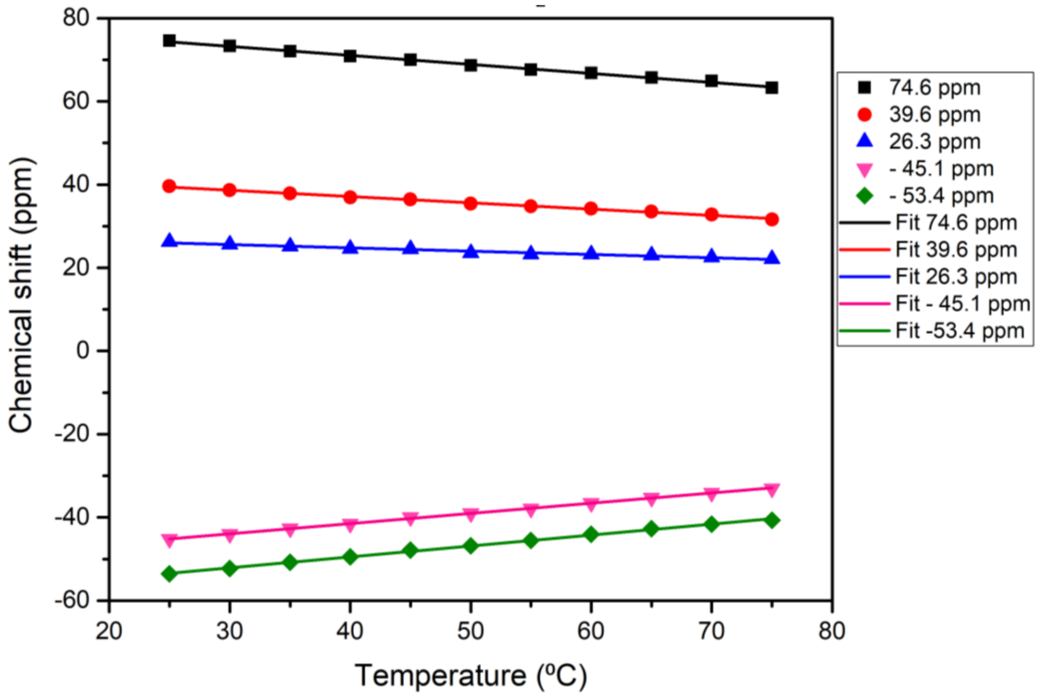

Figure S 17: Temperature dependence of ^{1}H chemical shifts of $[Co(L1)]^+$. Conditions: 10 mM, D_2O, pD 7.8.

Table S7: Slopes from temperature dependence of ^{1}H chemical shifts of $[Co(L1)]^+$. Conditions: 10 mM, D_2O, pD 7.8.

**Chemical shift (ppm)**	**74.6**	**39.6**	**26.3**	**-45.1**	**-53.4**
**Temperature coefficient (ppm/°C)**	0.22	0.15	0.08	0.25	0.26

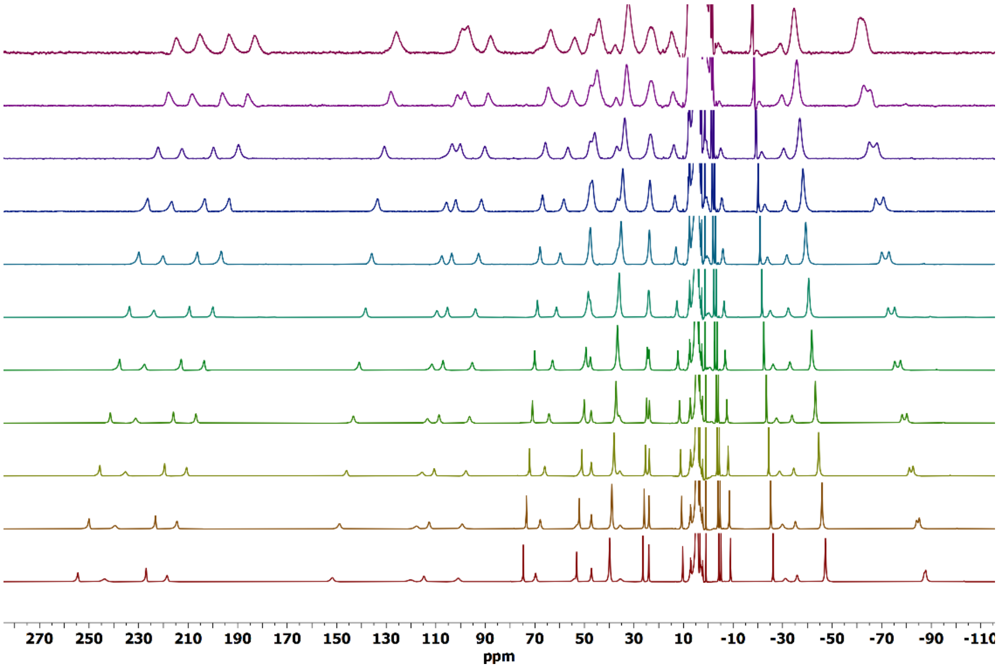

Figure S 18: Variable temperature paramagnetic ^{1}H NMR spectra of $[Co(L2)]^{2+}$. Bottom (25 °C) to top (75 °C) with 5°C interval. Conditions: 10 mM, D_2O, pD 7.8.

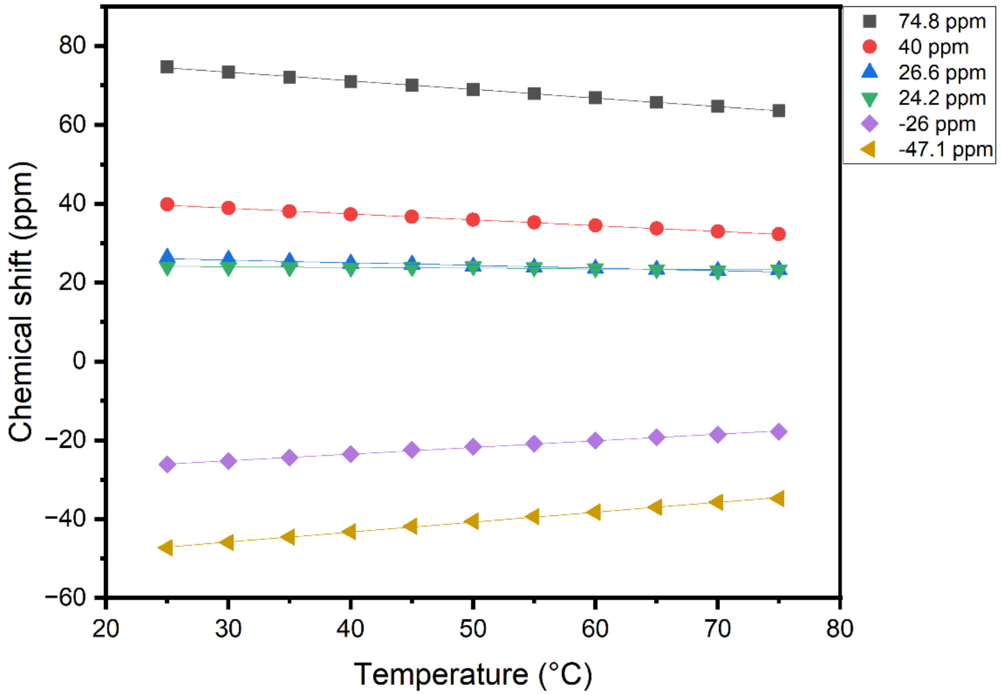

Figure S 19: Temperature dependence of ^1H chemical shifts of $[Co(L2)]^{2+}$. Conditions: 10 mM, D_2O, pD 7.8.

Table S8: Temperature dependence of ^1H chemical shifts of $[Co(L2)]^{2+}$. Conditions: 10 mM, D_2O, pD 7.8.

**Chemical shift (ppm)**	**74.8**	**40.0**	**26.6**	**24.2**	**-26**	**-47.1**
**Temperature coefficient (ppm/°C)**	0.22	0.15	0.07	0.02	0.17	0.25

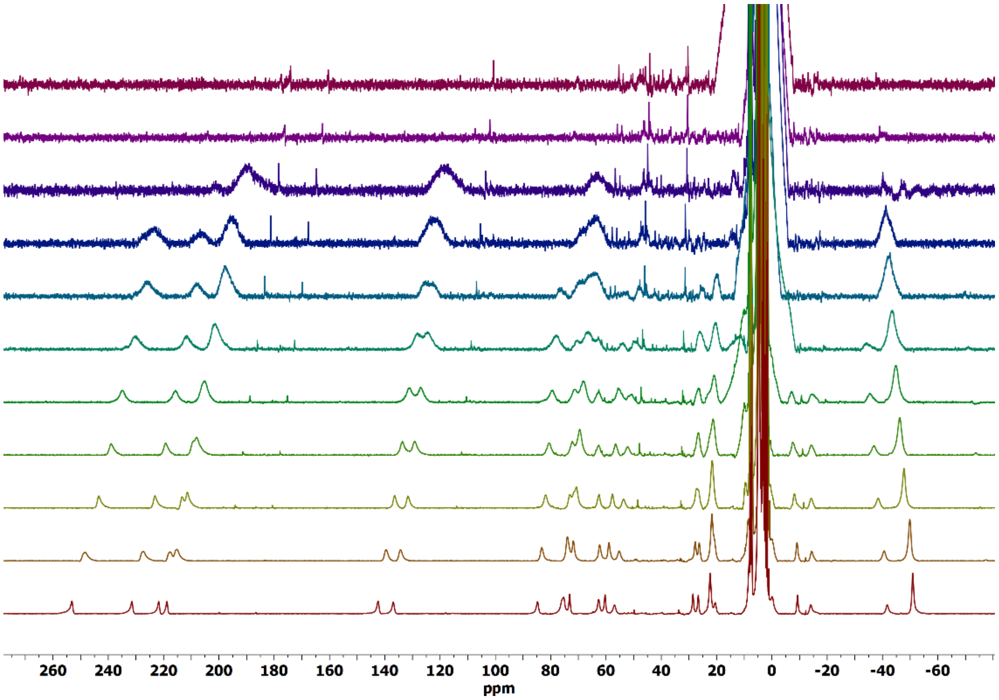

Figure S 20: Variable temperature paramagnetic ^{1}H NMR spectra of $[Co(L3)]^{2+}$. Bottom (25 °C) to top (75 °C) with 5°C interval. Conditions: 10 mM, D_2O, pD 7.8.

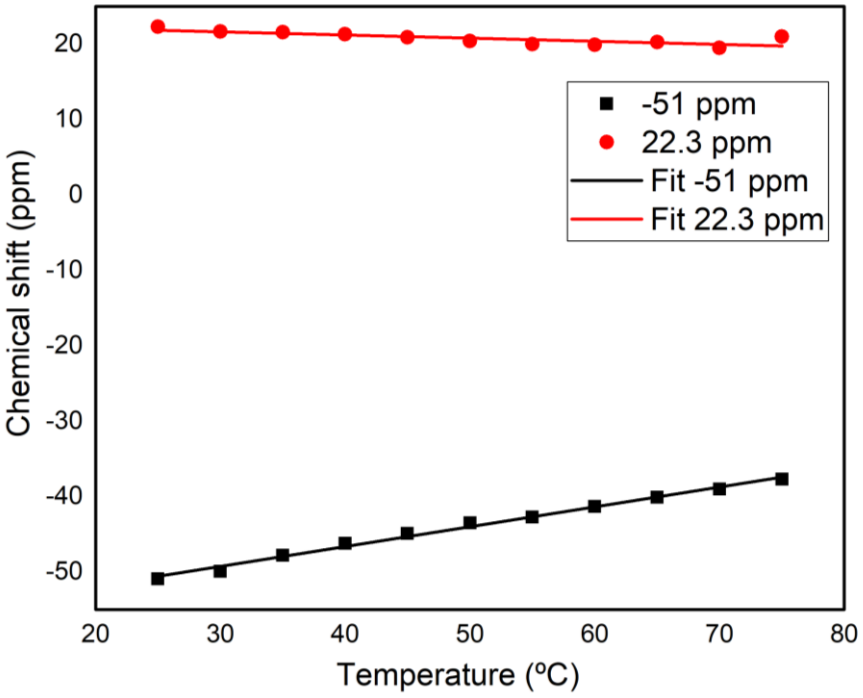

Figure S 21: Temperature dependence of selected ^{1}H chemical shifts of $[Co(L3)]^{2+}$. Conditions: 10 mM, D_2O, pD 7.8.

Table S9: Temperature dependence of ^1H chemical shifts of $[Co(L3)]^{2+}$. Conditions: 10 mM, D_2O, pD 7.8.

**Chemical shift (ppm)**	**22.3**	**-51.0**
**Temperature coefficient (ppm/°C)**	0.04	0.26

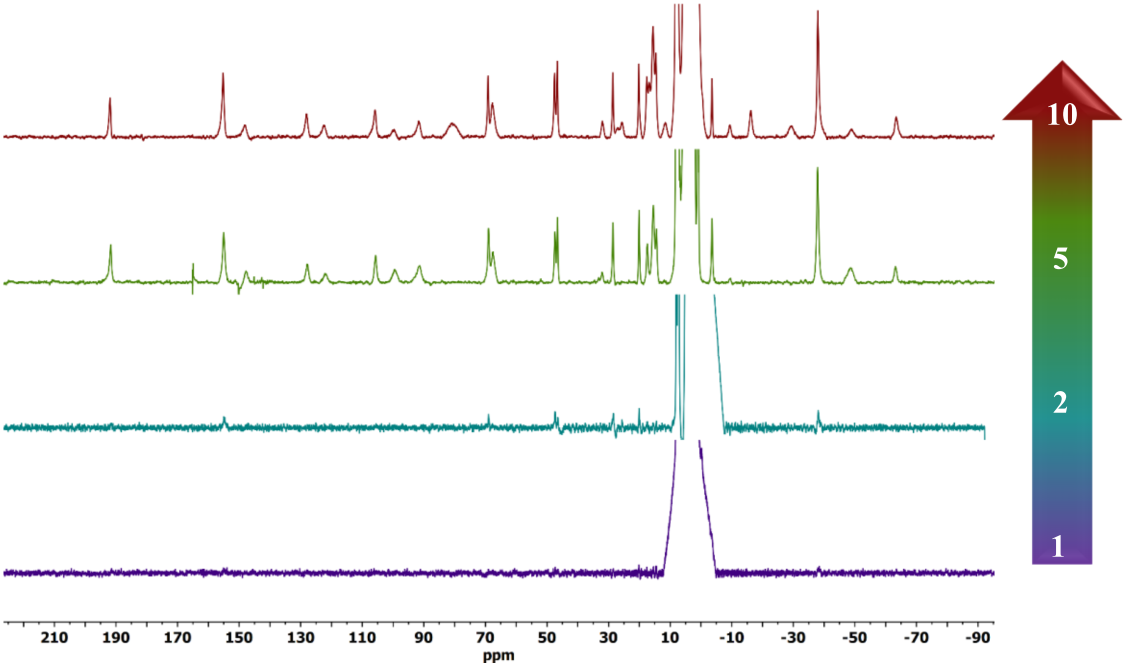

Figure S 22: ^1H NMR spectra to study detection limit of $[Fe(L1)]^+$. Conditions: 1 mM - 10 mM complex, pD 4.4, D_2O.

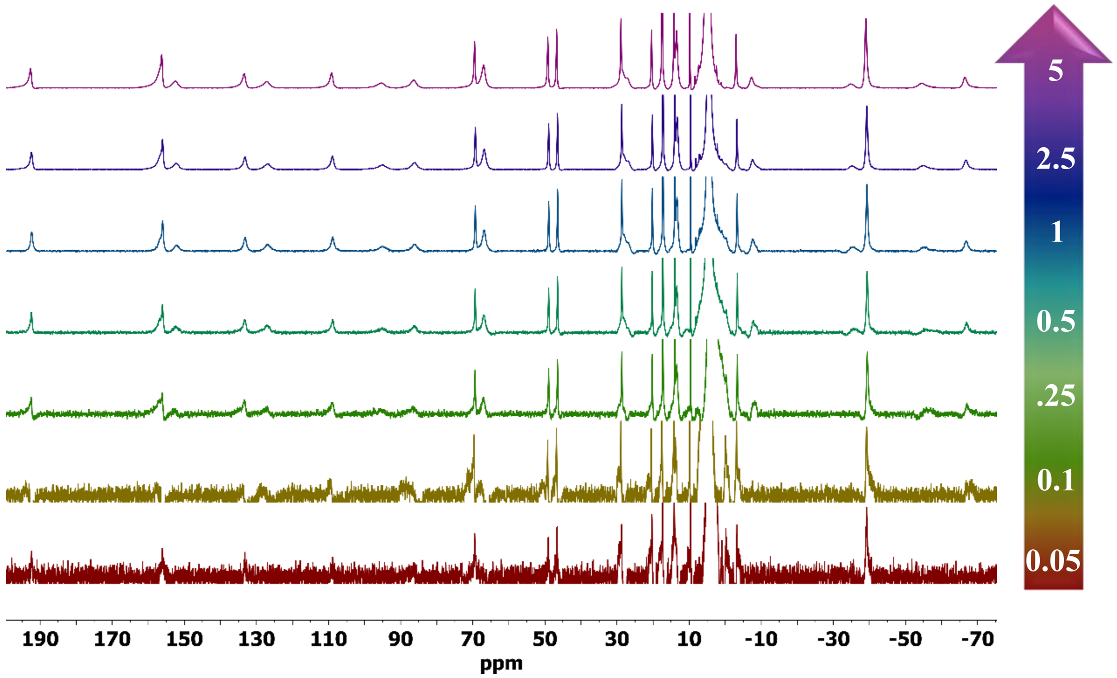

Figure S 23: ^{1}H NMR spectra to study limit of detection of $[Fe(L2)]^{2+}$. Conditions: 0.05 mM - 5 mM complex, pD 7.8, D_2O.

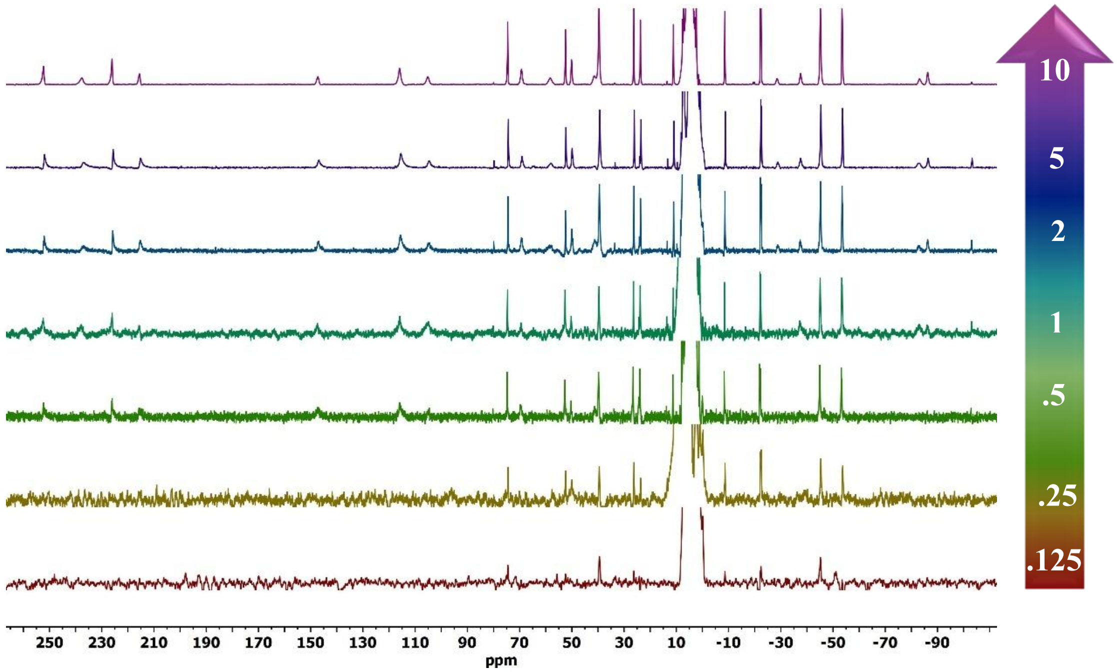

Figure S 24: ^{1}H NMR spectra to study limit of detection of $[Co(L1)]^{+}$. Conditions: 0.125 mM - 10 mM complex, pD 7.8, D_2O.

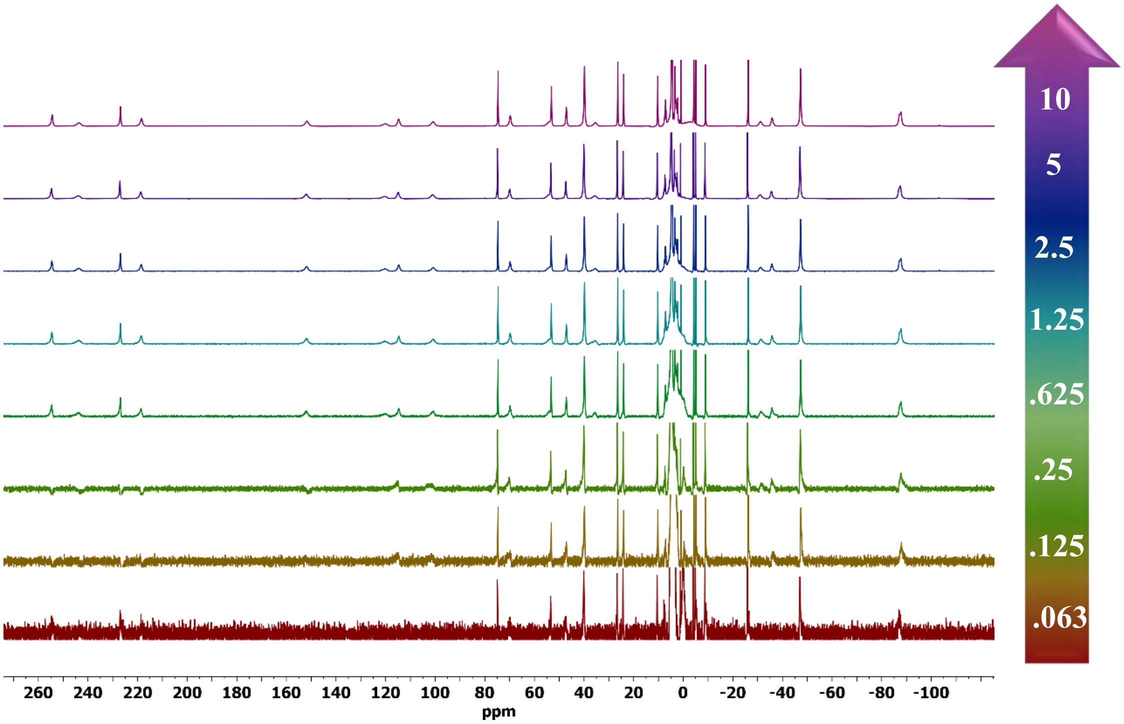

Figure S 25: ^{1}H NMR spectra to study limit of detection of $[Co(L2)]^{2+}$. Conditions: 0.063 mM - 10 mM complex, pD 7.8, D_2O.

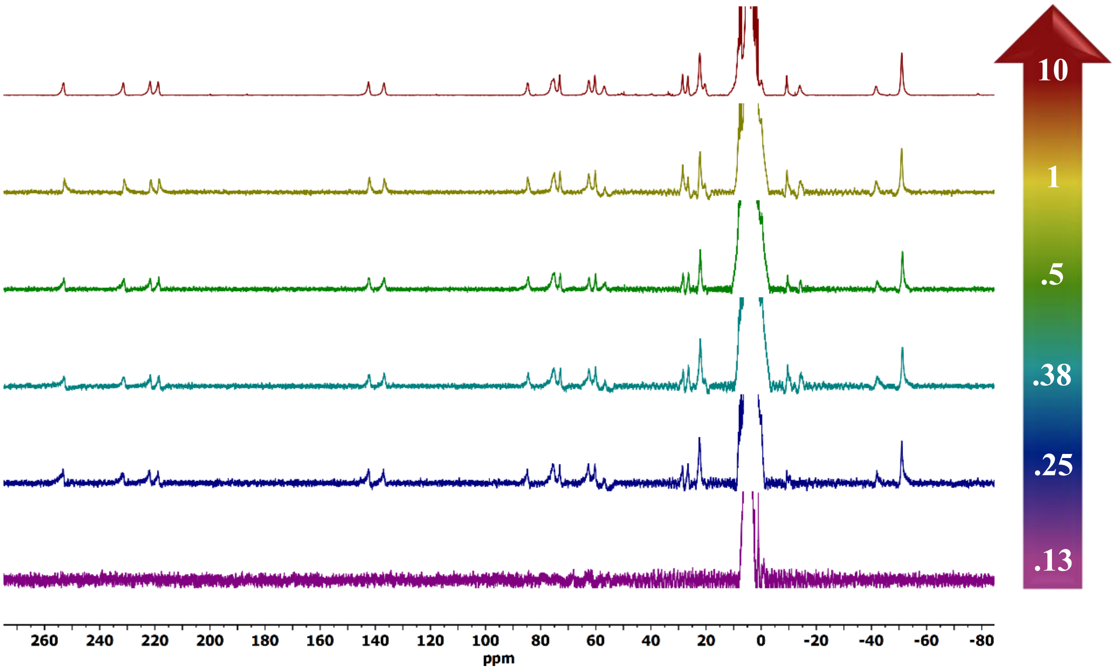

Figure S 26: ^{1}H NMR spectra to study limit of detection of $[Co(L3)]^{2+}$. Conditions: 0.13 mM - 10 mM complex, pD 7.8, D_2O.

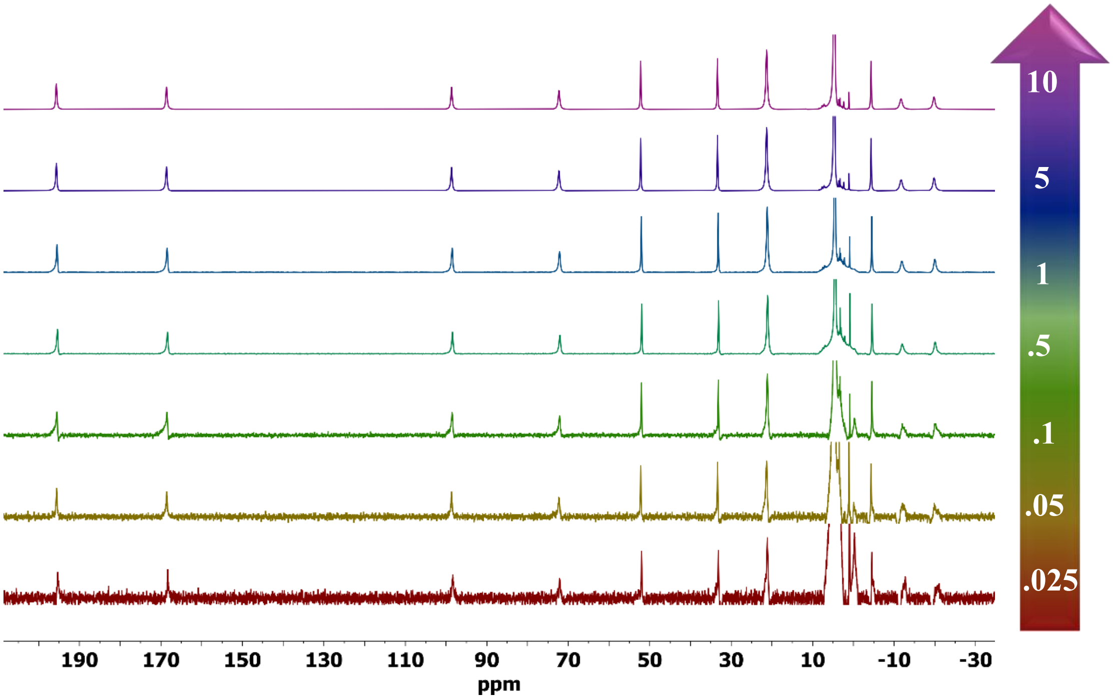

Figure S 27: ^{1}H NMR spectra to study limit of detection of $[Fe(L4)]^{2+}$. Conditions: 0.025 mM - 10 mM complex, pD 7.8, D_2O.

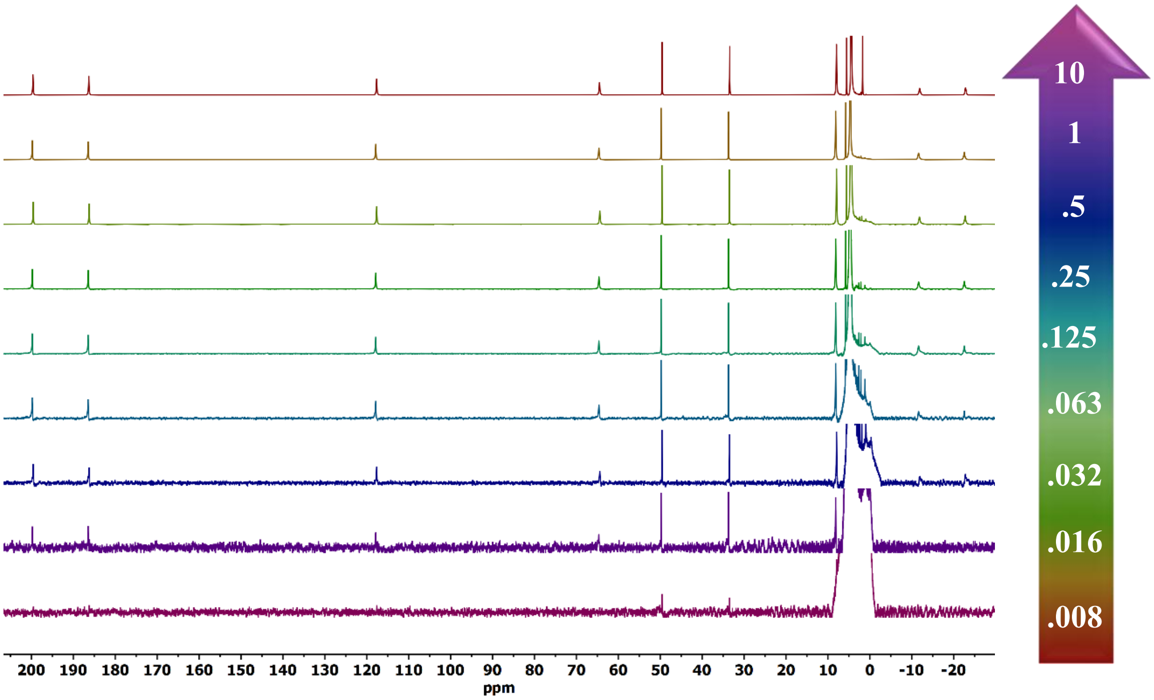

Figure S 28: ^{1}H NMR spectra to study limit of detection of $[Co(L4)]^{2+}$. Conditions: 0.008 mM - 10 mM complex, pD 7.8, D_2O.

Table S10: ^{17}O NMR data for 10 mM $[Co(L1)]^+$ solutions with and without 10 mM metabolites or fluoride.

**1/Temperature (K^{-1})**	**ln(1/T_{2r})**				
	**$[Co(L1)]^+$**	**Fluoride**	**Lactate**	**Pyruvate**	**Malate**
**0.00336**	14.2	15.2	15.2	15.3	14.9
**0.00330**	15.2	15.5	15.4	15.4	15.1
**0.00325**	15.3	15.7	15.5	15.6	15.2
**0.00319**	15.3	15.7	15.6	15.7	15.3
**0.00314**	15.2	15.8	15.5	15.7	15.3
**0.00310**	15.1	15.8	15.5	15.7	15.2
**0.00305**	14.9	15.6	15.3	15.6	15.1
**0.00300**	14.7	15.5	15.2	15.4	14.8
**0.00296**	14.6	15.3	15.0	15.2	14.7
**0.00292**	14.5	15.1	14.8	15.0	14.5
**0.00287**	14.5	15.4	14.7	15.9	14.4

Table S11: Calculated rate constant and activation parameters for water exchange in solutions containing 10 mM $[Co(L1)]^+$ with and without 10 mM metabolites or fluoride.

**Complex**	**$\Delta H^‡$ (kJ/mol)**	**$\Delta S^‡$ (J/mol K)**	**$C \times 10^9$**	**k_{ex298} (s^{-1}) $\times 10^6$**
**$[Co(L1)]^+$**	32.6	-7.28	2.67	6.13
**$[Co(L1)]^+$ & Fluoride**	40.9	20.2	4.63	4.60
**$[Co(L1)]^+$ & Lactate**	36.0	3.77	3.59	4.75
**$[Co(L1)]^+$ & Pyruvate**	38.7	12.9	4.24	4.75
**$[Co(L1)]^+$ & Malate**	35.8	0.74	2.67	3.59

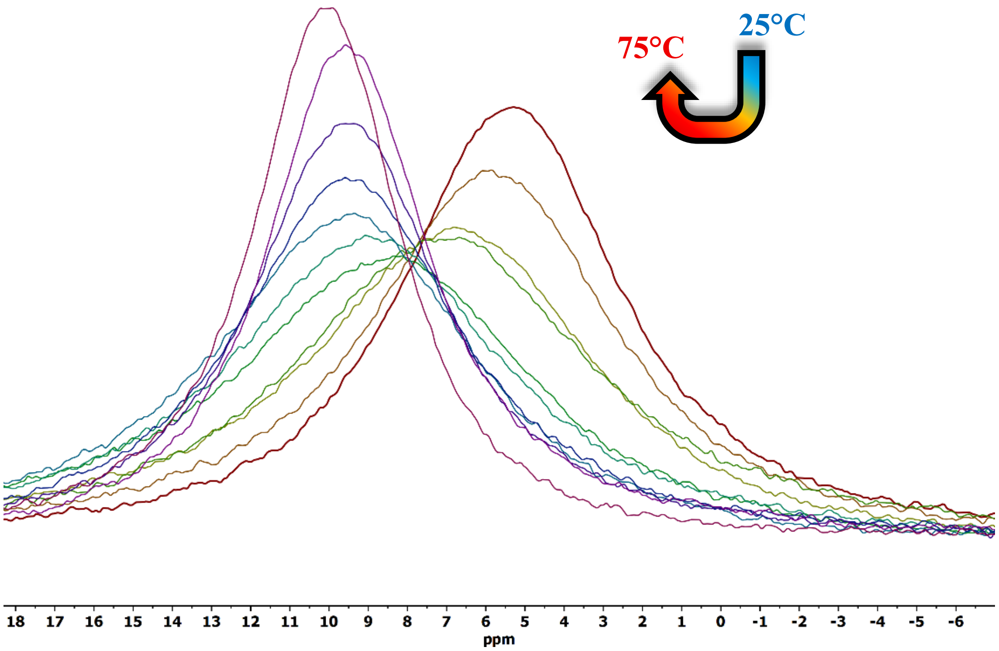

Figure S 29: Variable temperature ^{17}O NMR spectra of aqueous solutions of $[Co(L1)]^{+}$. Conditions: 10 mM complex, 0.1 M NaCl, pH 7.4, 1% (v/v) $H_2{}^{17}O$.

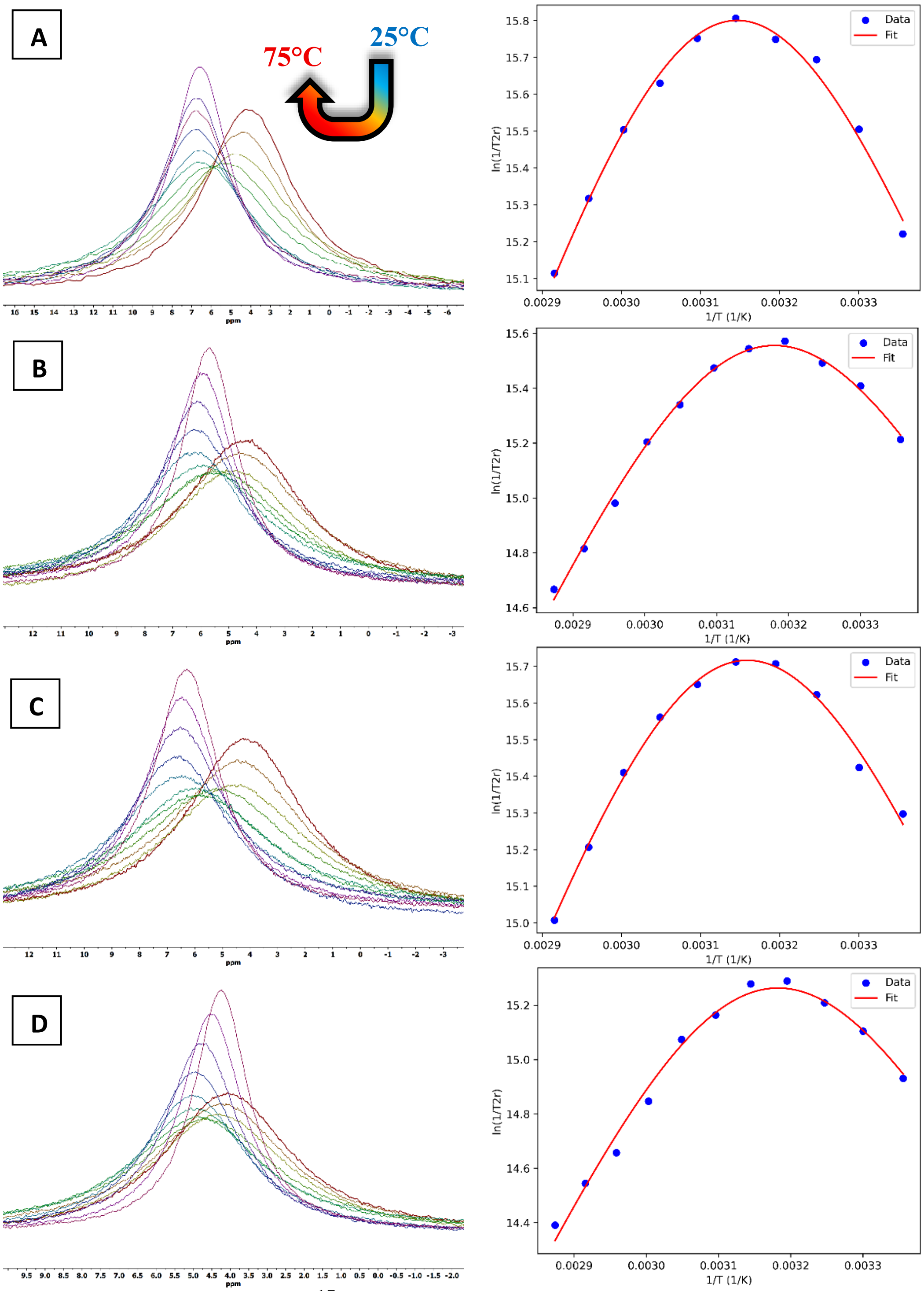

Figure S 30: Variable temperature ^{17}O NMR spectra of aqueous solutions of $[Co(L1)]^{+}$ with (A) 10 mM KF (B) 10 mM lactate (C) 10 mM pyruvate & (D) 10 mM malate on left and Swift-Connick fitting of $\ln(1/T_{2r})$ vs 1/T on right. Conditions: 10 mM complex, 0.1 M NaCl, pH 7.4, 1% (v/v) $H_2{}^{17}O$.

Table S12: ^{17}O NMR data for solutions containing 10 mM $[Co(L2)]^{2+}$.

**Temperature inverse (1/T) (K^{-1})**	**ln($1/T_{2r}$)**
**0.00336**	14.7
**0.00330**	14.9
**0.00325**	15.0
**0.00319**	15.0
**0.00314**	15.0
**0.00310**	15.0
**0.00305**	14.9
**0.00300**	14.8
**0.00296**	14.6
**0.00292**	14.6
**0.00287**	14.5

Table S 13: Calculated rate constant and activation parameters for water exchange in solutions of 10 mM $[Co(L2)]^{2+}$.

**Complex**	**$\Delta H^{\ddagger}$ (kJ/mol)**	**$\Delta S^{\ddagger}$ (J/mol K)**	**$C \times 10^{9}$**	**k_{ex298} (s^{-1}) $\times 10^{6}$**
**$[Co(L2)]^{2+}$**	27.3	-29.2	2.1	3.1

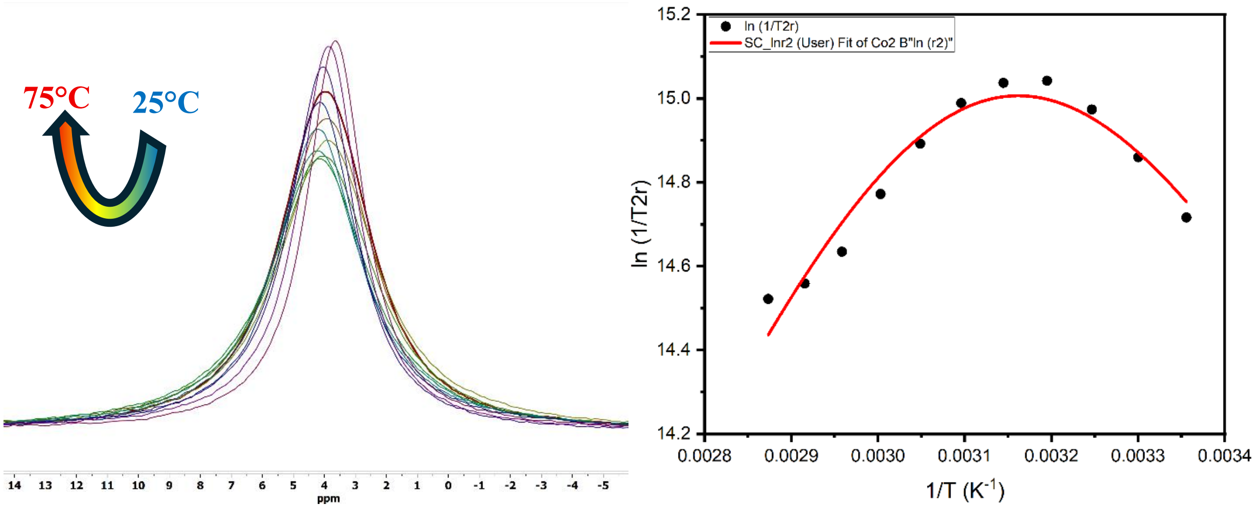

Figure S 31: Variable temperature ^{17}O NMR spectra of solutions containing $[Co(L2)]^{2+}$ on left and Swift-Connick fitting of ln($1/T_{2r}$) vs 1/T on right. Conditions: 10 mM complex, 0.1 M NaCl, pH 7.4, 1% (v/v) $H_2^{17}O$.

Table S14: ^{17}O NMR data for solutions containing 10 mM $[Co(L3)]^{2+}$.

**1/Temperature (K^{-1})**	**ln($1/T_{2r}$)**
**0.00336**	15.1
**0.00330**	15.4
**0.00325**	15.5
**0.00319**	15.5
**0.00314**	15.6
**0.00310**	15.6
**0.00305**	15.5
**0.00300**	15.4
**0.00296**	15.3
**0.00292**	15.1
**0.00287**	15.6

Table S15: Calculated rate constant and activation parameters for water exchange in solutions containing 10 mM $[Co(L3)]^{2+}$.

**Complex**	**$\Delta H^{\ddagger}$ (kJ/mol)**	**$\Delta S^{\ddagger}$ (J/mol K)**	**$C \times 10^9$**	**k_{ex298} (s^{-1}) $\times 10^6$**
**$[Co(L3)]^{2+}$**	32	-9.7	3.79	5.94

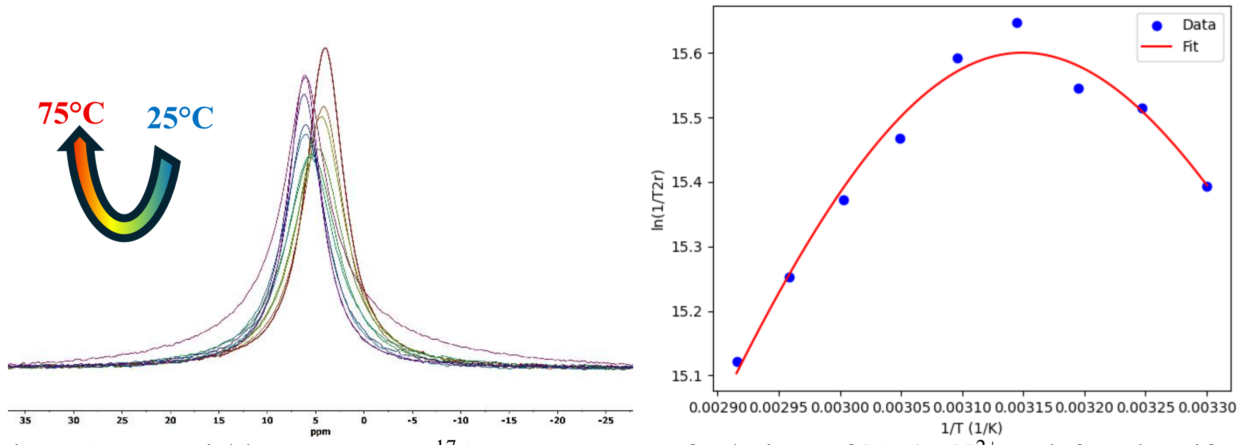

Figure S 32: Variable temperature ^{17}O NMR spectra of solutions of $[Co(L3)]^{2+}$ on left and Swift-Connick fitting of ln($1/T_{2r}$) vs 1/T on right. Conditions: 10 mM complex, 0.1 M NaCl, pH 7.4, 1% (v/v) $H_2{}^{17}O$.

Table S16: ^{17}O NMR data for solutions of 10 mM $[Fe(L1)]^+$ with and without 10 mM metabolite anions.

**1/Temperature (K^{-1})**	**ln(1/T_{2r})**			
	**$[Fe(L1)]^+$**	**Lactate**	**Pyruvate**	**100 mM Pyruvate**
**0.00336**	15.2	13.4	12.4	12.5
**0.00330**	15.2	13.5	12.5	12.7
**0.00325**	15.2	13.4	12.5	12.7
**0.00319**	15.1	13.5	12.7	12.8
**0.00314**	14.9	13.5	12.6	12.8
**0.00310**	14.7	13.3	12.5	12.8
**0.00305**	14.5	13.3	12.3	12.7
**0.00300**	14.4	13.3	12.4	12.7
**0.00296**	14.2	13.3	12.3	12.6
**0.00292**	14.1	13.3	12.6	12.6
**0.00287**	13.9	13.2	12.8	12.7

Table S17: Calculated rate constant and activation parameters for water exchange in solutions of 10 mM $[Fe(L1)]^+$.

**Complex**	**$\Delta H^‡$ (kJ/mol)**	**$\Delta S^‡$ (J/mol K)**	**C × 10^9**	**k_{ex298} (s^{-1}) × 10^6**
$[Fe(L1)]^+$	28.7	-16.7	2.48	7.83

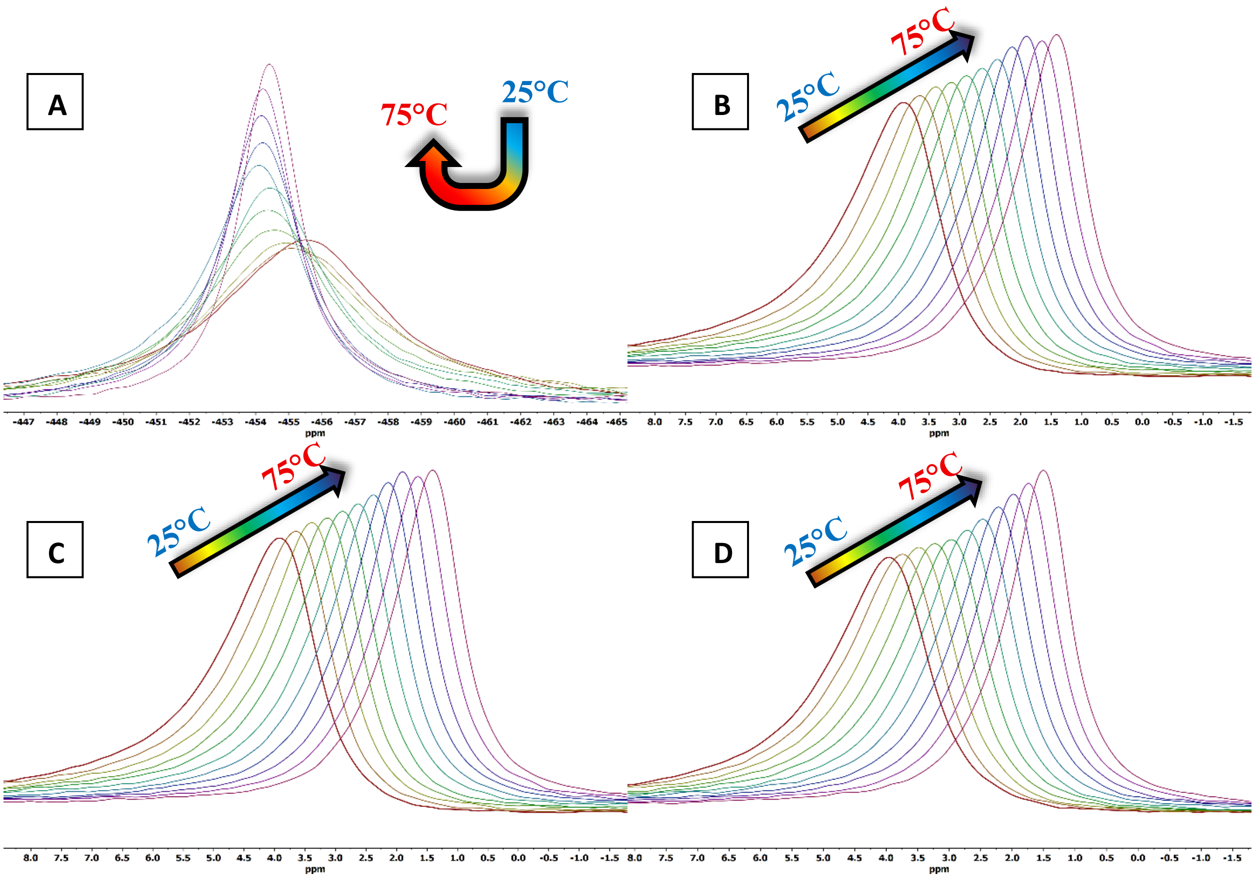

Figure S 33: Variable temperature ^{17}O NMR spectra of solutions containing: (A) $[Fe(L1)]^+$, with (B) 10 mM lactate (C) 10 mM pyruvate & (D) 100 mM pyruvate. Conditions: 10 mM complex, 0.1 M NaCl, pH 4 (A), pH 7.4 (B, C, D), 1% (v/v) $H_2{}^{17}O$.

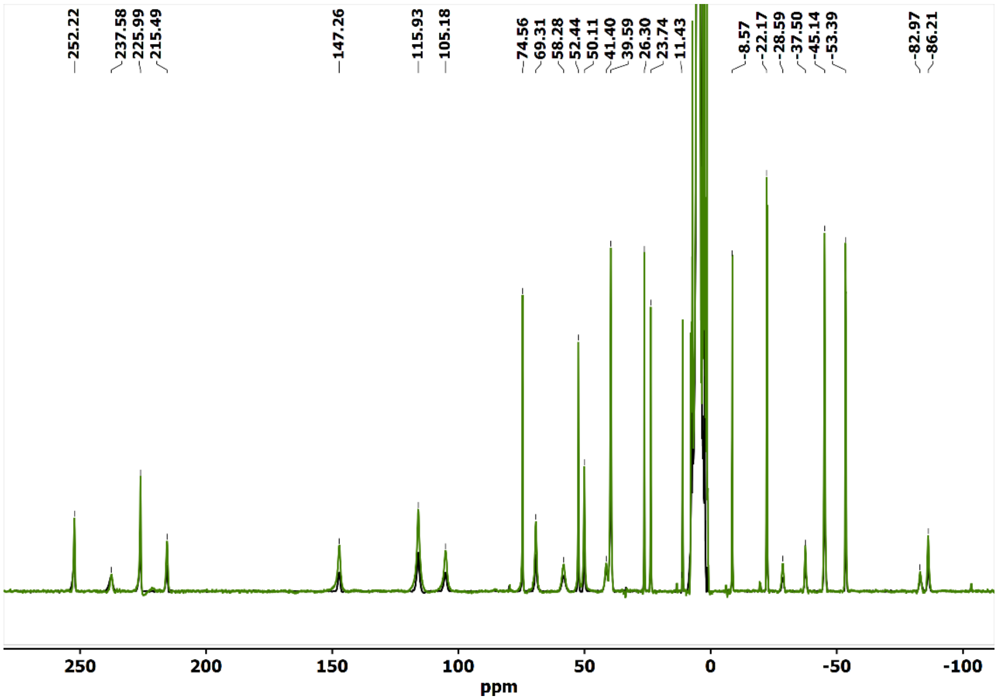

Figure S 34: ^{1}H NMR spectra of $[Co(L1)]^+$ (black) upon addition of 10 mM lactate (green). Conditions: 10 mM complex, pD 7.8, D_2O.

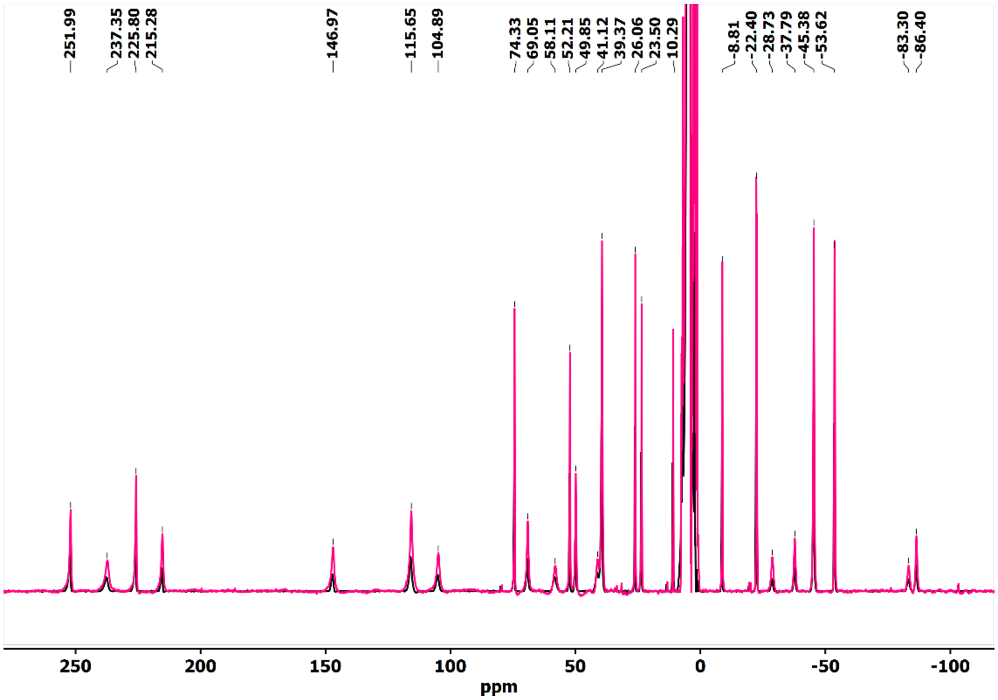

Figure S 35: ^{1}H NMR spectra of $[Co(L1)]^+$ (black) upon addition of 10 mM pyruvate (pink). Conditions: 10 mM complex, pD 7.8, D_2O.

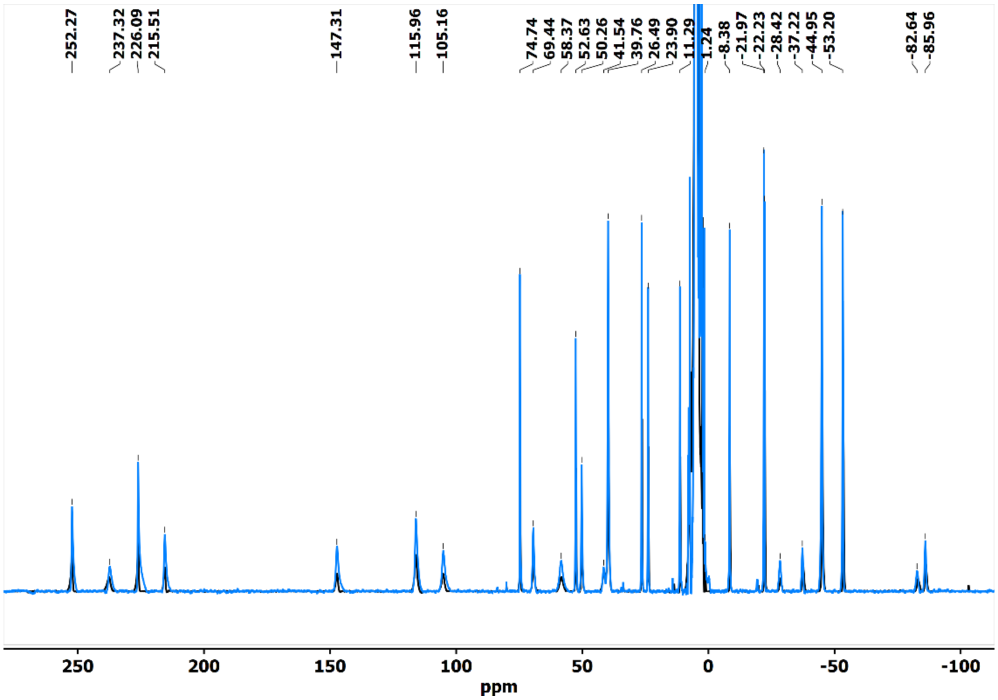

Figure S 36: ^{1}H NMR spectra of $[Co(L1)]^+$ (black) upon addition of 10 mM malate (blue). Conditions: 10 mM complex, pD 7.8, D_2O.

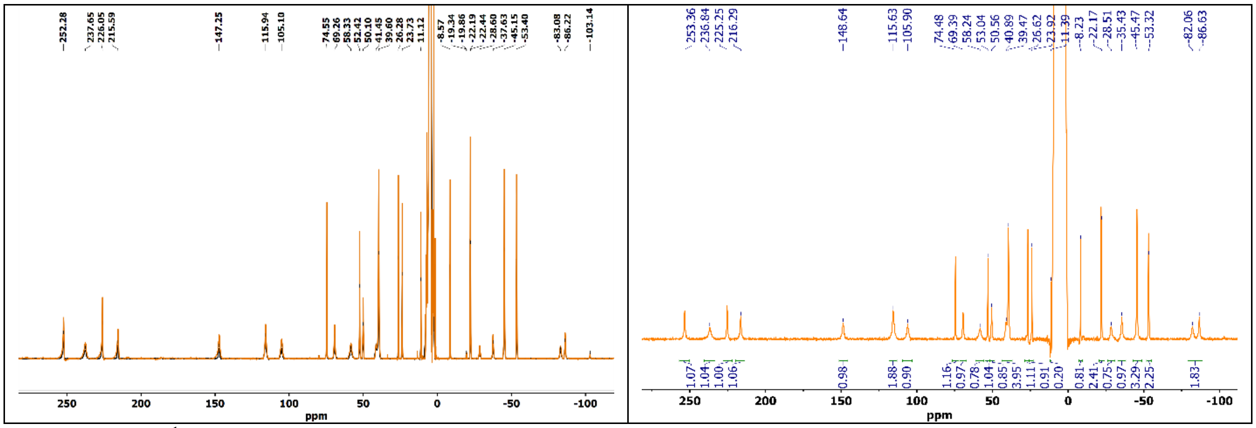

Figure S 37: ^{1}H NMR spectra of $[Co(L1)]^+$ (black) upon addition of 10 mM trifluorolactate (orange). Conditions: 10 mM complex, pD 7.8 D_2O (left), pH 7.4 H_2O (right).

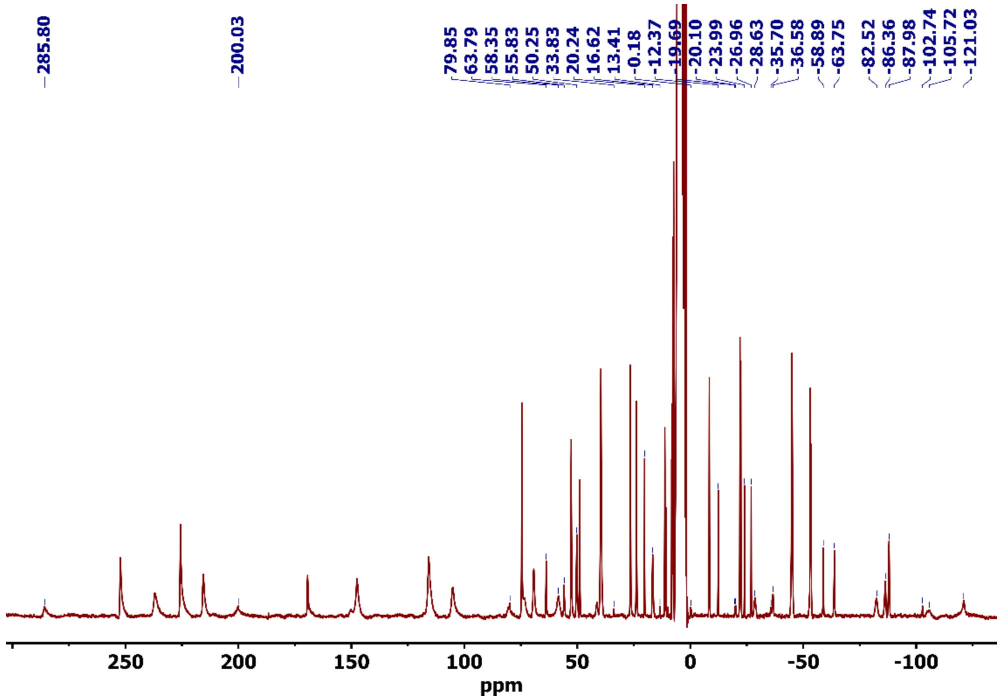

Figure S 38: ^{1}H NMR spectra of $[Co(L1)]^{+}$ upon addition of 10 eq. KF. Conditions: 10 mM complex, 100 mM KF, pD 7.8 D_2O. New proton resonances are labeled.

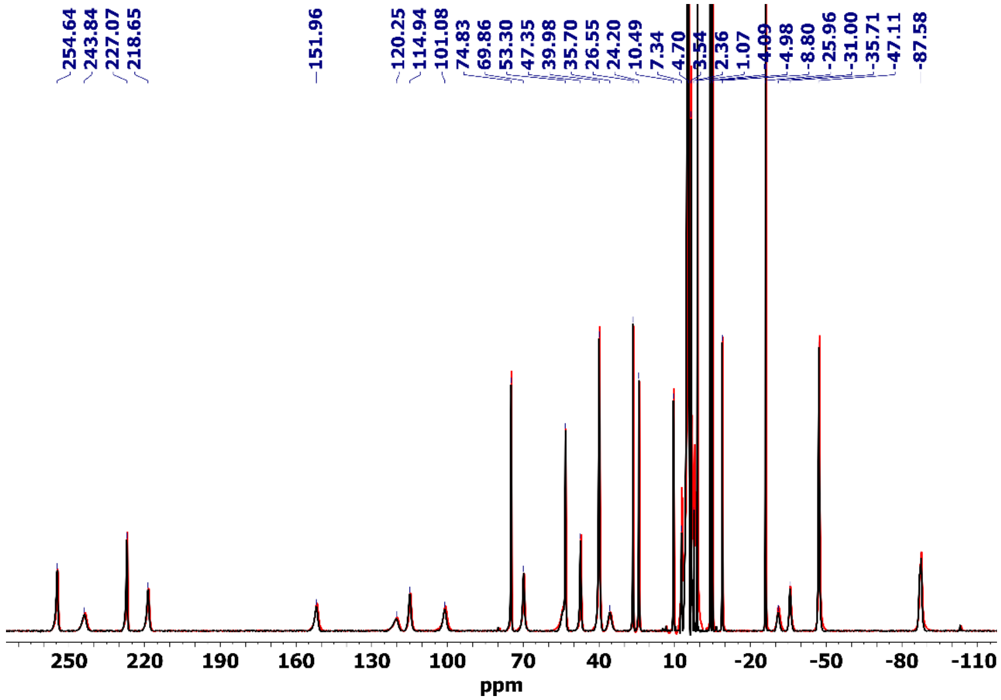

Figure S 39: ^{1}H NMR spectra of $[Co(L2)]^{2+}$ (black) upon addition of 10 mM lactate (red). Conditions: 10 mM complex, pD 7.8, D_2O.

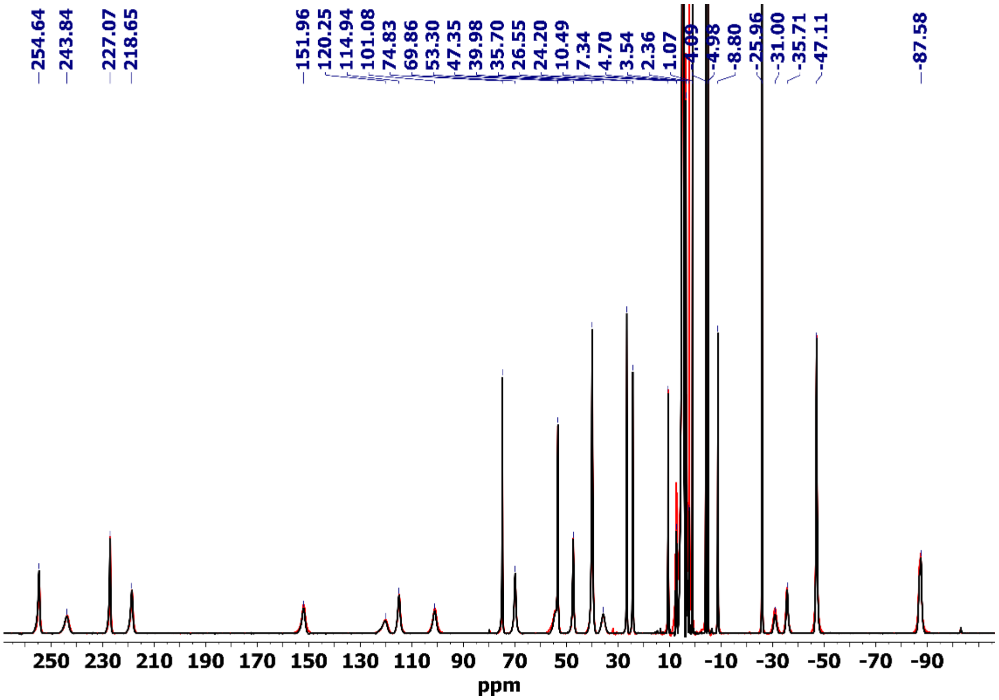

Figure S 40: ^{1}H NMR spectra of $[Co(L2)]^{2+}$ (black) upon addition of 10 mM pyruvate (red). Conditions: 10 mM complex, pD 7.8, D_2O.

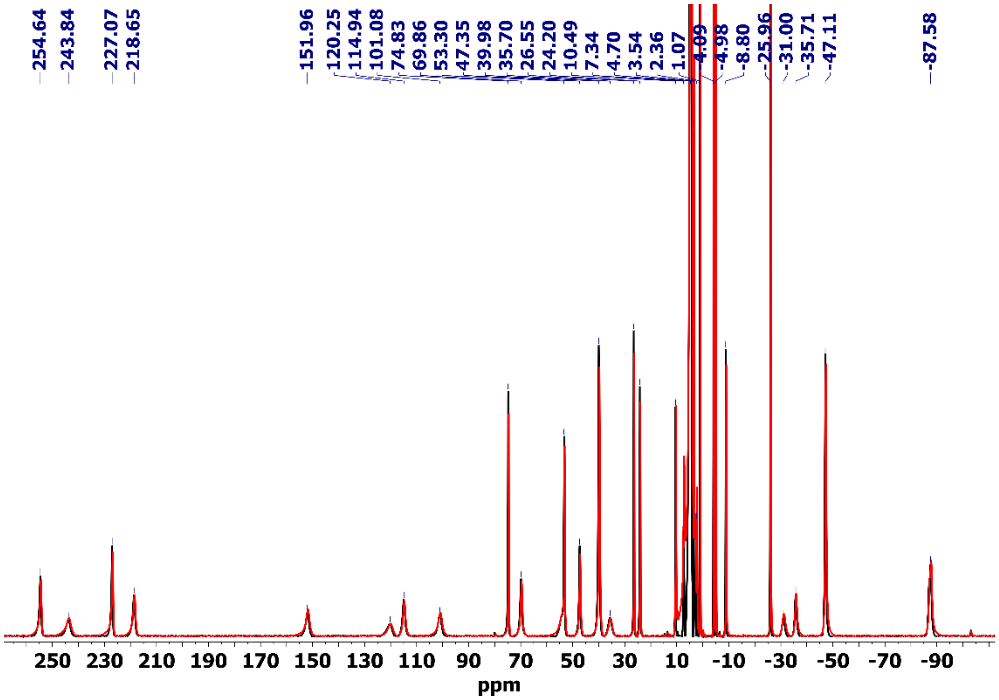

Figure S 41: ^{1}H NMR spectra of $[Co(L2)]^{2+}$ (black) upon addition of 10 mM trifluorolactate (red). Conditions: 10 mM complex, pD 7.8, D_2O.

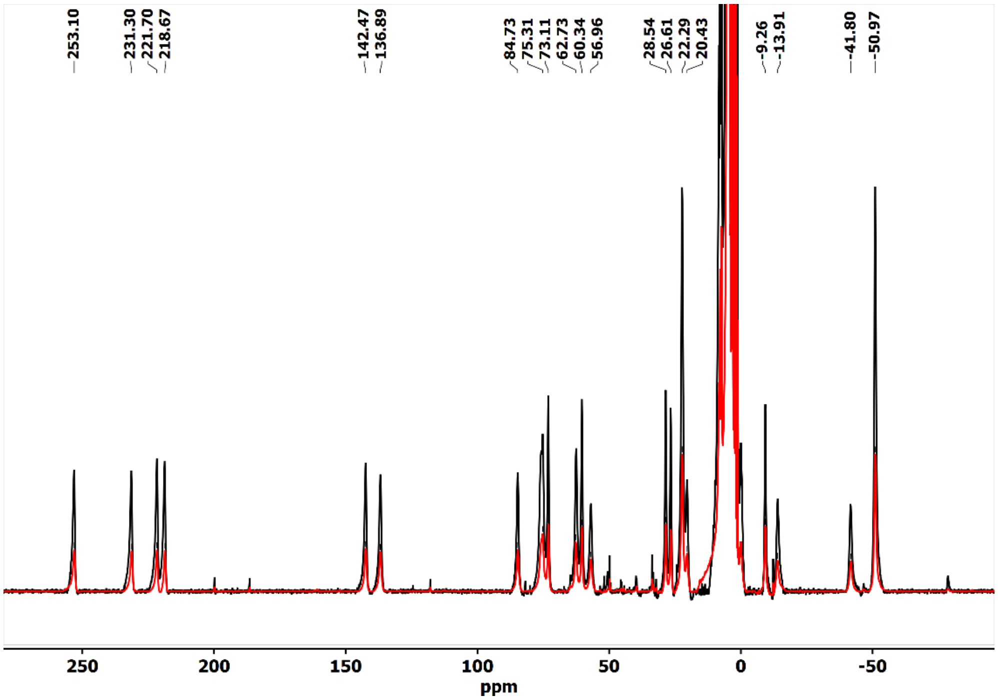

Figure S 42: ^{1}H NMR spectra of $[Co(L3)]^{2+}$ (black) upon addition of 10 mM KF (red). Conditions: 10 mM complex, pD 7.8, D_2O.

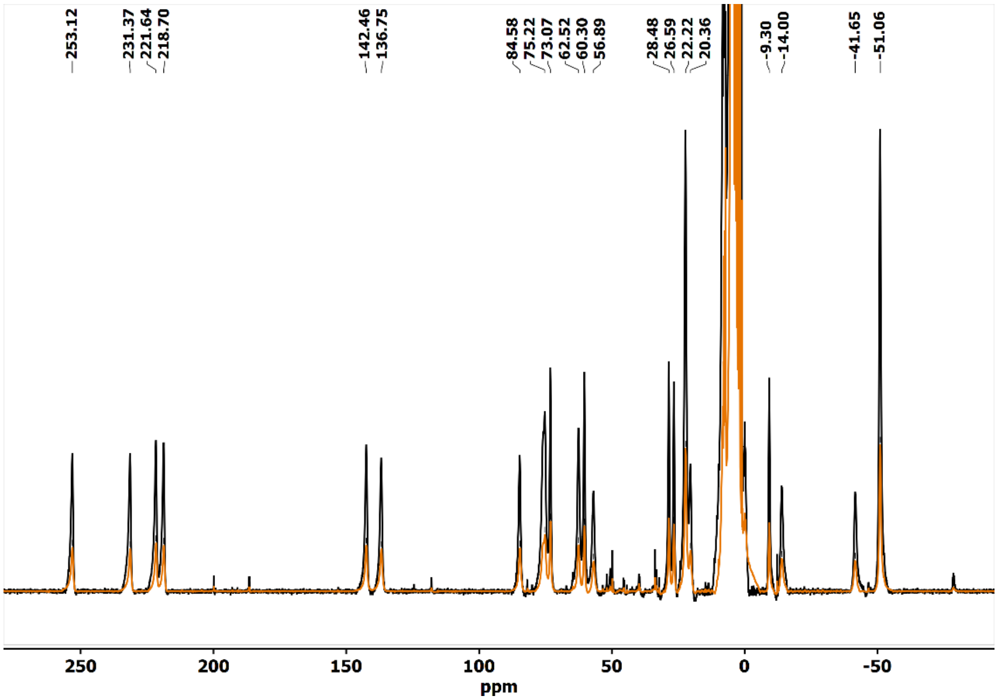

Figure S 43: ^{1}H NMR spectra of $[Co(L3)]^{2+}$ (black) upon addition of 10 mM trifluorolactate (orange). Conditions: 10 mM complex, pD 7.8, D_2O.

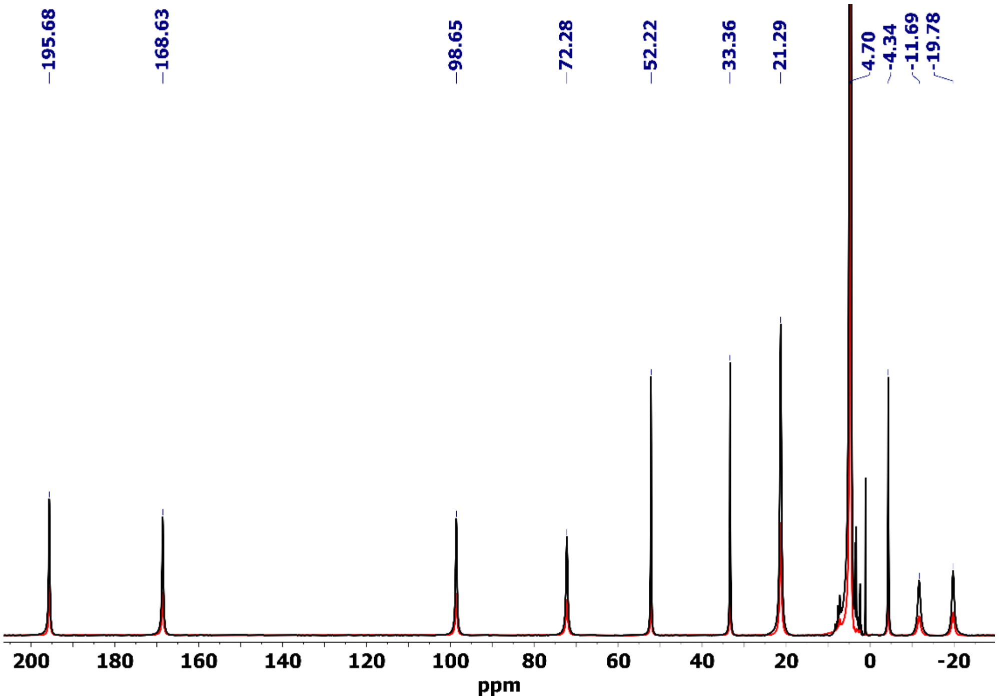

Figure S 44: ^{1}H NMR spectra of $[Fe(L4)]^{2+}$ (black) upon addition of 10 mM KF (red). Conditions: 10 mM complex, pD 7.8, D_2O.

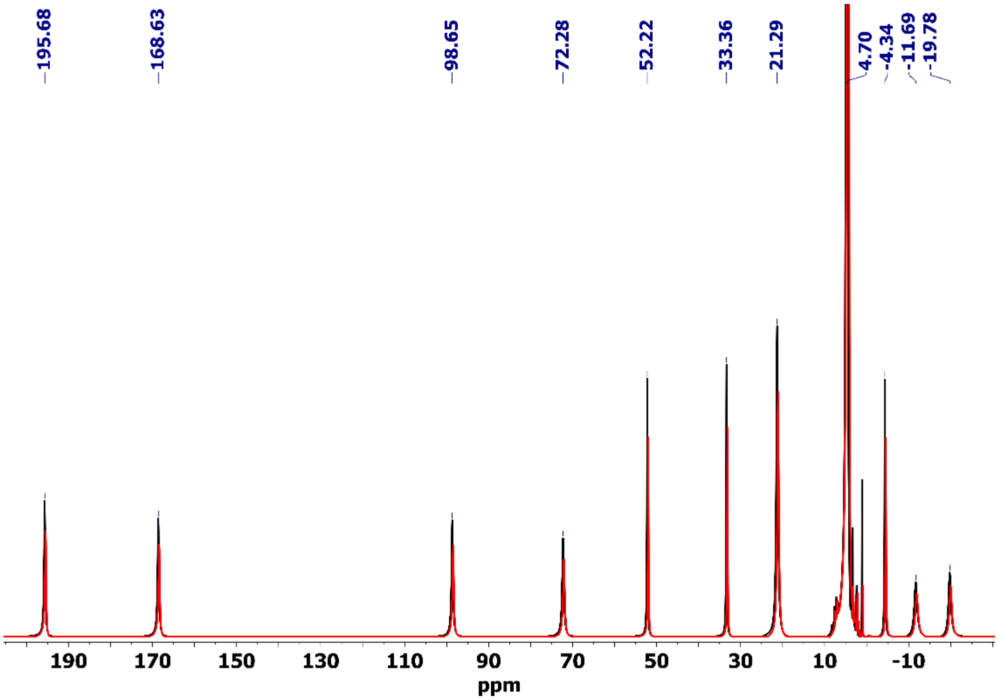

Figure S 45: ^{1}H NMR spectra of $[Fe(L4)]^{2+}$ (black) upon addition of 10 mM trifluorolactate (orange). Conditions: 10 mM complex, pD 7.8, D_2O.

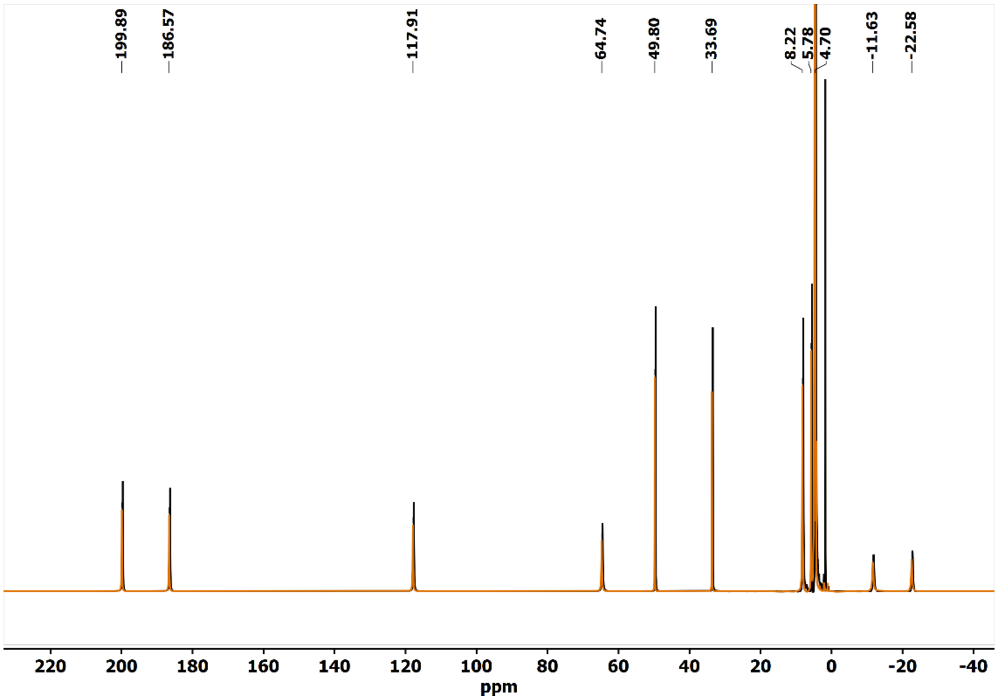

Figure S 46: ^{1}H NMR spectra of $[Co(L4)]^{2+}$ (black) upon addition of 10 mM trifluorolactate (orange). Conditions: 10 mM complex, pD 7.8, D_2O.

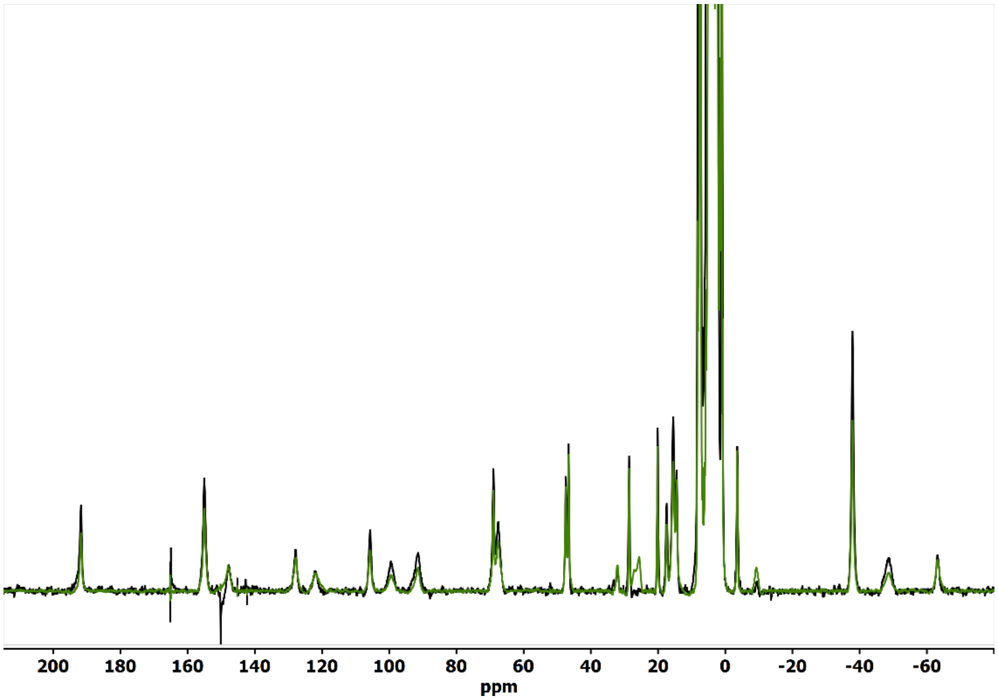

Figure S 47: ^{1}H NMR spectra of $[Fe(L1)]^{+}$ (10 mM, black pD 4.4) upon addition of 10 mM lactate (green pD 7.8). Conditions: D_2O.

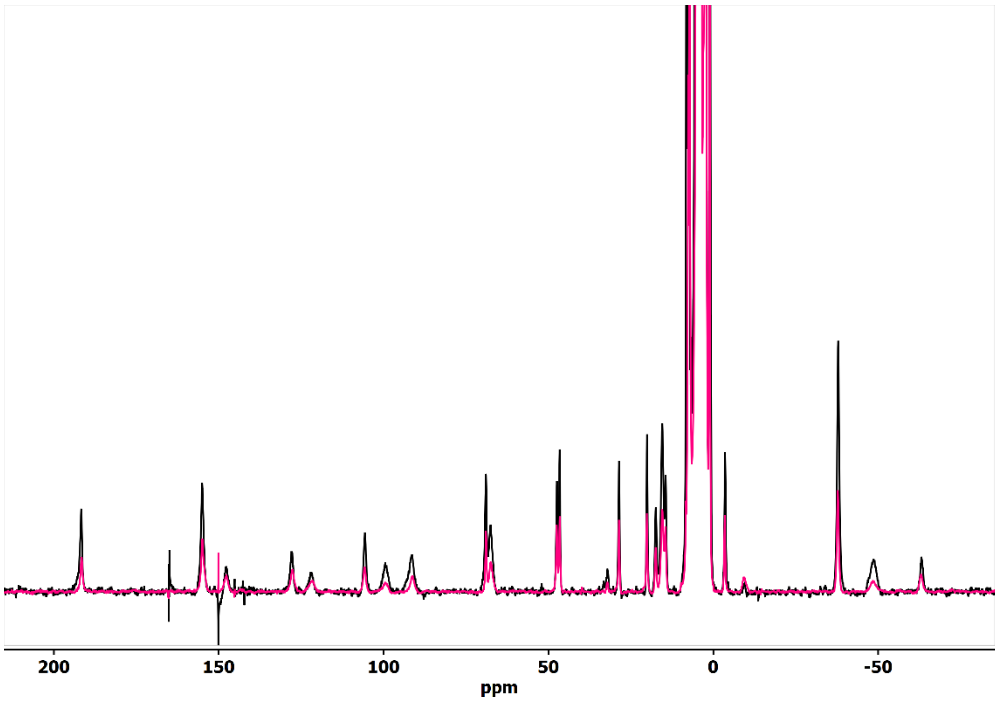

Figure S 48: ^{1}H NMR spectra of $[Fe(L1)]^{+}$ (10 mM, black pD 4.4) upon addition of 10 mM pyruvate (pink pD 7.8). Conditions: D_2O.

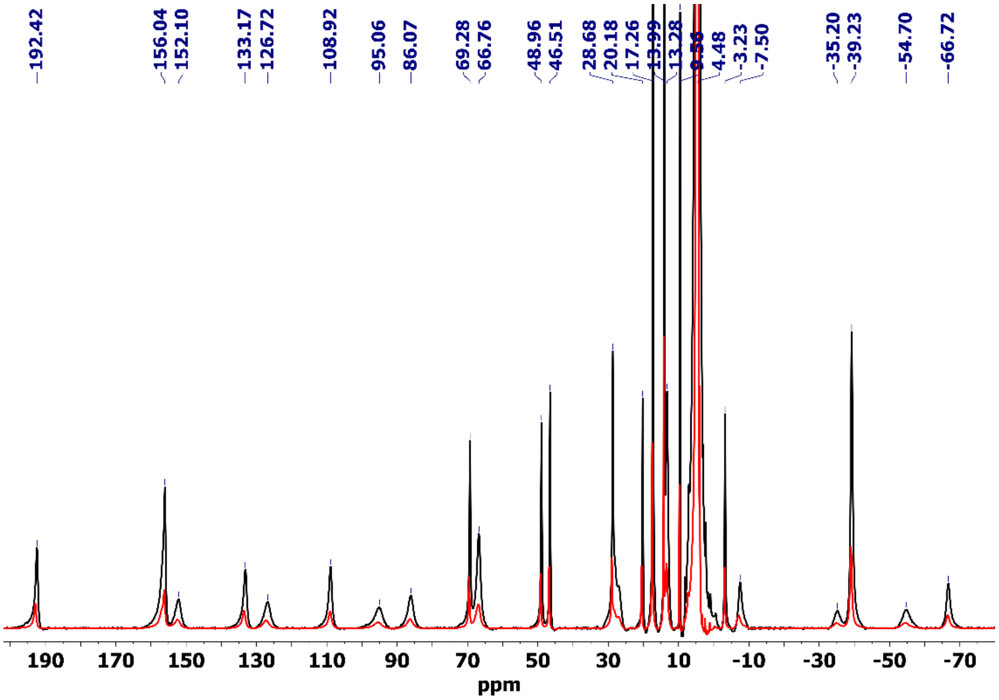

Figure S 49: ^{1}H NMR spectra of $[Fe(L2)]^{2+}$ (black) upon addition of 10 mM trifluorolactate (red). Conditions: 10 mM complex, pD 7.8, D_2O.

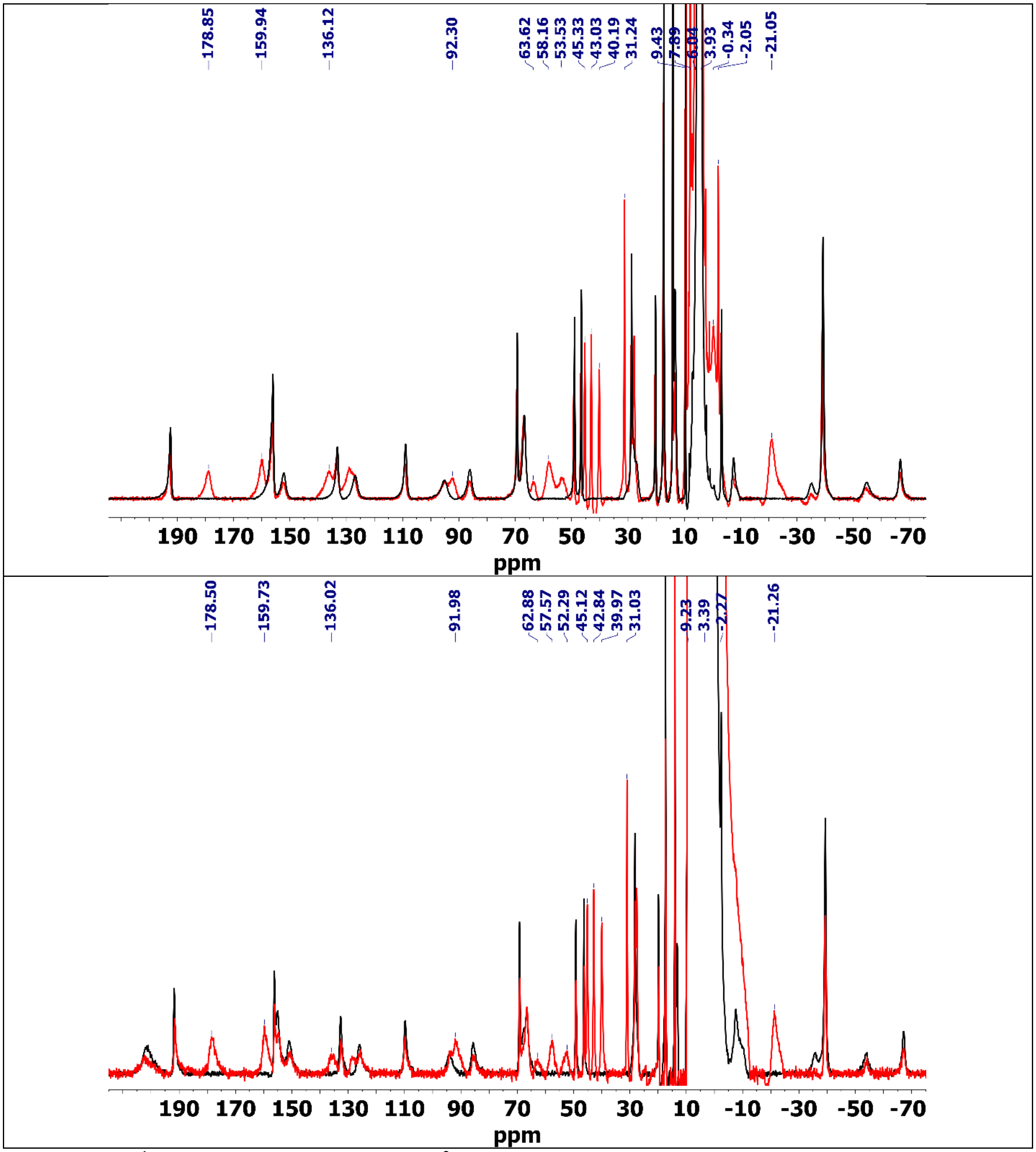

Figure S 50: ^{1}H NMR spectra of $[Fe(L2)]^{2+}$ (black) upon addition of 25 mM KF (red). Conditions: 5 mM complex, pD 7.8 D_2O (top), pH 7.4 H_2O (bottom). New proton resonances are labeled. In H_2O, there is a new resonance at about 200 ppm that does not appear in D2O which is assigned as a protonated group.

Table S 18: Binding constants from the fluoride NMR titrations at 25 °C, 0.1 M NaCl, pD 7.8.

**Complex**	**Binding constant K_d (mM)**	
	**^{1}H NMR**	**^{19}F NMR**
**$[CoL1]^{+}$**	2.5×10^{2}	-
**$[FeL1]^{+}$**	13	-
**$[CoL2]^{2+}$**	1.8×10^{2}	-
**$[FeL2]^{2+}$**	17 (16 in H_2O)	2.6 (3.1 in H_2O)

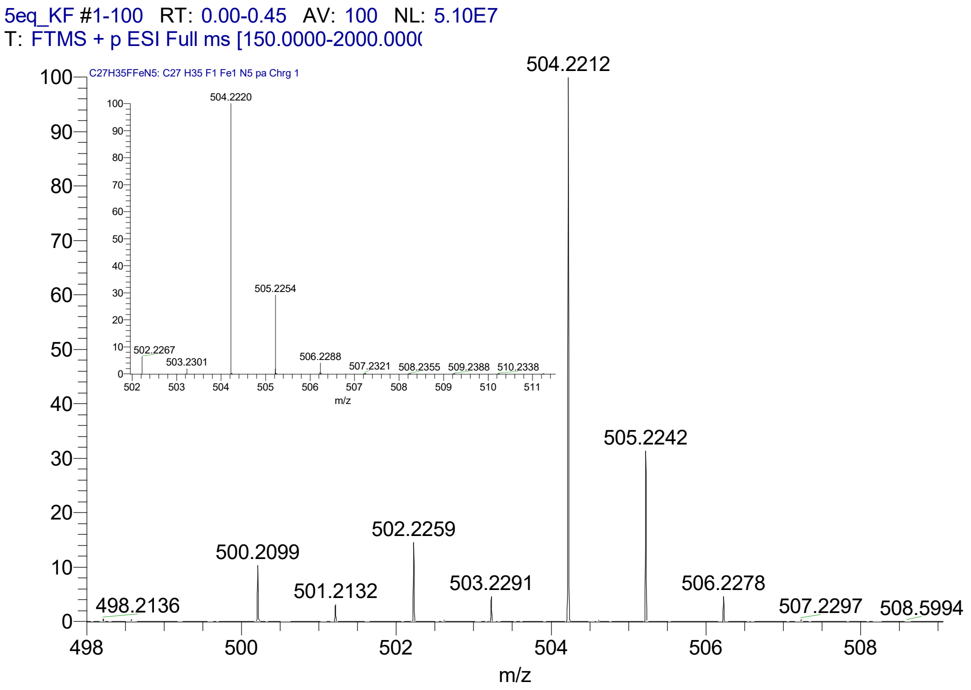

Figure S 51: High resolution mass spectrometry of $[Fe(L2)]^{2+}$ upon addition of 5 eq. KF Conditions: 100 µM complex, 500 µM KF, pH 7.4 H_2O. Insert shows the simulated spectra of $[Fe(L2)(F)]^{+}$ species.

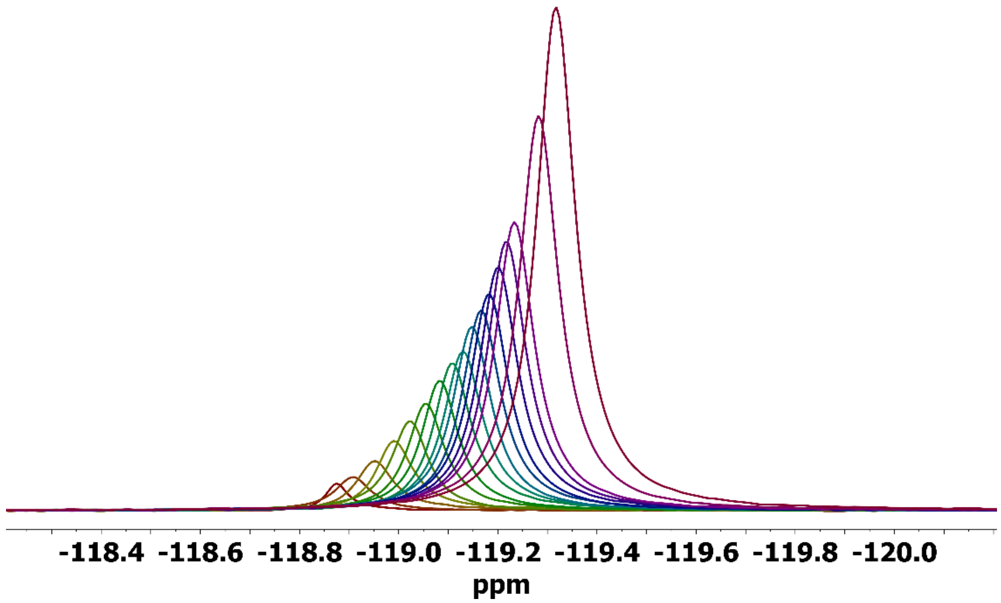

Figure S 52: ^{19}F NMR spectra for titration of $[Fe(L2)]^{2+}$ with fluoride. Conditions: 5 mM complex, 1 - 25 mM KF, pH 7.4, H_2O, 0.1 M NaCl.

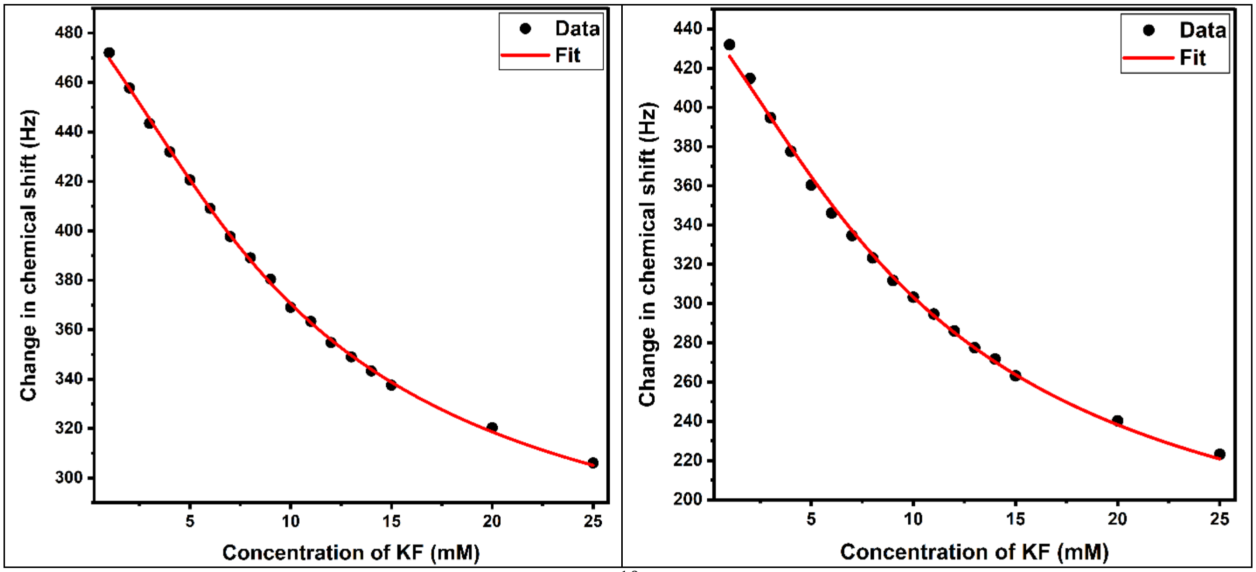

Figure S 53: Plot of change in chemical shift of ^{19}F NMR vs concentration of KF with fitting curve to determine binding constant for $[Fe(L2)]^{2+}$ with fluoride. pD 7.8 D_2O (left), pH 7.4 H_2O (right).

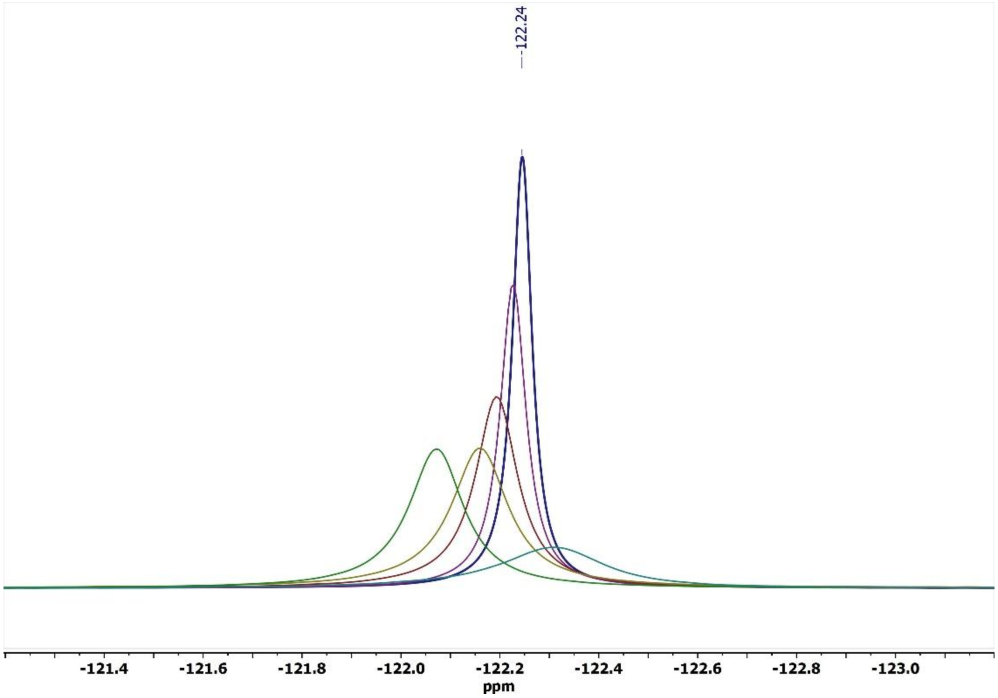

Figure S 54: ^{19}F NMR spectra for titration of fluoride with $[Fe(L1)]^{+}$. Conditions: 0 – 10 mM complex, 10 mM KF, pD 7.8, D_2O, 0.1 M NaCl.

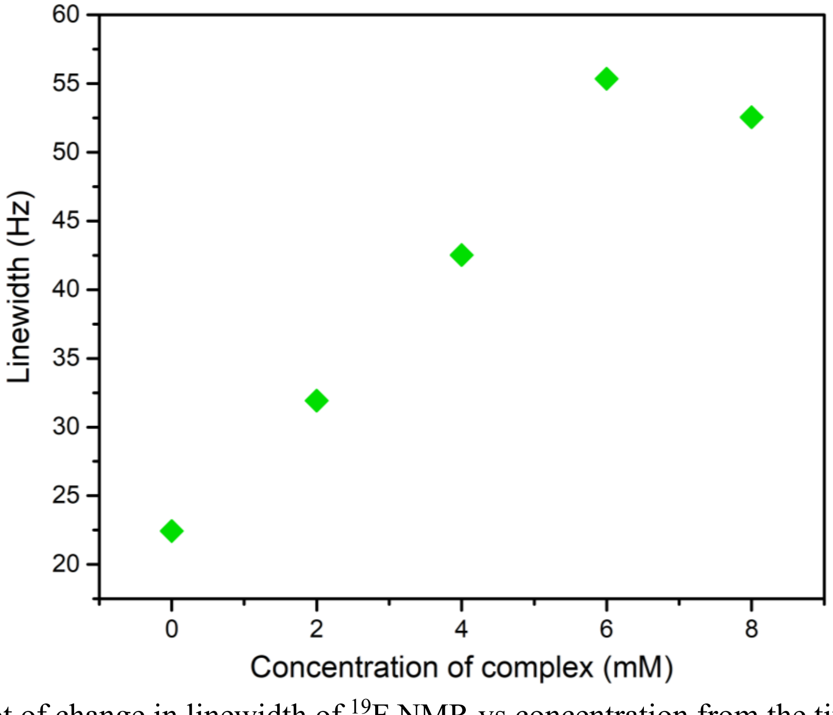

Figure S 55: Plot of change in linewidth of ^{19}F NMR vs concentration from the titration of fluoride with $[Fe(L1)]^{+}$.

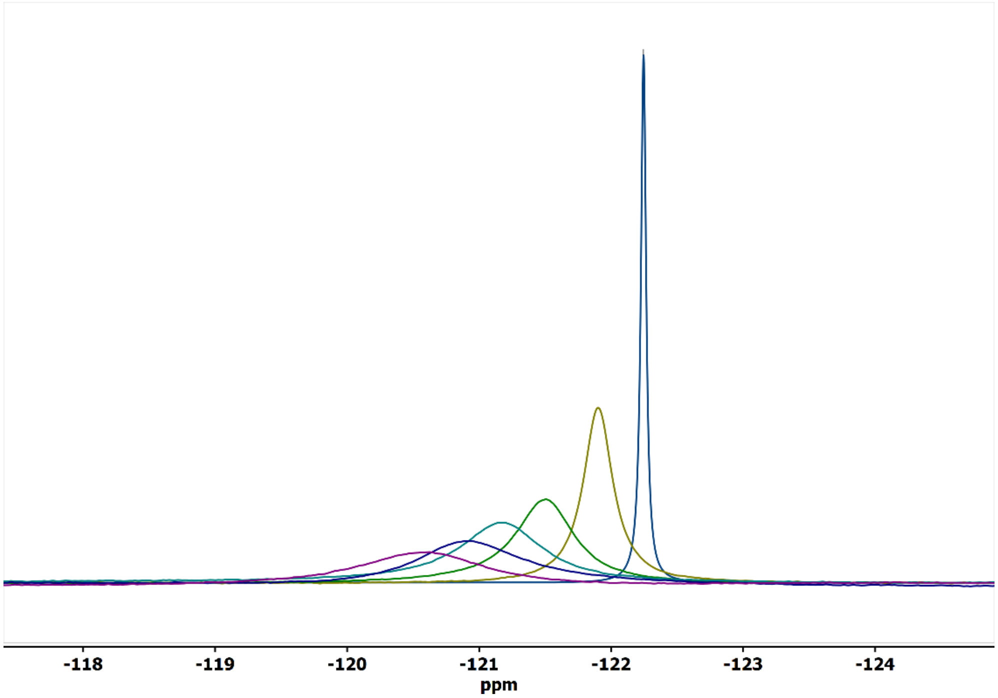

Figure S 56: ^{19}F NMR spectra for titration of fluoride with $[Co(L1)]^+$. Conditions: 0 – 10 mM complex, 10 mM KF, pD 7.8, D_2O, 0.1 M NaCl.

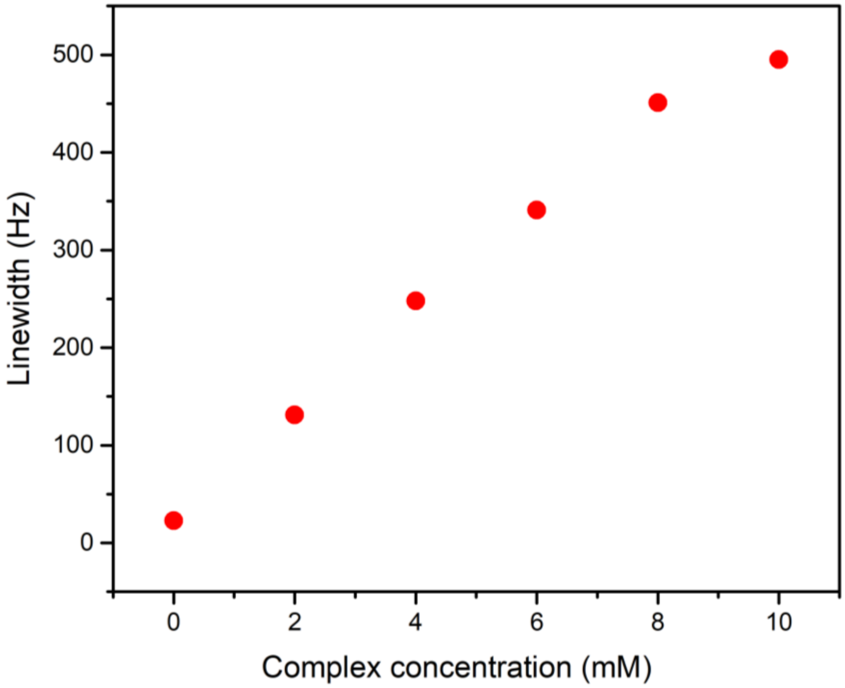

Figure S 57: Plot of change in linewidth of ^{19}F NMR vs concentration from the titration of fluoride with $[Co(L1)]^+$.

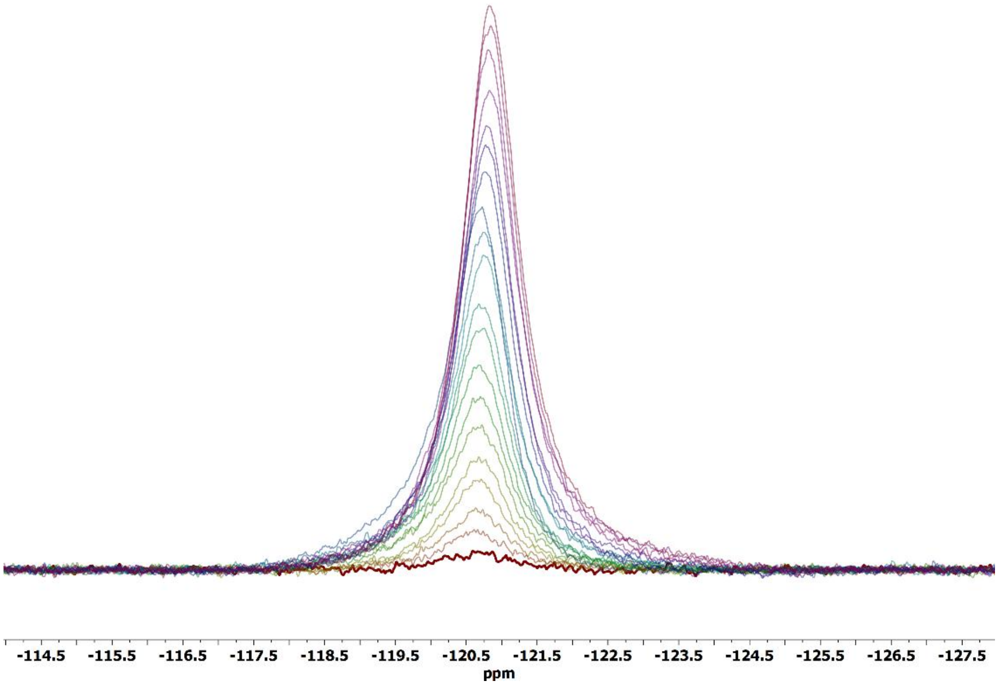

Figure S 58: ^{19}F NMR titration of fluoride with $[Co(L1)]^{+}$. Conditions: 10 mM complex, 1 - 20 mM KF, 3 mM trifluoroethanol, pD 7.8, D_2O, 0.1 M NaCl.

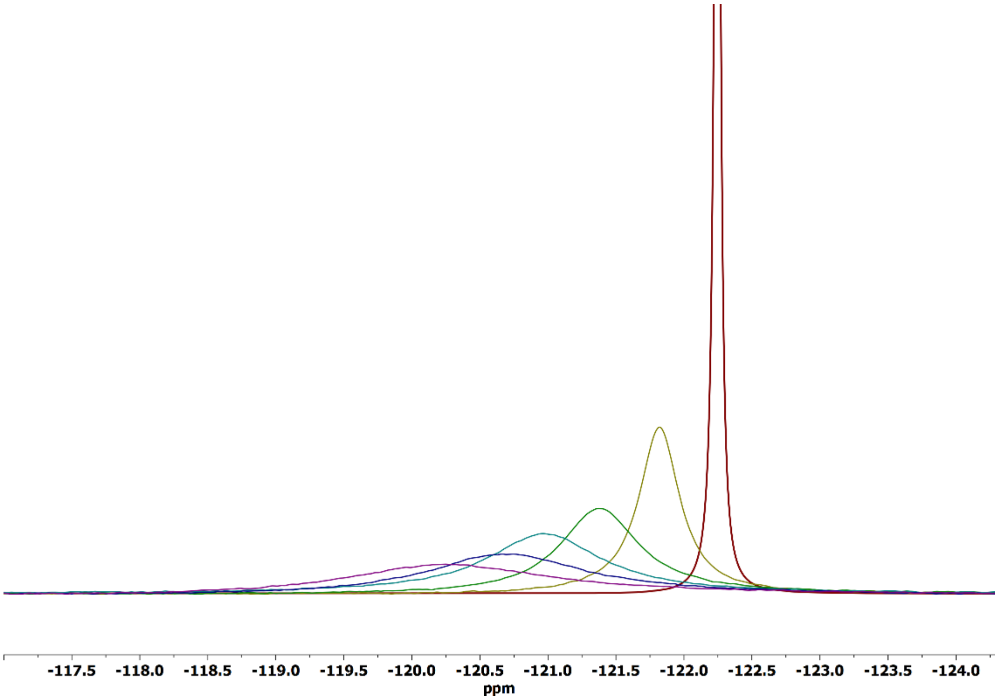

Figure S 59: ^{19}F NMR titration of fluoride with $[Co(L3)]^{2+}$. Conditions: 0 – 10 mM complex, 10 mM KF, pD 7.8, D_2O, 0.1 M NaCl.

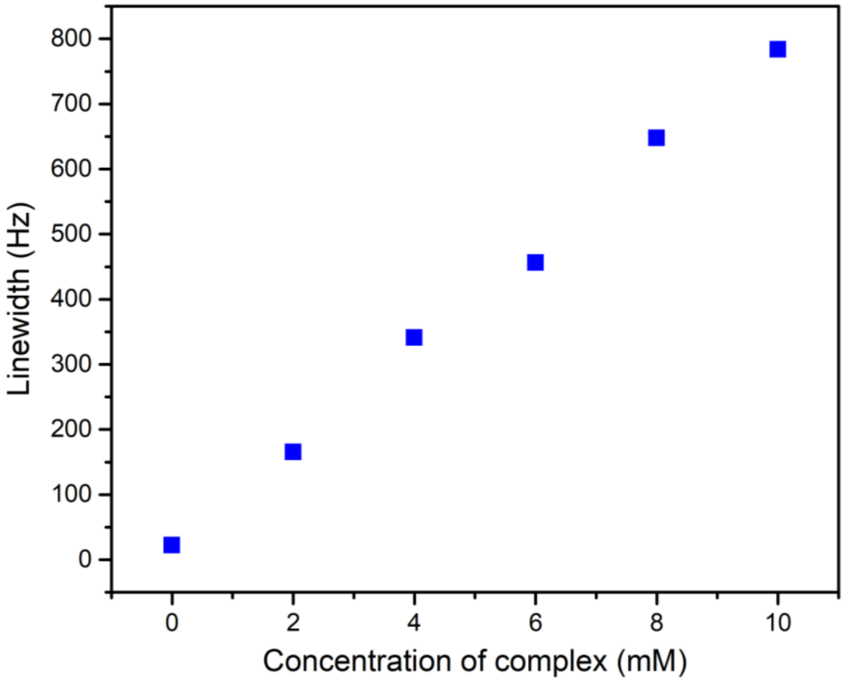

Figure S 60: Plot of change in linewidth of ^{19}F NMR vs concentration from the titration of fluoride with $[Co(L3)]^{2+}$.

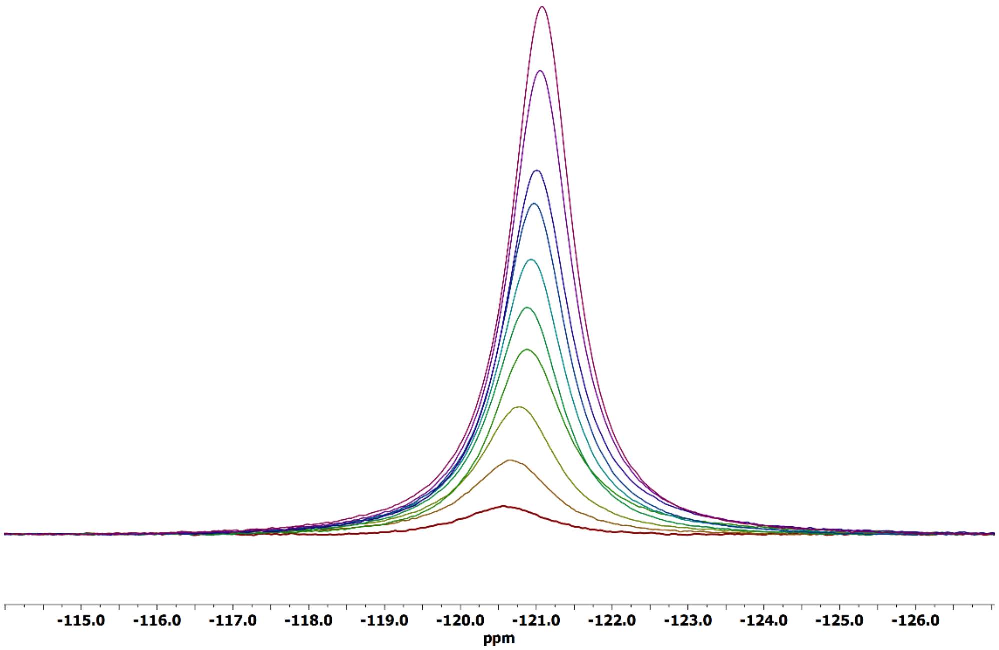

Figure S 61: ^{19}F NMR titration of $[Co(L3)]^{2+}$ with fluoride. Conditions: 10 mM complex, 10 - 100 mM KF, pD 7.8, D_2O, 0.1 M NaCl.

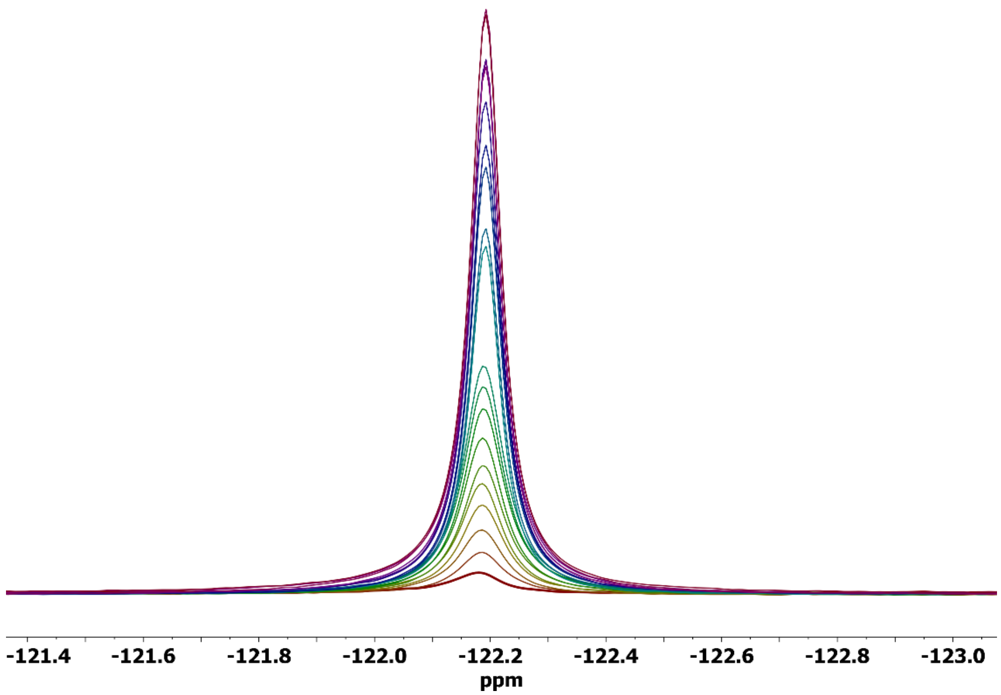

Figure S 62: ^{19}F NMR titration of $[Fe(L4)]^{2+}$ with fluoride. Conditions: 10 mM complex, 1 - 20 mM KF, 3 mM trifluoroethanol, pD 7.8, D_2O, 0.1 M NaCl.

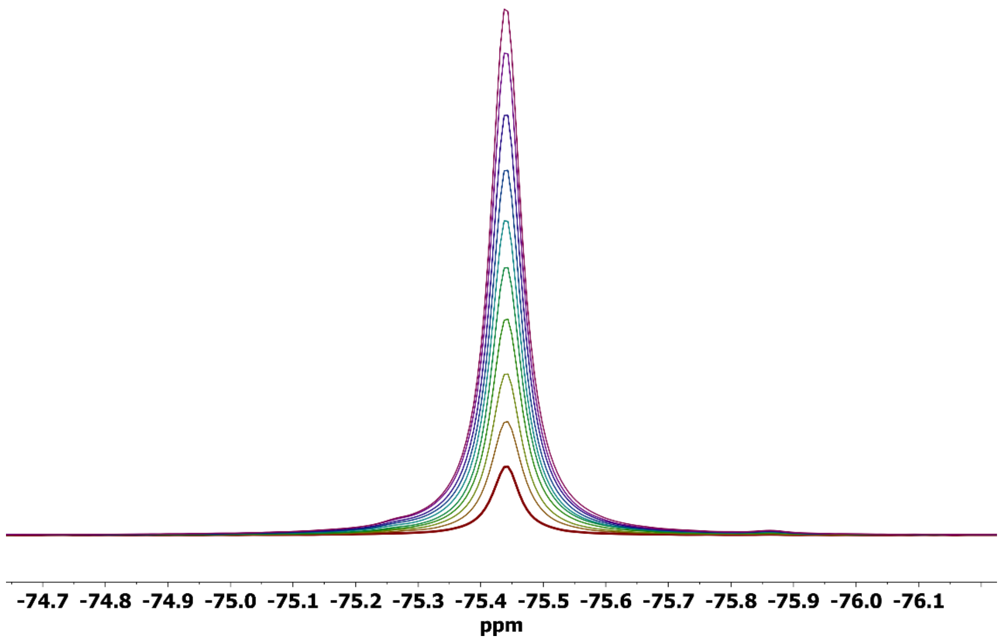

Figure S 63: ^{19}F NMR spectra of titration of $[Fe(L2)]^{2+}$ and trifluorolactate. Conditions: 5 mM complex, 1 -10 mM trifluorolactate, pD 7.8, D_2O, 0.1 M NaCl.

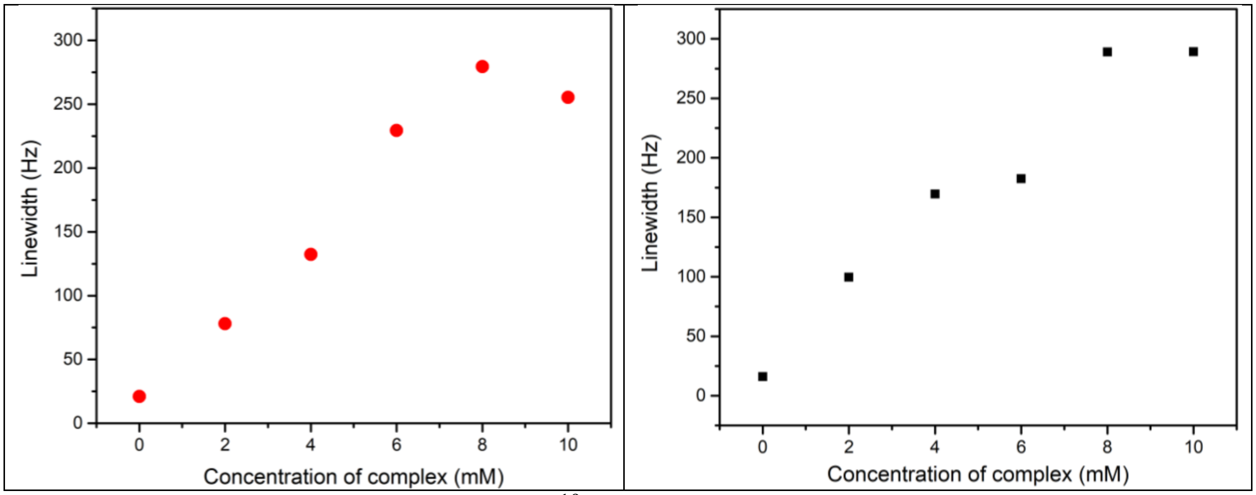

Figure S 64: Plot of change in linewidth of ^{19}F NMR vs concentration from the titration of $[Co(L1)]^+$ with trifluorolactate. D_2O (left) and H_2O (right).

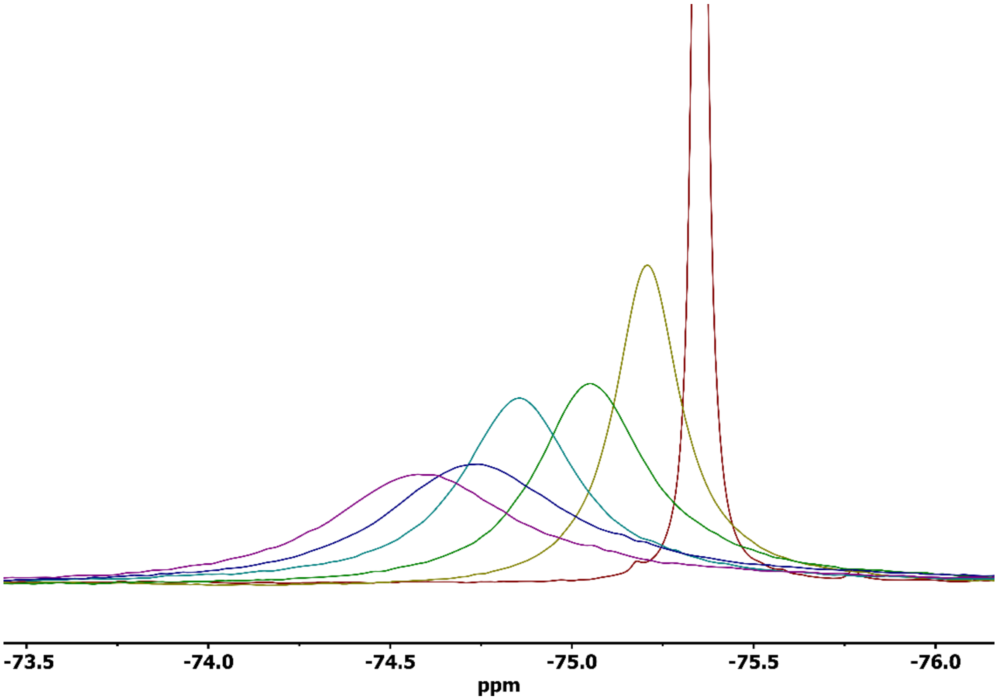

Figure S 65: ^{19}F NMR spectra for titration of trifluorolactate with $[Co(L1)]^+$. Conditions: 0 – 10 mM complex, 10 mM trifluorolactate, pH 7.4, H_2O, 0.1 M NaCl.

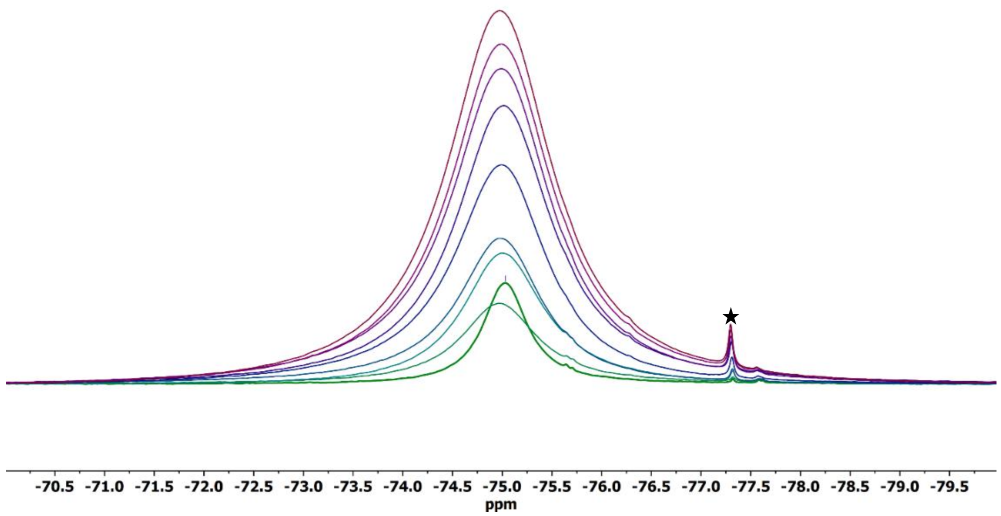

Figure S 66: ^{19}F NMR spectra of titration of $[Co(L1)]^{+}$ and trifluorolactate. Conditions: 10 mM complex, 10 -100 mM trifluorolactate, pD 7.8, D_2O, 0.1 M NaCl. Impurity in trifluorolactate is marked by a star.

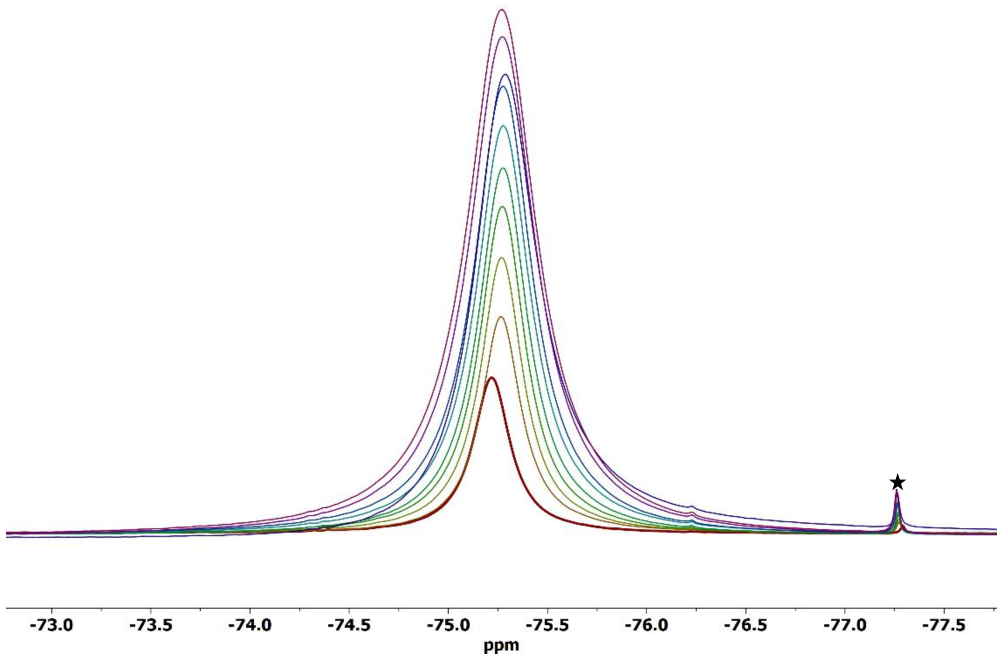

Figure S 67: ^{19}F NMR spectra of titration of $[Co(L2)]^{2+}$ and trifluorolactate. Conditions: 10 mM complex, 10 -100 mM trifluorolactate, pD 7.8, D_2O, 0.1 M NaCl. Impurity in trifluorolactate is marked by a star.

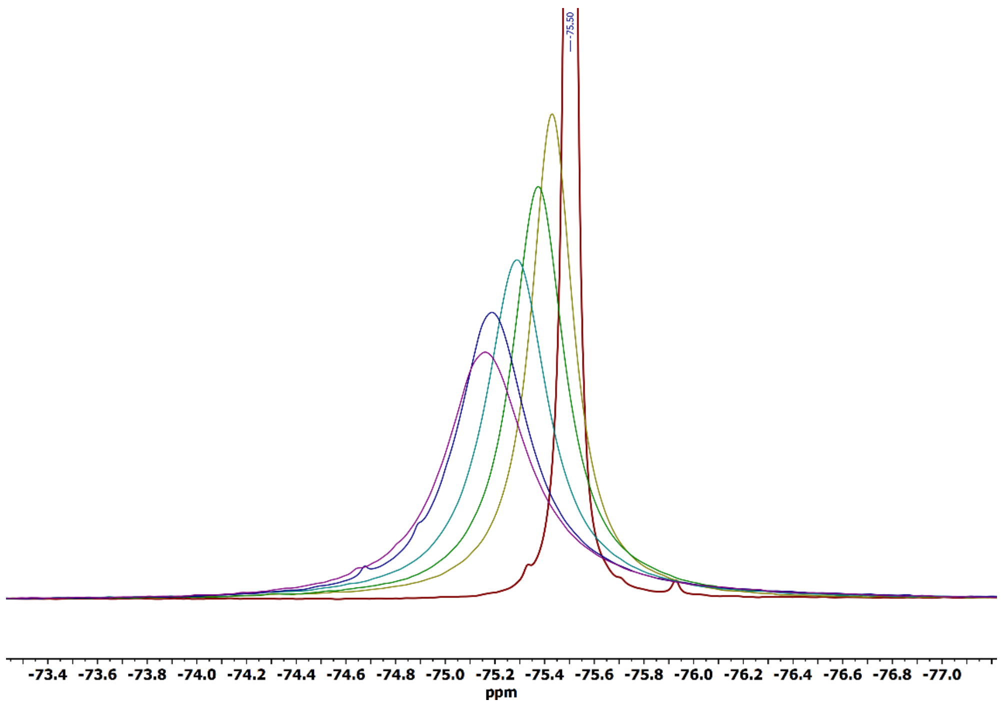

Figure S 68: ^{19}F NMR titration of 10 mM trifluorolactate with $[Co(L3)]^{2+}$. Conditions: complex 0 – 10 mM, pD 7.8, D_2O, 0.1 M NaCl.

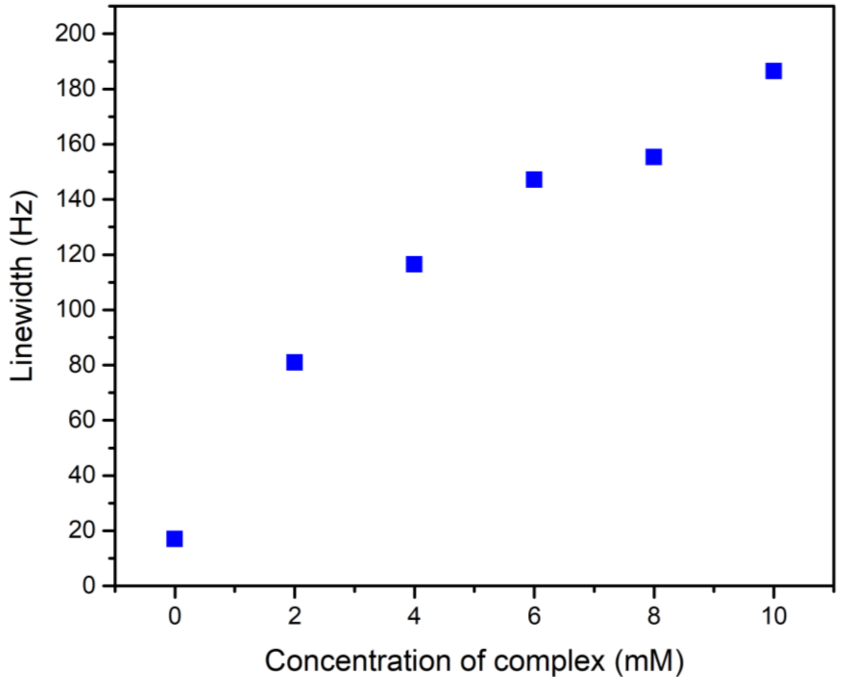

Figure S 69: Plot of change in linewidth of ^{19}F NMR vs concentration from the titration of $[Co(L3)]^{2+}$ with trifluorolactate.

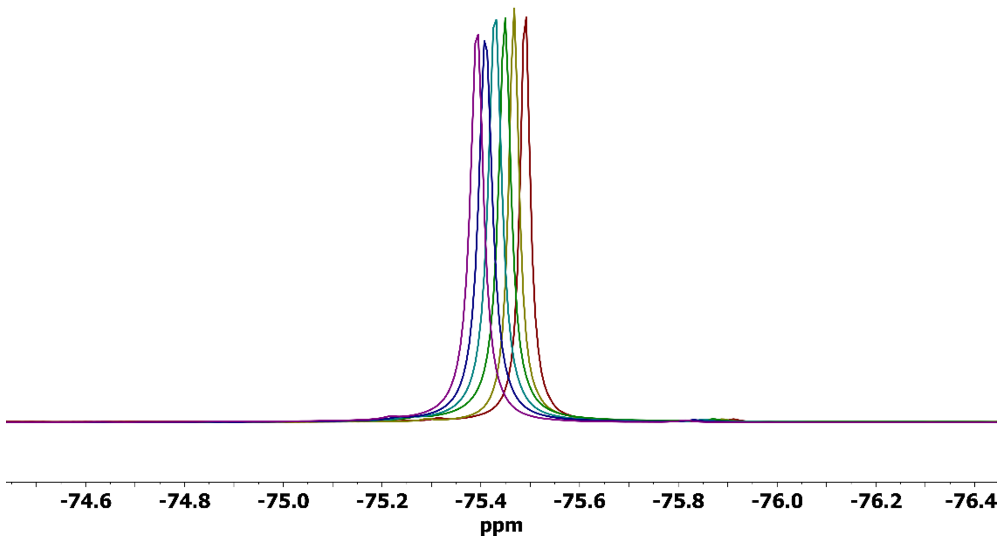

Figure S 70: ^{19}F NMR titration of 10 mM trifluorolactate with $[Fe(L4)]^{2+}$. Conditions: complex 0 – 10 mM, pD 7.8, D_2O, 0.1 M NaCl.

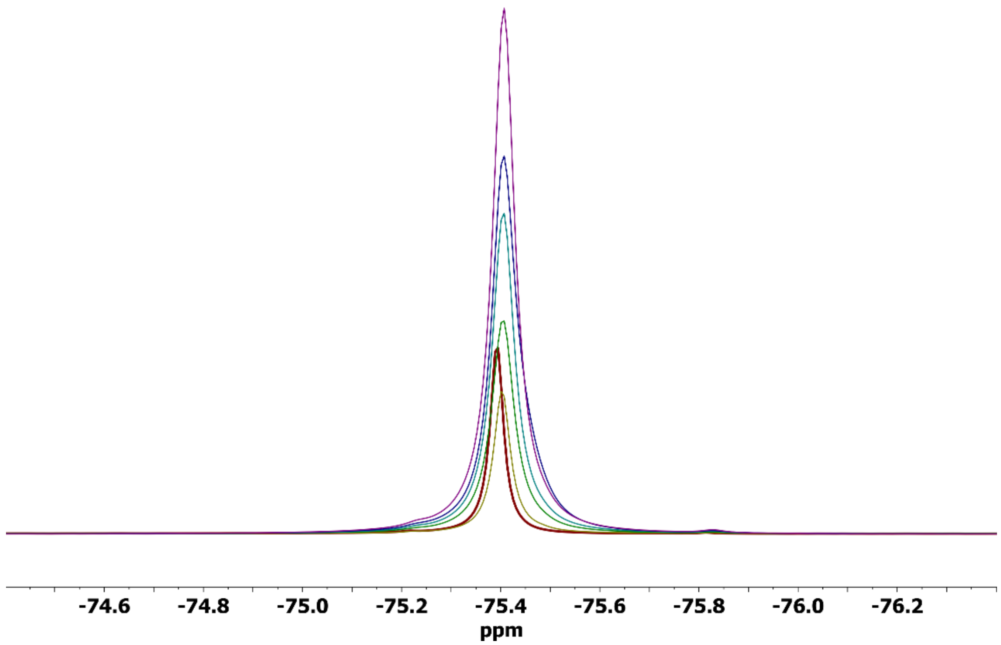

Figure S 71: ^{19}F NMR spectra of titration of $[Fe(L4)]^{2+}$ and trifluorolactate. Conditions: 10 mM complex, 10 -100 mM trifluorolactate, pD 7.8, D_2O, 0.1 M NaCl.

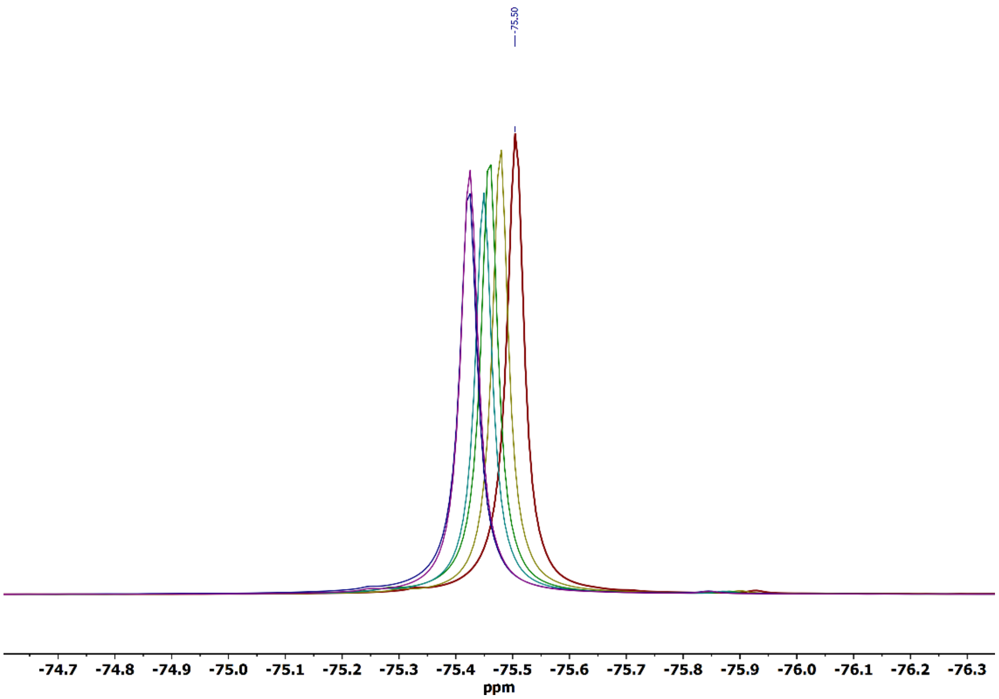

Figure S 72: ^{19}F NMR titration of 10 mM trifluorolactate with $[Co(L4)]^{2+}$. Conditions: complex 0 – 10 mM, pD 7.8, D_2O, 0.1 M NaCl.

Appendix

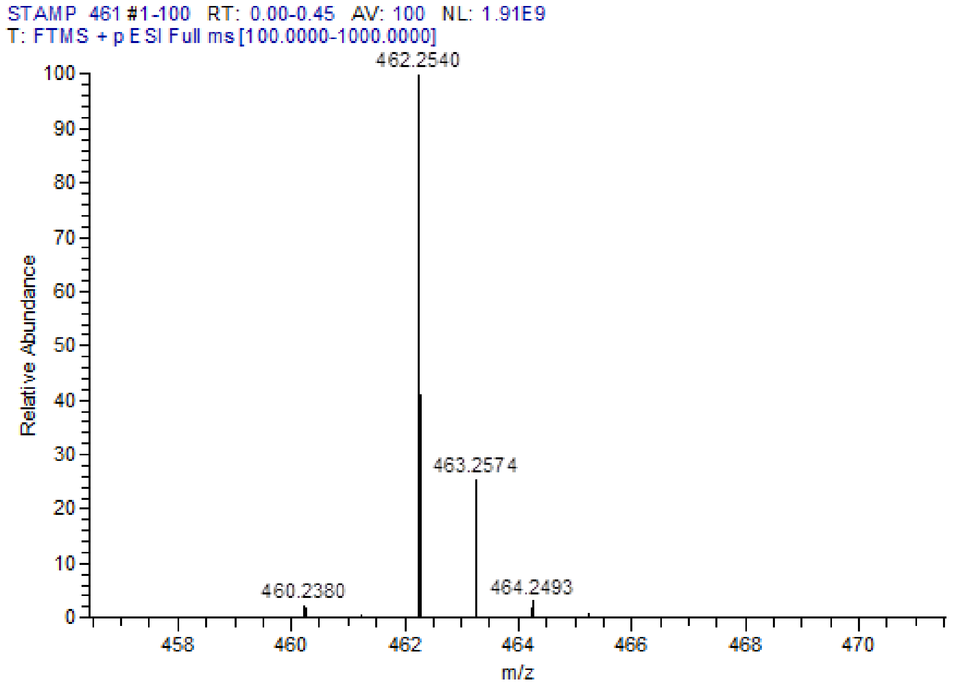

Figure S 73: High-resolution mass spectrometry of L1 ligand.

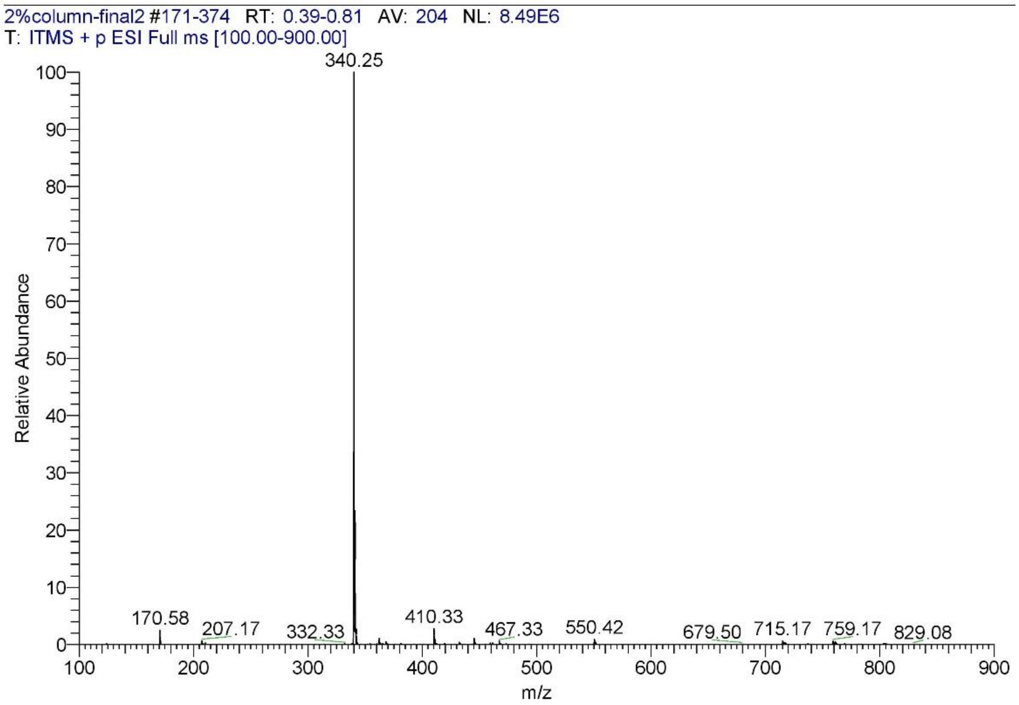

Figure S 74: High-resolution mass spectrometry of L3 ligand.

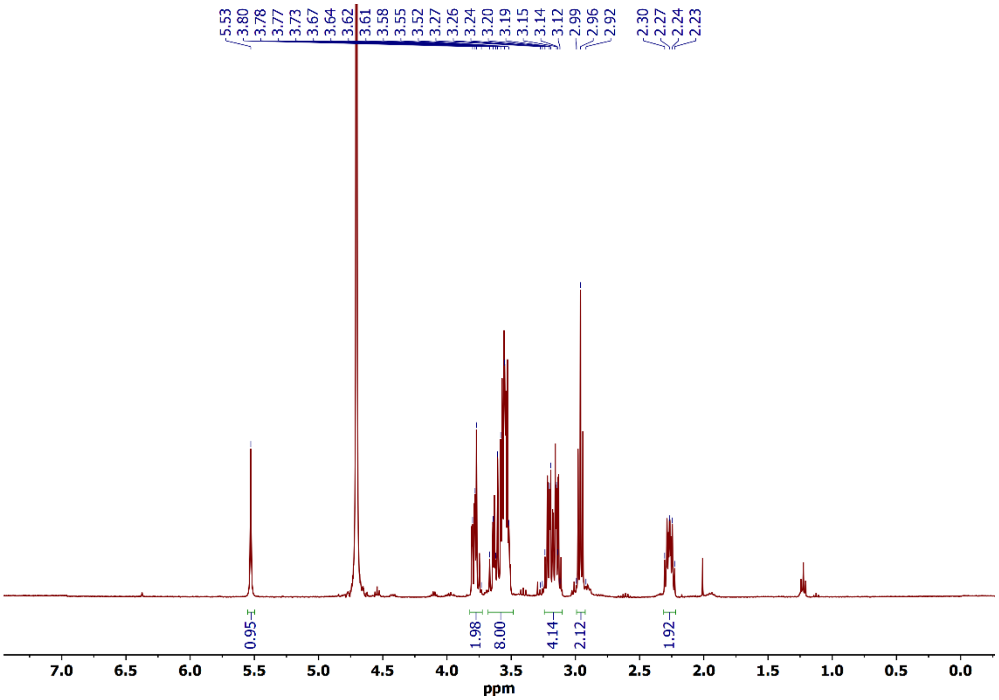

Figure S 75: ^{1}H NMR spectrum of Protected sulfone TACN in D_2O. Conditions: 20mM

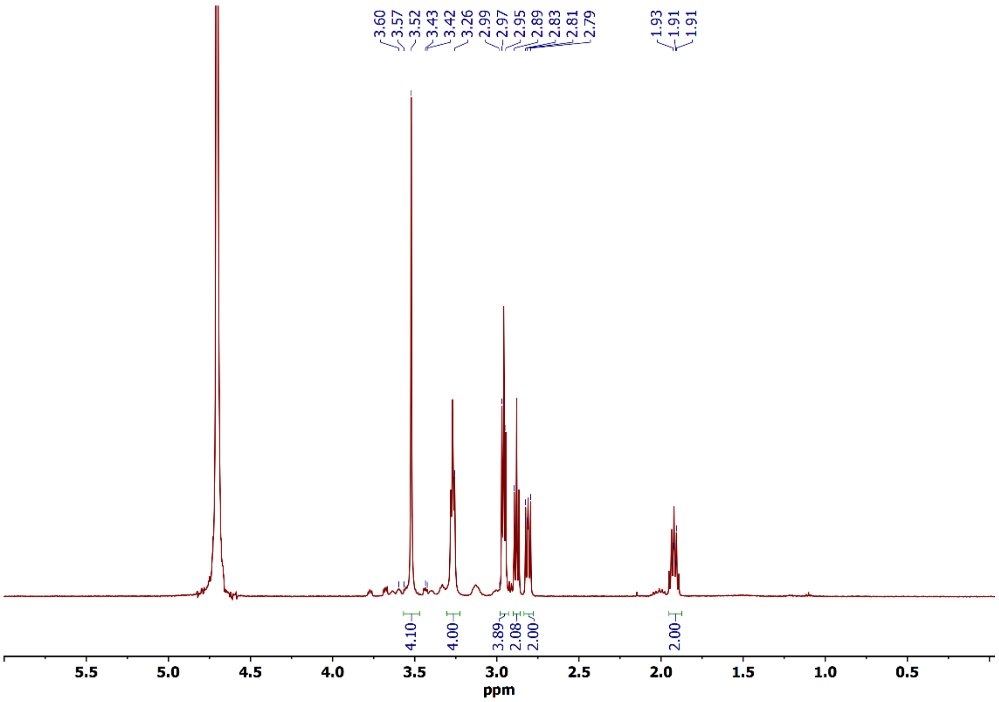

Figure S 76: ^{1}H NMR spectrum of sulfone TACN in D_2O. Conditions: 20mM

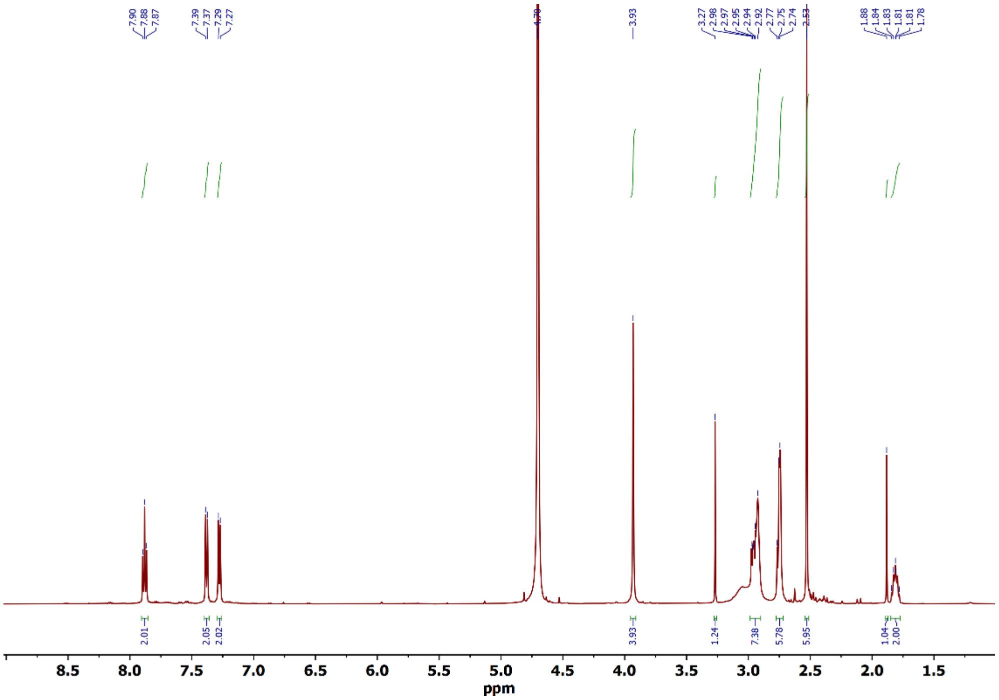

Figure S 77: ^{1}H NMR spectrum of L1 ligand in D_2O. Conditions: 20mM

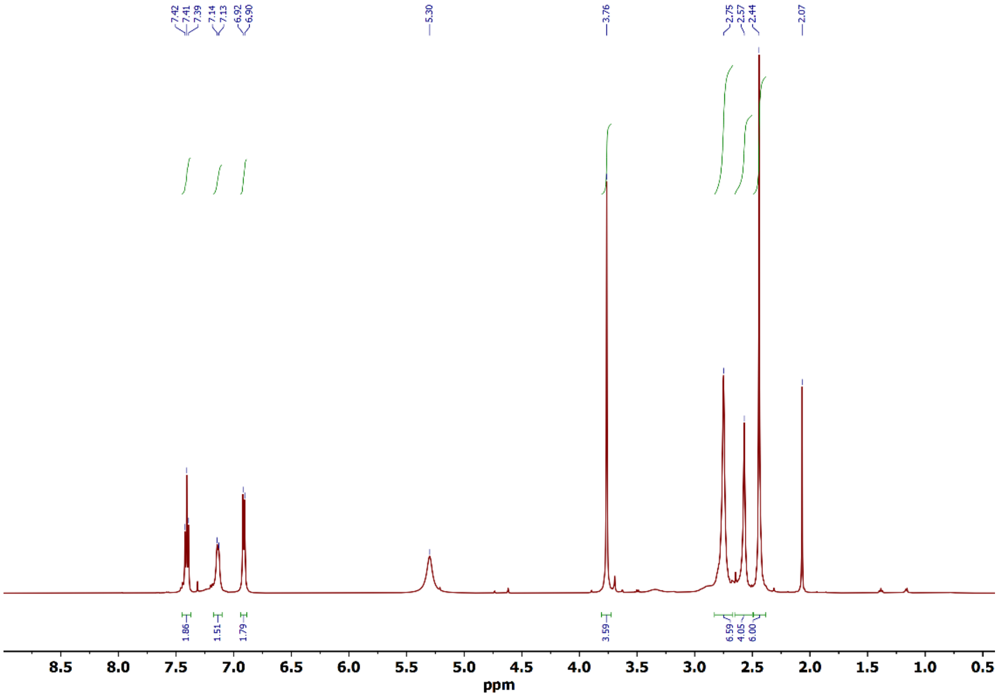

Figure S 78: ^{1}H NMR spectrum of L3 ligand in $CDCl_3$. Conditions: 20mM

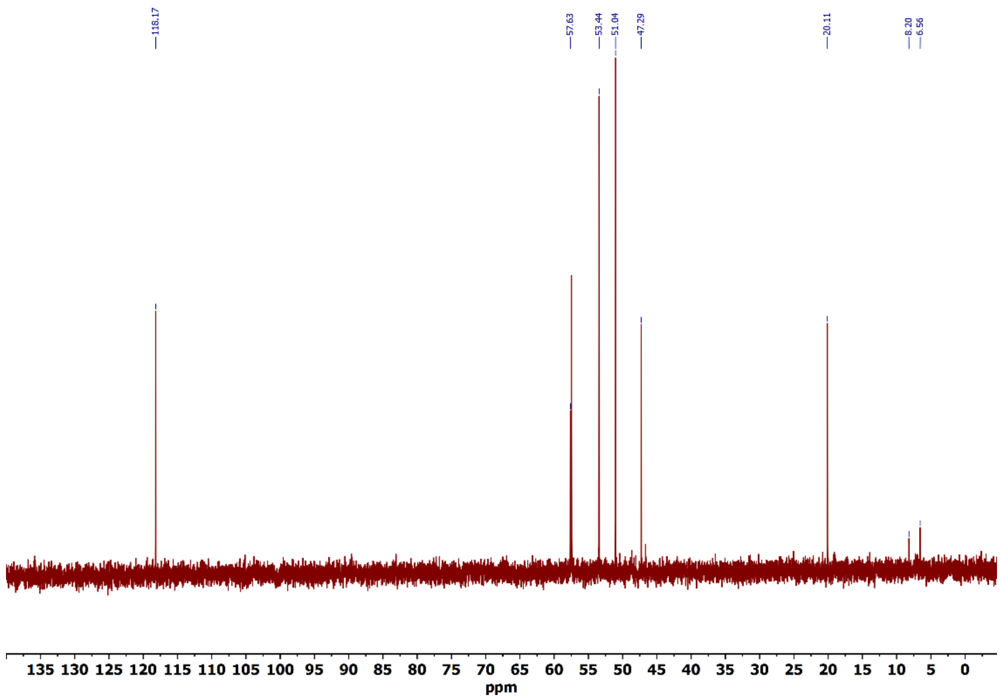

Figure S 79: ^{13}C NMR spectrum of Protected sulfone TACN in D_2O. Conditions: 20mM

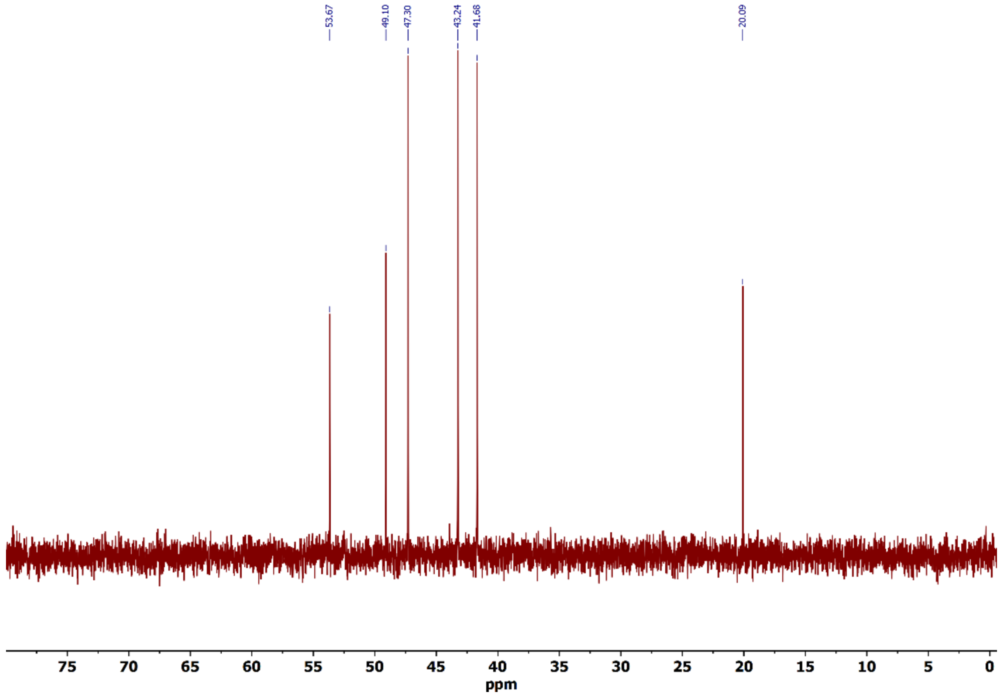

Figure S 80: ^{13}C NMR spectrum of sulfone TACN in D_2O. Conditions: 20mM

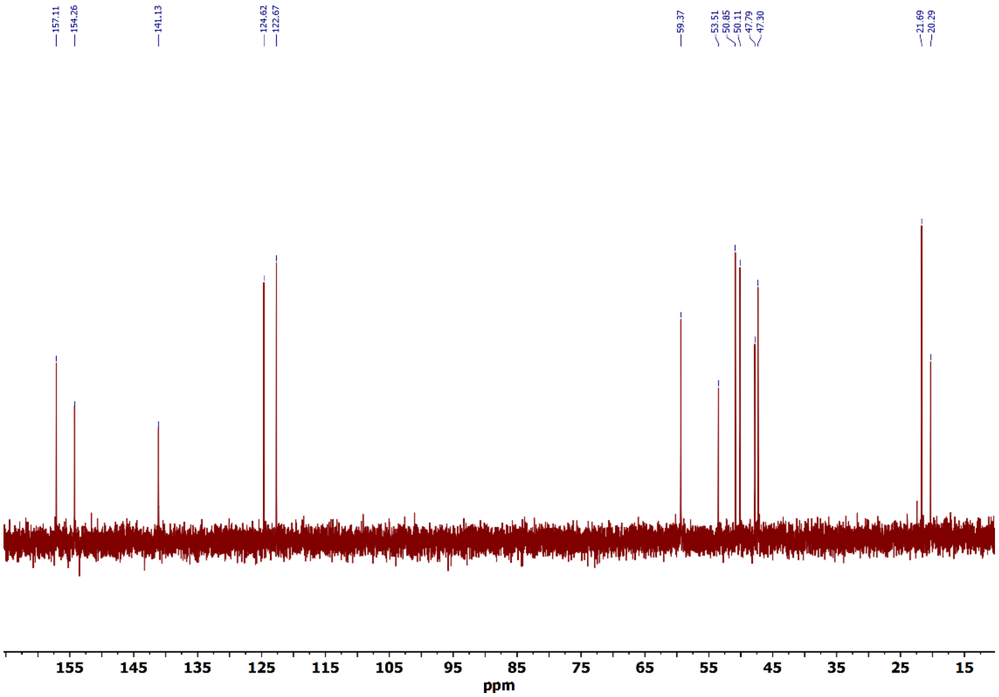

Figure S 81: ^{13}C NMR spectrum of L1 ligand in D_2O. Conditions: 20mM

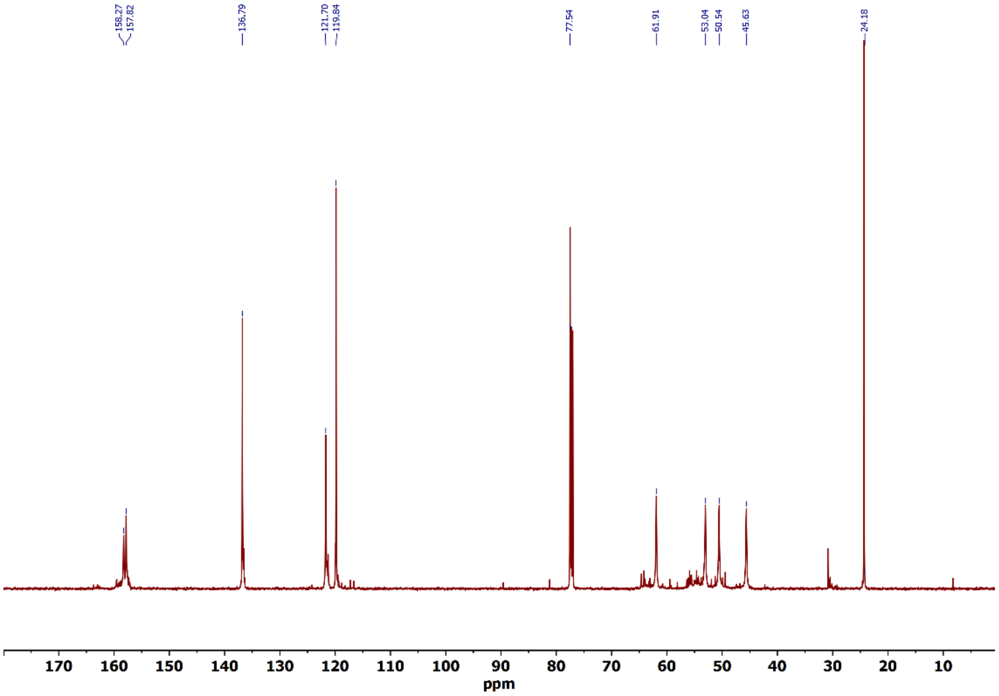

Figure S 82: ^{13}C NMR spectrum of L3 ligand in $CDCl_3$. Conditions: 20mM

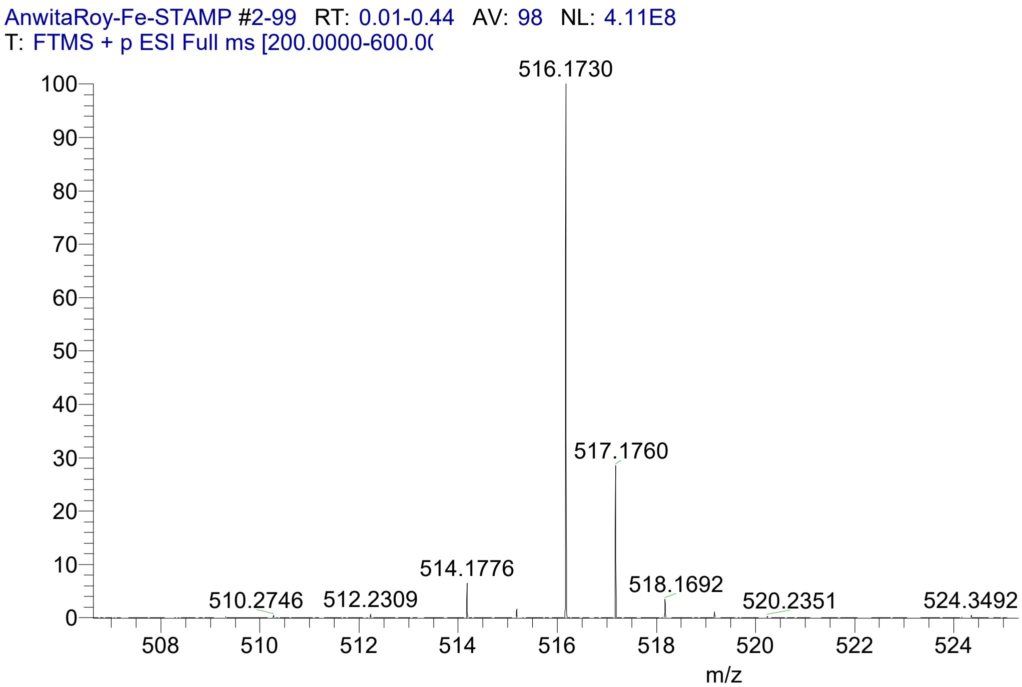

Figure S 83: High-resolution mass spectrometry of $[Fe(L1)]^+$.

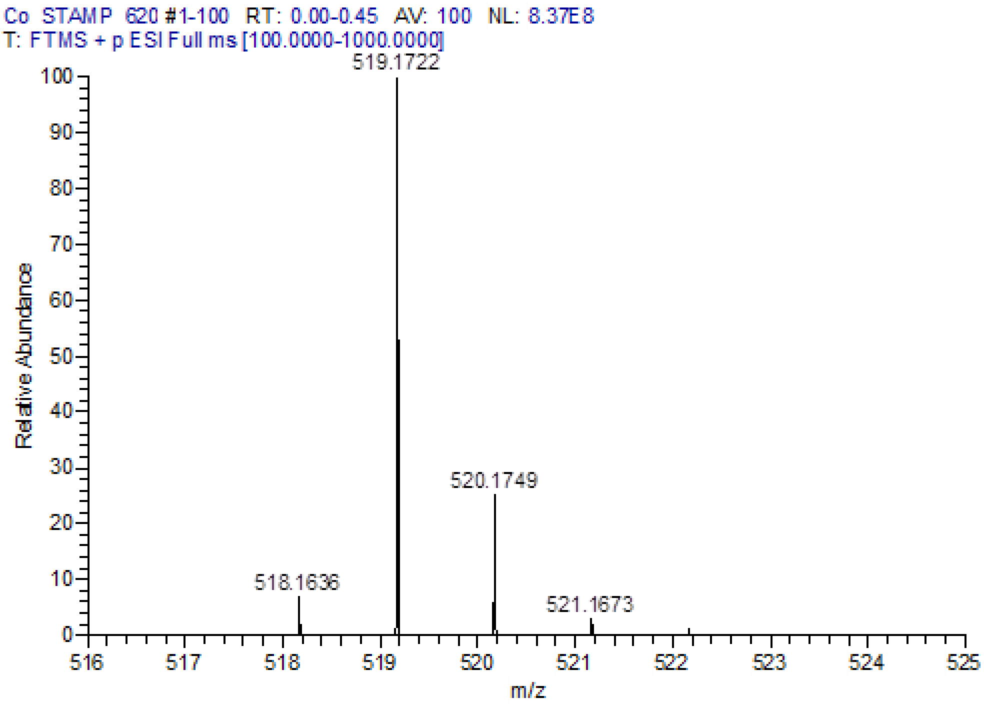

Figure S 84: High-resolution mass spectrometry of $[Co(L1)]^+$.

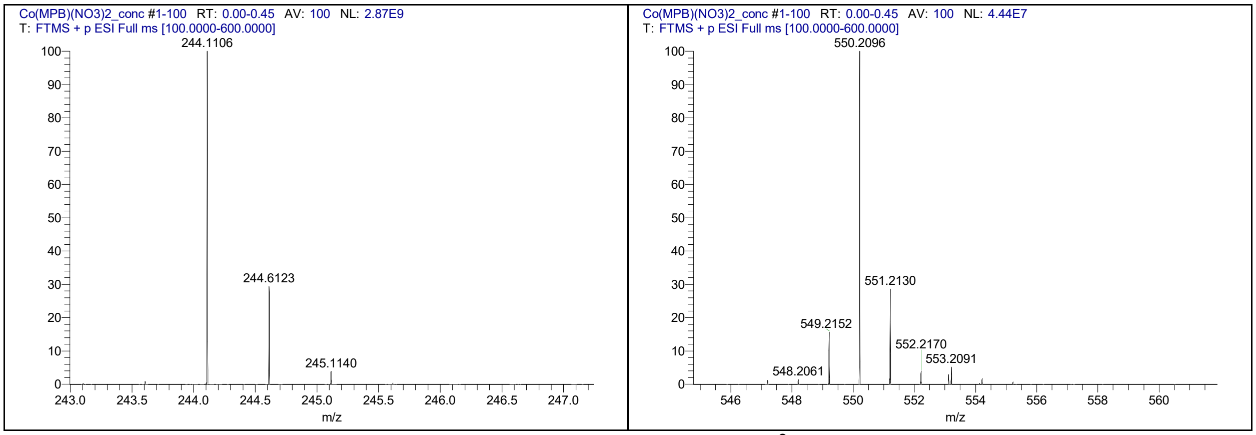

Figure S 85: High-resolution mass spectrometry of $[Co(L2)]^{2+}$.

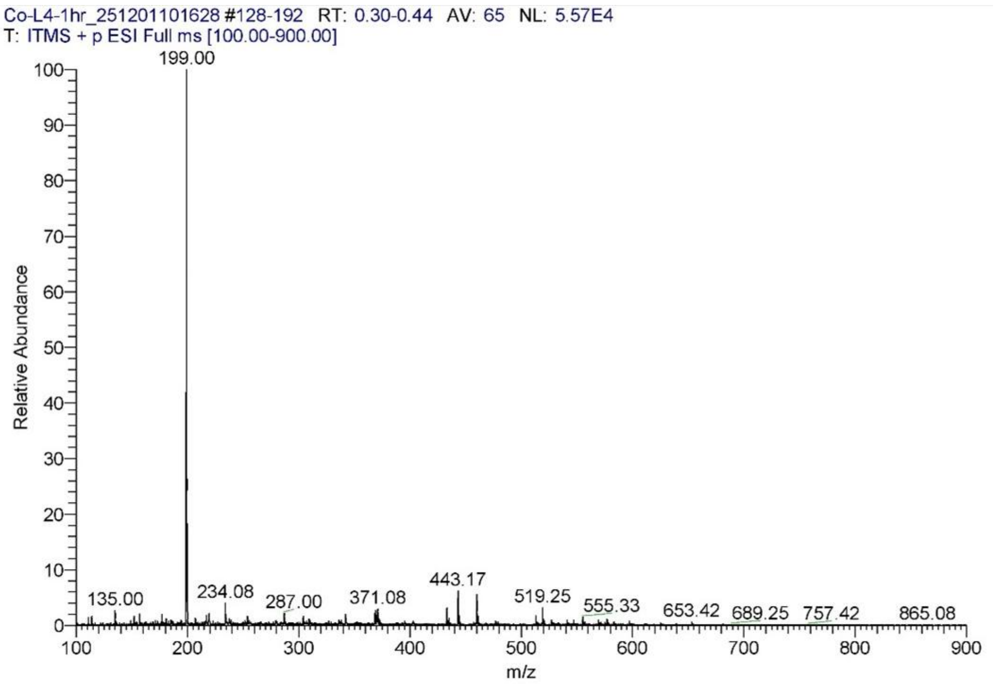

Figure S 86: High-resolution mass spectrometry of $[Co(L3)]^{2+}$.

(1) Schubert, E. M. Utilizing the Evans Method with a Superconducting Nmr Spectrometer in the Undergraduate Laboratory. *J Chem Educ* **1992**, *69* (1), 62.
(2) Balaji, D. K.; Spernyak, J. A.; Crawley, M. R.; Morrow, J. R. Redox-responsive Fe(II)/Fe(III) MRI probes with pentadentate or hexadentate macrocyclic ligands. *Inorg Chem Front* **2026**. DOI: 10.1039/d6qi00660d.
